# Magnetic Barkhausen Noise Sensor: A Comprehensive Review of Recent Advances in Non-Destructive Testing and Material Characterization

**DOI:** 10.3390/s26010258

**Published:** 2025-12-31

**Authors:** Polyxeni Vourna, Pinelopi P. Falara, Aphrodite Ktena, Evangelos V. Hristoforou, Nikolaos D. Papadopoulos

**Affiliations:** 1 National Centre for Scientific Research “Demokritos”, Institute of Nanoscience and Nanotechnology, 15341 Agia Paraskevi, Greece; 2 School of Chemical Engineering, National Technical University of Athens, 9 Iroon Polytechniou Str., Zografou, 15772 Athens, Greece; pin.falara@gmail.com; 3 General Department, National and Kapodistrian University of Athens, 15784 Athens, Greece; apktena@uoa.gr; 4 Institute of Communication and Computer Systems, 15773 Athens, Greece; hristoforou@ece.ntua.gr; 5 Department of Research and Development, BFP Advanced Technologies G.P., 11633 Athens, Greece; npapadopoulos@bfp-tech.com

**Keywords:** Barkhausen noise, non-destructive testing, magnetic sensors, materials characterization, domain wall dynamics, machine learning, structural health monitoring

## Abstract

Magnetic Barkhausen noise (MBN) represents a powerful non-destructive testing and material characterization methodology enabling quantitative assessment of microstructural features, mechanical properties, and stress states in ferromagnetic materials. This comprehensive review synthesizes recent advances spanning theoretical foundations, sensor design, signal processing methodologies, and industrial applications. The physical basis rooted in domain wall dynamics and statistical mechanics provides rigorous frameworks for interpreting MBN signals in terms of grain structure, dislocation density, phase composition, and residual stress. Contemporary instrumentation innovations including miniaturized sensors, multi-parameter systems, and high-entropy alloy cores enable measurements in challenging environments. Advanced signal processing techniques—encompassing time-domain analysis, frequency-domain spectral methods, time–frequency transforms, and machine learning algorithms—extract comprehensive material information from raw Barkhausen signals. Deep learning approaches demonstrate superior performance for automated material classification and property prediction compared to traditional statistical methods. Industrial applications span manufacturing quality control, structural health monitoring, railway infrastructure assessment, and predictive maintenance strategies. Key achievements include establishing quantitative correlations between material properties and stress states, with measurement uncertainties of ±15–20 MPa for stress and ±20 HV for hardness. Emerging challenges include standardization imperatives, characterization of advanced materials, machine learning robustness, and autonomous system integration. Future developments prioritizing international standards, physics-informed neural networks, multimodal sensor fusion, and wireless monitoring networks will accelerate industrial adoption supporting safe, efficient engineering practice across diverse sectors.

## 1. Introduction

### 1.1. Overview of Magnetic Barkhausen Noise

The magnetic Barkhausen noise (MBN) technique represents a fundamental and widely utilized non-destructive testing (NDT) and electromagnetic characterization method applicable to ferromagnetic materials. Since its original discovery in 1919 by Heinrich Barkhausen [1], this phenomenon has evolved from a physical curiosity into a sophisticated and practical analytical tool for material evaluation across diverse scientific and industrial applications. The technique operates through the detection and analysis of electromagnetic signals generated during the complex processes of magnetization reversal within ferromagnetic materials, offering unique capabilities for assessing microstructural features and mechanical states that are inaccessible to conventional testing approaches.

The physical foundation of Barkhausen noise lies in the discontinuous motion of magnetic domain walls within ferromagnetic materials when exposed to a time-varying magnetic field. When an external magnetic field drives these domain walls through a material containing structural heterogeneities—such as grain boundaries, dislocations, or precipitates—the walls become pinned and subsequently depinned in abrupt events. This jerky, avalanche-like motion generates measurable electromagnetic signals characterized by transient voltage pulses, collectively termed Barkhausen noise [2]. The irreversible nature of domain wall displacement, coupled with the stochastic distribution of pinning sites throughout the material microstructure, produces the distinctive noise-like signal from which the method derives its name.

From a measurement perspective, MBN acquisition employs inductive sensing principles wherein a primary excitation coil generates the magnetic field driving magnetization reversal, while a secondary pickup coil detects the electromagnetic signals arising from domain wall motion. The frequency selectivity of this approach, typically concentrating sensitivity in the range of 100 kHz to several MHz, facilitates depth-profiling capabilities through the electromagnetic skin effect. This frequency dependence permits investigation of material properties at various distances beneath the sample surface—a capability particularly valuable for evaluating case-hardened components, surface treatments, and subsurface microstructural modifications [3]. Environmental considerations including temperature effects, humidity, and electromagnetic interference require careful attention to ensure measurement reproducibility and quantitative accuracy.

Recent advances have positioned MBN at the forefront of modern materials science and engineering through the development of automated measurement systems amenable to real-time implementation. Multi-parameter sensor systems capable of simultaneous acquisition across diverse frequency bands enhance information content, permitting discriminatory analysis of complex material states where stress and microstructural effects simultaneously influence mechanical properties. Machine learning methodologies have emerged as powerful complementary analysis approaches, enabling the extraction of subtle diagnostic information from raw Barkhausen signals without explicit feature engineering [4]. Deep learning architectures, particularly convolutional neural networks applied to time–frequency representations of MBN data, demonstrate remarkable capability for automated material classification and property prediction tasks.

The applicability of MBN encompasses diverse industrial sectors spanning aerospace quality assurance, railway infrastructure monitoring, manufacturing process control, and structural health monitoring of in-service components [5]. The detection of grinding-induced thermal damage, evaluation of case-depth consistency in hardened components, and assessment of shot-peening effects represent well-established practical applications [6,7]. Emerging applications in additive manufacturing process monitoring and characterization of advanced materials extend the technique’s scope toward future manufacturing paradigms [8,9]. The versatility and non-destructive nature of MBN analysis make it an indispensable tool for both research investigations into fundamental materials behavior and practical engineering applications requiring reliable, reproducible, and rapid assessment of material properties and integrity.

### 1.2. Historical Development and Theoretical Background

The theoretical foundations of magnetic Barkhausen noise trace their origins to the pioneering work on ferromagnetism conducted during the early 20th century. The conceptual framework emerged from the landmark contributions of Pierre Weiss, who in 1907 proposed the domain theory of ferromagnetism, introducing the molecular field hypothesis to explain spontaneous magnetization in ferromagnetic materials [10]. Weiss postulated that ferromagnetic materials subdivide into microscopic regions termed magnetic domains, within which atomic magnetic moments align spontaneously due to an internal molecular field proportional to magnetization. This revolutionary concept provided the first theoretical explanation for the coexistence of strong internal magnetic ordering with macroscopically demagnetized states observed in ferromagnetic specimens.

The domain theory advanced substantially through the complementary contributions of Felix Bloch and Louis Néel, who elucidated the internal structure and energetics of domain boundaries. Bloch demonstrated that magnetization transitions between adjacent domains occur gradually across finite-width boundaries—now termed Bloch walls—where magnetic moments rotate progressively to minimize exchange energy while accommodating magnetocrystalline anisotropy [11,12]. Néel extended these concepts by identifying alternative wall configurations and exploring antiferromagnetic ordering, establishing that domain wall width represents an equilibrium between competing exchange and anisotropy energy contributions [13]. These foundational insights established the physical basis for understanding the discontinuous magnetization processes that generate Barkhausen noise signals.

Throughout the mid-to-late 20th century, theoretical understanding evolved substantially through experimental validation and refinement of domain wall dynamics. Direct observations via magneto-optical techniques confirmed the existence of discrete domain structures and revealed the jerky, avalanche-like nature of domain wall motion under applied magnetic fields [14]. Recognition that structural heterogeneities—including grain boundaries, dislocations, precipitates, and compositional variations—create pinning sites that impede domain wall motion established the microstructural sensitivity underlying practical Barkhausen noise applications [2]. The stochastic nature of pinning and depinning events, coupled with the collective interactions among domain walls, produces the characteristic noise-like electromagnetic signals detected during magnetization processes.

A pivotal advancement enabling quantitative analysis emerged through the development of the Jiles–Atherton model for magnetic hysteresis. Introduced in 1986, this phenomenological theory provides a comprehensive framework relating macroscopic magnetization to applied magnetic field through explicit consideration of domain wall pinning, interdomain coupling, and reversible magnetization processes [15]. The model formulates anhysteretic magnetization—representing the equilibrium state absent pinning effects—through a modified Langevin function incorporating mean-field coupling between domains. Hysteresis arises through the introduction of a pinning parameter characterizing energy dissipation during irreversible domain wall displacement [15,16]. Extensions incorporating stress effects, two-phase materials, and anisotropic media have substantially broadened applicability, enabling the prediction of Barkhausen noise characteristics under diverse conditions [16,17]. The physically meaningful parameters and computational efficiency of the Jiles–Atherton framework continue supporting both fundamental investigations and practical sensor development for magnetic material characterization.

Parallel theoretical developments elucidated the statistical mechanics underlying Barkhausen avalanche phenomena. Recognition that ferromagnetic domain walls constitute driven interfaces in disordered media connected Barkhausen noise to broader classes of critical phenomena exhibiting power-law scaling [6]. Mean-field theories and renormalization group approaches demonstrated that avalanche size and duration distributions follow universal scaling relations characterized by critical exponents depending on system dimensionality, disorder type, and interaction range [6,18]. Experimental verification of predicted scaling behaviors in diverse ferromagnetic materials validated these statistical frameworks, establishing Barkhausen noise as a model system for studying nonequilibrium phase transitions and crackling noise phenomena [19]. The interplay between dipolar interactions, elastic effects, and quenched disorder determines universality classes, with distinct exponents characterizing soft magnetic materials versus hardened specimens exhibiting enhanced pinning [20].

Recent developments integrating machine learning and artificial intelligence methodologies represent a transformative advancement in Barkhausen noise analysis and interpretation. Traditional approaches relying on manually engineered features—such as root-mean-square voltage, spectral parameters, or pulse counts—increasingly yield to data-driven algorithms capable of extracting complex, nonlinear relationships directly from raw signals [4,21]. Deep learning architectures, particularly convolutional neural networks applied to time–frequency representations generated via short-time Fourier transforms or wavelet decompositions, demonstrate remarkable capability for automated material classification and property prediction without requiring explicit feature engineering [4]. Recurrent neural networks and long short-term memory architectures exploit temporal dependencies within Barkhausen sequences, achieving superior stress prediction accuracy compared to conventional regression approaches [22]. Uncertainty quantification through Bayesian neural networks enables probabilistic predictions incorporating both measurement noise and model uncertainties, enhancing reliability for critical applications [23]. Ensemble methods combining multiple algorithms improve robustness, while transfer learning facilitates application to new materials with limited calibration data [21]. These artificial intelligence methodologies align directly with contemporary sensor technology trends emphasizing autonomous operation, real-time decision-making, and integration within Industry 4.0 frameworks, positioning magnetic Barkhausen noise analysis at the forefront of intelligent non-destructive testing and material characterization systems.

The convergence of century-spanning theoretical developments—from Weiss’s molecular field concept through statistical avalanche theories to contemporary artificial intelligence integration—establishes magnetic Barkhausen noise as a mature yet continually evolving field combining fundamental physics with practical sensor applications aligned with the mission of advancing sensor science and technology.

### 1.3. Scope and Objectives of Review

This comprehensive review addresses the multifaceted dimensions of magnetic Barkhausen noise (MBN) as a sensor technology and non-destructive testing (NDT) methodology, with particular emphasis on synthesizing recent developments spanning the last decade and projecting critical research directions for the coming years. The *Sensors* journal’s mission to advance sensor science and technology, foster interdisciplinary connections, and bridge fundamental research with practical applications aligns precisely with the scope of this review, which integrates theoretical foundations, instrumentation innovations, signal processing methodologies, and diverse application domains within a cohesive framework.

The period from approximately 2015 to 2025 has witnessed transformative advances in MBN research, driven by convergent developments in experimental instrumentation, computational methodologies, and machine learning integration [24]. Significant progress has been achieved in the standardization and reproducibility of MBN measurements, addressing longstanding challenges regarding interlaboratory comparison and measurement consistency [24]. Automated measurement systems incorporating sophisticated signal conditioning and digital processing have substantially improved data quality and operational efficiency, facilitating integration into industrial quality control frameworks [25]. The emergence of miniaturized, low-power sensor architectures demonstrates feasibility for embedded structural health monitoring applications requiring integration in harsh or inaccessible environments [26]. Contemporary research has expanded the characterization capabilities of MBN beyond traditional applications, establishing quantitative relationships between magnetic parameters and microstructural features, mechanical properties, and residual stress distributions through systematic parameter analysis and multivariate statistical approaches [27,28].

Theoretical foundations (Section 2) provide rigorous frameworks for MBN interpretation through domain wall dynamics and statistical avalanche models [2,6]. Contemporary theoretical developments integrate phenomenological hysteresis models—particularly the Jiles–Atherton framework—with micromagnetic simulations and mean-field approximations to predict noise characteristics under diverse material conditions and magnetic excitations [15,16]. Instrumentation innovations extend beyond conventional sensing coil configurations toward multi-parameter measurement systems combining MBN with complementary magnetic techniques including incremental permeability analysis and harmonic distortion measurement, thereby enriching information content for material characterization [29]. Modern signal processing methodologies capitalize on time–frequency analysis techniques—including short-time Fourier transforms, wavelet decompositions, and empirical mode decompositions—to extract microstructurally sensitive parameters inaccessible through traditional time- or frequency-domain analysis alone [4,27]. Comprehensive application surveys demonstrate MBN’s utility across manufacturing quality assurance, aerospace and railway maintenance, electrical steel characterization, additive manufacturing process monitoring, and emerging structural health monitoring paradigms [27,28,30].

This review systematically addresses several critical research questions currently motivating MBN investigations: (1) Standardization and reproducibility: How can measurement protocols achieve reproducibility comparable to conventional mechanical testing while accommodating diverse equipment architectures and operational environments? (2) Multi-parameter decoupling: Can quantitative discrimination between competing microstructural effects—grain size, dislocation density, precipitation state—be achieved alongside stress state determination from single-sensor measurements? (3) Depth profiling and subsurface sensitivity: What electromagnetic mechanisms govern the effective sampling depth, and can frequency-dependent excitation strategically modulate depth sensitivity for non-invasive subsurface characterization? (4) Material dependence of noise scaling: How do avalanche size and duration distributions vary across material classes, microstructural states, and thermomechanical histories, and what universal scaling laws govern these variations? (5) Machine learning integration: Can data-driven algorithms extract microstructural information superior to physics-guided feature engineering, and what strategies optimize training data efficiency for transferability across material systems? (6) Integration with Industry 4.0: How can autonomous MBN systems integrate within digital manufacturing ecosystems to enable real-time quality feedback and adaptive process control [31]?

Contemporary MBN methodologies exhibit both well-established strengths and persistent limitations warranting critical scrutiny. The non-destructive nature and surface-sensitive depth range represent distinct advantages for quality assurance and condition monitoring applications [24]. However, the methodology demonstrates inherent sensitivity to measurement parameters including magnetization frequency, waveform shape, excitation amplitude, sensor positioning, and environmental conditions—factors complicating standardization and cross-laboratory comparison [24]. The measurement repeatability challenges, particularly for rough or curved surfaces encountered in advanced manufacturing contexts, necessitate continued instrumentation refinement to achieve the surface conformability and gauge volume consistency required for reliable quantitative assessment [30]. Signal-to-noise ratio limitations in certain material conditions—particularly low-permeability, heavily hardened, or finely microstructured systems exhibiting suppressed Barkhausen activity—restrict applicability and require enhanced signal conditioning strategies [28]. Furthermore, the passive Barkhausen phenomenon’s stochastic nature introduces inherent uncertainty into individual measurements, necessitating careful threshold selection and statistical averaging protocols to extract robust quantitative parameters [27].

Future developments must address several interconnected challenges: (1) Standardization imperatives: The establishment of comprehensive international standards encompassing sensor calibration, magnetization protocols, signal processing algorithms, and quantitative interpretation criteria remains essential for industrial adoption [25]. (2) Emerging manufacturing paradigms: Application to additive manufacturing, advanced composites, and high-entropy alloys—material systems with limited existing MBN characterization—represents crucial research frontiers [30]. (3) High-frequency operation: Extension to GHz-range frequencies enabling investigation of fundamental domain wall dynamics and enhanced depth sensitivity for thick-section components requires novel instrumentation architectures [32]. (4) Machine learning robustness: The development of physics-informed neural networks incorporating domain knowledge to improve generalization, reduce overfitting, and enhance the interpretability of learned features represents a promising direction [4]. (5) Autonomous sensor systems: The integration of miniaturized MBN sensors with wireless communication, onboard data processing, and cloud connectivity to enable continuous structural health monitoring in distributed sensor networks aligns with Industry 4.0 objectives [31]. (6) Multimodal integration: The fusion of MBN measurements with complementary sensors (ultrasonic, thermal, acoustic emission) within unified frameworks may substantially enhance diagnostic capability for complex damage scenarios.

**Figure 1:**
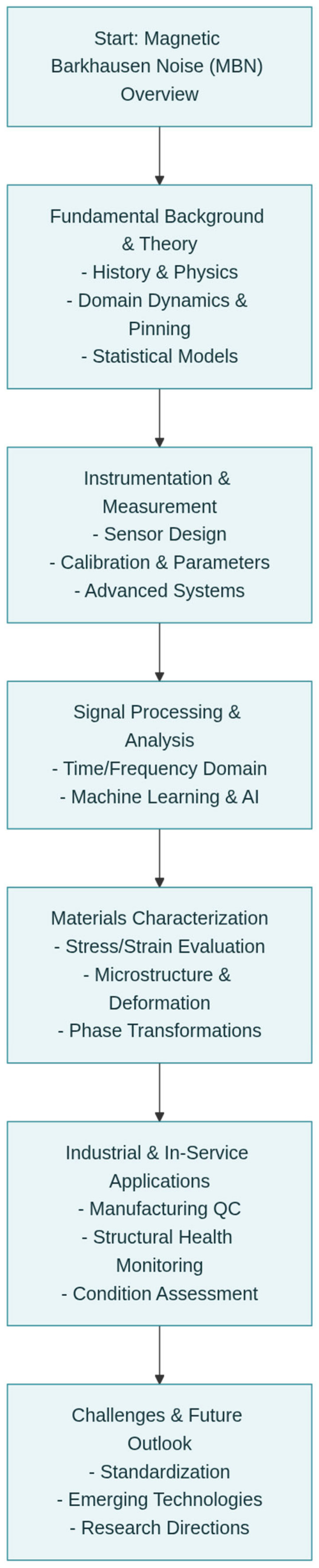
A schematic flowchart illustrating the organizational structure of this review. The manuscript progresses from the historical and theoretical foundations of magnetic Barkhausen noise (Section 2), through instrumentation and signal processing methodologies (Section 3, Section 4, Section 5 and Section 6), to material characterization techniques and industrial applications (Section 7 and Section 8), concluding with current challenges and future research directions (Section 9).

To facilitate navigation through the comprehensive topics covered in this review, Figure 1 provides a schematic overview of the manuscript’s organization. The structure follows a logical progression from fundamental physical principles to practical implementation and future outlooks.

## 2. Theoretical Foundations

### 2.1. Barkhausen Effect Physics

The Barkhausen effect represents a quintessential example of crackling noise arising from avalanche-like magnetization discontinuities in ferromagnetic materials, and its physics encompasses a sophisticated interplay between structural disorder, long-range magnetic interactions, and critical phenomena [33]. Understanding the fundamental mechanisms underlying Barkhausen noise generation requires integrating concepts from statistical mechanics, micromagnetism, and materials science, providing insights applicable across diverse sensor and material characterization applications.

#### 2.1.1. Energy Landscape and Pinning Site Distributions

The energy landscape governing domain wall motion consists of competing potential contributions: exchange interactions favoring uniform magnetization, magnetocrystalline anisotropy establishing easy axes, and interaction energy with applied magnetic fields [34]. Structural defects—grain boundaries, dislocations, precipitates, and compositional variations—introduce pinning sites, creating local energy barriers that obstruct smooth domain wall propagation. These pinning sites establish a complex, spatially correlated energy landscape characterized by multiple energy minima and saddle points through which domain walls must navigate. The distribution of pinning site strengths reflects material microstructure; materials with high dislocation densities, fine-grained structures, or dense precipitate distributions exhibit stronger pinning and narrower energy valley separations [35]. The effective pinning potential scales with the domain wall width and the pinning site dimensions; domain walls narrower than local pinning features experience weaker interactions, whereas walls comparable to or broader than defect dimensions encounter maximum pinning efficacy [35].

#### 2.1.2. Critical Phenomena and Avalanche Dynamics

The avalanche-like nature of Barkhausen jumps reflects the instability of domain wall configurations trapped in metastable equilibrium positions when subjected to steadily increasing magnetic fields. Below depinning thresholds, walls deform reversibly in response to applied field increases, accumulating elastic energy [34]. Upon exceeding threshold field values, walls escape pinned configurations through cooperative, jerky motion spanning potentially macroscopic spatial extents—the avalanche phenomenon [33]. The stochastic, threshold-dependent nature of these events, coupled with spatial correlations among domain wall segments, produces avalanches of disparate sizes characterized by power-law scaling relationships indicative of critical behavior [6].

#### 2.1.3. Role of Disorder and Dipolar Interactions

Quenched disorder—spatially fixed, time-independent random variations in pinning strength—fundamentally shapes Barkhausen avalanche statistics by establishing competing local energy minima and creating complex depinning dynamics [20]. Dipolar interactions provide long-range magnetic forces coupling distant domain regions, extending effective interaction ranges beyond nearest-neighbor scales characteristic of exchange coupling [36]. The interplay between disorder-induced randomness and long-range dipolar coherence determines which universality class characterizes a specific material: strong dipolar effects promote mean-field behavior, while weakened dipolar coupling (thin films, high-anisotropy materials) drives crossovers toward short-range critical exponents [37]. This fundamental competition explains material-to-material variations in observed scaling exponents and underpins the physical basis for using Barkhausen noise measurements as sensitive magnetic sensors responding to microstructural and stress-induced modifications of disorder and anisotropy landscapes.

### 2.2. Domain Wall Dynamics and Pinning Mechanisms

Building on the critical phenomena discussed in Section 2.1, domain wall dynamics exhibit characteristic temporal profiles—defined as the pulse duration (full width at half maximum of individual Barkhausen voltage pulses), rise time (10–90% amplitude transition), and decay time (time from peak to baseline)—along with amplitude distributions of magnetic Barkhausen noise pulses. These temporal characteristics reflect domain wall avalanche kinetics and pinning site distributions, establishing a critical nexus between microscopic defect configurations and macroscopic magnetic properties detectable through non-destructive sensing methodologies [9]. Understanding these dynamics requires integrating stochastic differential equation frameworks with comprehensive classification of pinning mechanisms operating across diverse microstructural scales, addressing key challenges in sensor development for contemporary magnetic material characterization.

**Figure 2:**
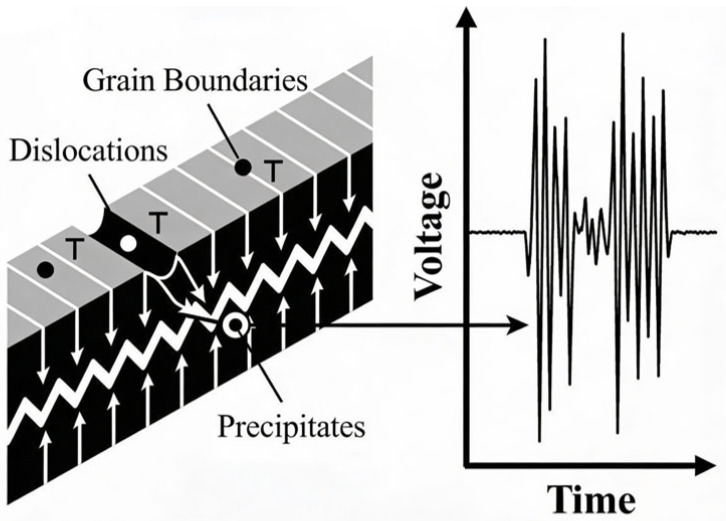
A schematic representation of the Barkhausen noise generation mechanism. Magnetic domain walls moving through a ferromagnetic microstructure interact with pinning sites such as grain boundaries, dislocations, and non-magnetic precipitates. The domain walls remain pinned until the applied magnetic field provides sufficient energy to overcome the local potential barrier (**left**). The sudden, discontinuous depinning of domain walls results in abrupt changes in magnetization, inducing discrete voltage pulses (Barkhausen noise) in a sensing coil. The amplitude and temporal clustering of these pulses directly correlate with the strength and distribution of the microstructural pinning features (**right**).

The fundamental mechanism connecting microstructural features to observed magnetic signals is illustrated in Figure 2. As domain walls traverse the ferromagnetic lattice under an applied field, their motion is impeded by local energy barriers created by crystallographic defects. Figure 2 (left) schematically depicts this interaction, where a domain wall is shown pinned by key microstructural features: grain boundaries, which act as planar pinning sites; dislocations, which introduce localized stress fields; and non-magnetic precipitates, which create voids in the magnetic continuum. The stochastic release of the domain wall from these pinning sites results in discontinuous jumps in magnetization. These abrupt changes induce transient voltage pulses in the pickup coil, as shown in Figure 2 (right). The amplitude and duration of these pulses—collectively forming the Barkhausen noise signal—are thus direct signatures of the specific pinning landscape, allowing for the differentiation between microstructural states such as grain refinement or precipitation hardening.

#### 2.2.1. Stochastic Differential Equations for Domain Wall Motion

Domain wall motion is fundamentally described through the Landau–Lifshitz–Gilbert (LLG) equation, which incorporates competing torques arising from applied and internal magnetic fields, exchange interactions, anisotropy, and dissipative damping [38]. For one-dimensional domain wall models, the equation of motion reduces to a Langevin equation governing the center-of-mass coordinate:

(1) $$X_{0}(t):\;\frac{dX_{0}}{dt}=\frac{\gamma \lambda }{1+\alpha^{2}}[H_{eff}(X_{0},t)-U^{\prime }(X_{0})/M_{s}],$$

where γ denotes the gyromagnetic ratio, λ represents domain wall width, α is the Gilbert damping parameter, and U(X_0_) encompasses the effective pinning potential landscape [38]. Thermal fluctuations introduce stochastic forcing through Langevin noise terms, producing Fokker–Planck equations characterizing probability distributions of domain wall configurations under thermal activation [39]. The combined deterministic driving and stochastic thermal noise regimes produce distinct dynamical behaviors: at low applied fields, creep-dominated motion exhibits exponential field dependence reflecting thermal barrier crossing; at intermediate fields, thermally assisted flux flow emerges; at high fields, viscous depinning flow dominates with velocity saturating due to spin-wave limitations [34,38].

#### 2.2.2. Classification of Pinning Mechanisms

Pinning mechanisms subdivide into three fundamental categories reflecting distinct physical origins:

- Structural pinning arises from crystallographic discontinuities including grain boundaries and phase boundaries. Grain boundaries represent extended defects creating strong pinning sites through multiple mechanisms: localized compositional variations modify exchange interactions, lattice mismatch generates elastic strain fields, and reduced coordination numbers at boundaries alter magnetic anisotropy [40]. The pinning strength of grain boundaries exceeds point defects by orders of magnitude, with coercivity contributions often dominated by grain boundary interactions in polycrystalline materials [41]. Phase boundaries between distinct magnetic or non-magnetic phases similarly create energy barriers; domain walls preferentially align parallel to extended phase boundaries, maximizing pinning through long-range interactions.
- Compositional pinning stems from atomic ordering variations including precipitate phases and compositional segregation. Magnetic precipitates—particles with ferromagnetic ordering differing from the matrix—exert dipolar pinning forces that are maximized when precipitate dimensions approximate domain wall width [42]. Non-magnetic precipitates alternatively pin through elastic deformation of the ferromagnetic matrix surrounding inclusions, creating effective potential barriers [43]. The pinning efficacy of compositional features scales with the volume fraction, size distribution, and spatial arrangement of precipitated phases [44].
- Stress-related pinning reflects magnetoelastic coupling, wherein mechanical stresses modify magnetic anisotropy landscapes through strain-induced energy contributions. Dislocations, representing line defects characterized by Burgers vectors, establish spatially localized strain fields that interact strongly with migrating domain walls [35]. The interaction between dislocations and domain walls exhibits anisotropy depending on Burgers vector orientation and wall type; optimally oriented dislocation arrays produce pinning forces approaching nN scales [45]. Residual stresses—macroscopic stress distributions persisting after plastic deformation or thermal processing—create distributed anisotropy variations that collectively enhance pinning strength [46].

#### 2.2.3. Grain Boundary Interactions and Pinning Strength

Grain boundaries function as the dominant pinning sites in polycrystalline ferromagnets, with pinning strength scales substantially exceeding intragranular defects [47]. High-resolution scanning probe microscopy directly images domain wall pinning at grain boundaries, revealing “sharp” walls precisely coinciding with boundary locations and exhibiting widths determined by electromagnetic tip convolution (~20 nm) rather than intrinsic wall width [47]. The gradient of local coercivity from grain boundary-pinned regions toward grain interiors reflects elastic interactions extending into the grain bulk; domain walls experience gradually decreasing potential barriers away from grain boundaries [47]. Polycrystalline materials with fine-grained structures consequently exhibit enhanced coercivity and reduced permeability through increased grain boundary density, establishing microstructurally sensitive Barkhausen noise parameters responsive to grain refinement processes.

#### 2.2.4. Dislocation–Domain Wall Coupling

Dislocations couple to domain walls through magnetoelastic interactions producing localized anisotropy variations around dislocation cores. Micromagnetic simulations reveal that dislocation pinning strength depends critically on dislocation orientation and Burgers vector relative to domain wall propagation direction [35]. For certain orientations, dislocations create attractive potentials drawing domain walls toward cores; alternative orientations establish repulsive barriers [35]. The pinning of vortex domain walls by artificial dislocation networks demonstrates the manipulability of domain wall dynamics through designed dislocation distributions, enabling engineering of magnetic hardness [45]. Dislocation density, quantifying areal dislocation line length, directly correlates with Barkhausen noise amplitude and coercivity; increased dislocation densities from mechanical deformation or heat treatment produce proportionally stronger pinning and enhanced noise generation [45].

#### 2.2.5. Precipitate Interactions: Magnetic Versus Non-Magnetic

Magnetic precipitates—ferromagnetic or ferrimagnetic phases embedded within softer ferromagnetic matrices—exert strong dipolar pinning through long-range stray fields. Coherent precipitates with small lattice misfits produce weaker pinning than incoherent inclusions, generating significant elastic strain [42]. The interaction volume scales with precipitate size relative to domain wall width; maximum pinning occurs when precipitate dimensions approach wall width, such that walls cannot bypass pinning sites through easy path distortion [44].

Non-magnetic precipitates create pinning through distinct mechanisms: elastic deformation of the surrounding ferromagnetic matrix produces local anisotropy variations, while compositional gradients at particle–matrix interfaces modify exchange interactions [43]. The pinning strength of non-magnetic inclusions depends on inclusion size, hardness, and spatial density.

Enhanced pinning by high precipitate volume fractions suppresses overall MBN RMS amplitudes despite the reviewers’ intuitive expectation of larger individual jumps. Stronger pinning increases depinning force thresholds, fragmenting collective domain wall motion into numerous smaller, more frequent avalanches rather than fewer large events. While individual pulse amplitudes may increase modestly due to higher depinning energies, the dramatic reduction in maximum avalanche size dominates the RMS calculation, as RMS scales with the second moment of the avalanche size distribution ⟨S^2^⟩^1/2^. Materials with dense precipitate networks exhibit power-law avalanche distributions truncated at much smaller characteristic sizes S_max_, producing net MBN suppression consistent with experimental observations in precipitation-hardened alloys [43,44,48].

This counterintuitive behavior—stronger pinning yielding weaker overall noise—underpins MBN’s sensitivity to microstructural hardening states.

#### 2.2.6. Collective Pinning Effects

Collective pinning—the cooperative interaction of domain walls with distributed disorder—substantially enhances pinning efficacy beyond single-site contributions. Larkin collective pinning theory establishes that domain walls effectively “feel” disorder correlations over length scales termed Larkin lengths, defined through balance between elastic wall stiffness and disorder strength [49]. The collective pinning length depends on domain wall width, saturation magnetization, pinning site density, and strength characteristics; narrower walls or stronger disorder reduce Larkin lengths [49]. Scaling analysis reveals creep velocity following power-law dependencies with characteristic exponents reflecting collective pinning universality [50].

#### 2.2.7. Temperature and Stress Dependencies

Temperature effects on domain wall dynamics operate through multiple mechanisms: thermal activation of depinning transitions, temperature-dependent saturation magnetization evolution, and modifications of damping parameters [51]. Creep-dominated dynamics exhibit logarithmic temperature dependence of pinning barrier heights; reducing temperature increases effective pinning strength through suppressed thermal assistance to barrier crossing. Stress dependencies reflect magnetoelastic coupling; applied or residual stresses modify the magnetic anisotropy landscape, increasing or decreasing pinning potential depending on stress orientation relative to easy magnetic axes [52]. Quantitative relationships between stress-induced pinning changes and Barkhausen noise parameters enable non-destructive stress evaluation, a key application for magnetic sensor technologies in structural monitoring and quality assurance.

### 2.3. Statistical Models and Scaling Laws

The statistical characterization of magnetic Barkhausen noise represents a sophisticated intersection of critical phenomena, random processes, and sensor physics, providing fundamental insights into material microstructure and magnetic dynamics amenable to quantitative non-destructive evaluation [18,53]. Understanding these statistical frameworks and associated scaling laws proves essential for developing robust sensor technologies capable of extracting quantitative material properties from noisy, complex magnetic signals—a central objective for sensor applications aligned with contemporary material characterization demands.

#### 2.3.1. Avalanche Models and Critical Point Behavior

Avalanche models describe the discrete, impulsive magnetization events arising from domain wall depinning transitions in disordered ferromagnetic systems [53]. The fundamental insight establishing connections between Barkhausen noise and critical phenomena emerges through recognition that ferromagnetic domain walls exhibit critical point behavior characteristic of disorder-driven phase transitions [18]. At the critical disorder strength—or equivalently, at the depinning transition field—systems display scale-invariant avalanche statistics wherein avalanche sizes span multiple orders of magnitude without characteristic scales [53]. The theoretical framework, established through the random field Ising model (RFIM) formalism introduced by Sethna, Dahmen, and collaborators, demonstrates that magnetic systems at critical depinning transitions generate avalanches distributed identically to those in completely different physical systems—earthquakes, plastic deformation, and neuroscience avalanches—establishing universality [20,53]. This universality emerges from fundamental symmetries and dimensionality rather than microscopic details, rendering Barkhausen noise a paradigmatic experimental system for investigating critical phenomena.

#### 2.3.2. Probability Distributions for Avalanche Size and Duration

Avalanche size distributions follow power-law scaling in the critical regime $P(S)∼S^{-\tau }$, where S denotes the total magnetization change (voltage-integrated magnitude) and τ represents the avalanche size exponent [18]. Similarly, avalanche duration distributions exhibit $P(T)∼T^{-\alpha }$, where T represents the temporal extent and α denotes the duration exponent [6]. These power-law distributions extend over multiple decades before exponential cutoffs determined by system size limitations and demagnetizing field effects [18]. Empirical measurements reveal that polycrystalline ferromagnets typically display τ ≈ 1.50 ± 0.05, while amorphous materials show τ ≈ 1.27 ± 0.03, reflecting different universality classes [18]. Duration exponents span α ≈ 1.5–2.0 depending on system dimensionality and interaction range [54]. Critically, these exponents are not independent; they obey the crackling noise scaling relation $\gamma \approx \frac{\tau -1}{\alpha -1}$, where γ characterizes the average avalanche size scaling with duration through $\langle S\rangle_{T}∼T^{\gamma }$ [53]. This exponent relationship provides a quantitative test for proximity to criticality, applicable across diverse experimental systems.

#### 2.3.3. Scaling Exponents and Universality Classes

Scaling exponents characterizing avalanche distributions depend fundamentally on system dimensionality and the range of interactions governing domain wall dynamics [54]. Universality classes represent groups of systems with identical macroscopic properties despite disparate microscopic details, characterized by specific exponent sets [18]. In three-dimensional systems with long-range dipolar interactions (characteristic of polycrystalline ferromagnets), mean-field exponents emerge, τ = 3/2 and α = 2, with the exponent relation γ = 2 [2]. Two-dimensional systems or materials with short-range interactions exhibit distinct exponents, τ ≈ 1.27, α ≈ 1.5, and γ ≈ 1.77, reflecting alternative universality classes [37]. Thin ferromagnetic films exhibit dimensional crossovers wherein reducing thickness transitions from three- to two-dimensional behavior, with corresponding changes in observed scaling exponents [54]. Temperature variations near magnetic phase transitions induce universality class crossovers reflecting competing interaction mechanisms [54].

#### 2.3.4. Mean-Field Approximations

Mean-field theory provides analytical predictions for critical exponents through approximations replacing spatially distributed systems with representative average quantities [20]. For the RFIM in dimensions d ≥ 3, mean-field predictions yield τ = 3/2 and α = 2, independent of driving rate [2]. Mean-field theory further predicts that avalanche temporal profiles—average magnetization versus normalized time t/T for avalanches of duration T—collapse onto universal functions: inverted parabolic shapes ∝ (1 − (t/T)^2^) characterize mean-field systems [53]. The power spectrum of Barkhausen signals within avalanches scales as

(2) $$S(f)∼f^{-1/(\sigma \nu z)},$$

where *σ*, *ν*, and *z* denote the exponent relating size to duration [55]. Theoretical models demonstrate that mean-field behavior emerges when long-range dipolar interactions dominate, as realized in bulk ferromagnets where the dipolar interaction range exceeds system dimensions [2].

#### 2.3.5. Finite-Size Scaling Theory

Finite-size scaling addresses how avalanche statistics evolve with system size L, particularly approaching critical points where correlation lengths diverge [54]. For observables O near criticality, finite-size scaling ansatz predicts the following:

(3) $$O(L,|R-R_{c}|)=L^{x_{O}/\nu }f(|R-R_{c}|L^{1/\nu })$$

where R represents disorder, R_c_ denotes critical disorder, ν is the correlation length exponent, and f denotes a universal scaling function [56]. This formulation enables extrapolation of critical behavior from finite experimental systems to thermodynamic limits through appropriate scaling collapses [56]. For systems above the upper critical dimension (d > 6 for RFIM), an effective length scale $L_{eff}=L^{d/d_{u}}\;$ replaces linear L, where d_u_ represents the upper critical dimension [56].

#### 2.3.6. Dynamic Scaling and Driving Rate Effects

Driving rate effects—modifications of avalanche statistics with field sweep rate—fundamentally characterize domain wall dynamics and depinning transitions [6]. The avalanche size exponent exhibits linear dependence on driving rate, $\tau (v)=\tau_{0}-c\cdot v$, where v denotes driving field rate and c characterizes rate sensitivity [6]. This driving rate dependence reflects competition between thermal activation over pinning barriers (dominant at low rates, producing creep dynamics) and viscous depinning flow (dominant at high rates) [47]. Dynamic scaling theory predicts that avalanche distributions, when properly rescaled, collapse onto universal forms independent of driving rate at criticality [6]. Frequency-dependent measurements reveal that excitation frequency directly modulates observed exponents through skin-effect-induced depth profiling, providing methodological leverage for depth-sensitive characterization.

#### 2.3.7. Multifractal Analysis of Barkhausen Signals

Multifractal analysis extends beyond traditional power-law scaling to characterize complex temporal correlations within Barkhausen signals through generalized Hurst exponents h(q) computed from detrended fluctuation analysis (DFA) [57]. For monofractal processes—exhibiting uniform scaling across all timescales—h(q) remains independent of moment order q. Multifractal processes display q-dependent Hurst exponents reflecting asymmetric behavior between large and small fluctuations [54]. Research demonstrates that polycrystalline and amorphous ferromagnetic films exhibit dimensional-dependent multifractal properties: three-dimensional films display a multifractal structure at short timescales, transitioning to monofractal (random) behavior at longer scales [54]. Two-dimensional films exhibit monofractal behavior throughout, with Hurst exponent α ≈ 0.80 indicating strong temporal correlations [54]. The multifractal spectrum width correlates with disorder strength and pinning regime characterization, enabling quantitative discrimination between weak-pinning (individual wall motion) and strong-pinning (collective avalanche) regimes [58]. Temporal profile analysis through phase space network methods reveals that avalanche complexity—quantified through higher-order graph structures—correlates with collective dynamics intensity, particularly in hysteresis loop central regions [59].

These interconnected statistical frameworks—from power-law distributions through universality class identification to multifractal temporal characterization—establish comprehensive quantitative approaches for extracting microstructural and stress state information from Barkhausen noise measurements, enabling the development of sophisticated magnetic sensor systems for non-destructive material evaluation supporting contemporary Industry 4.0 manufacturing paradigms.

#### 2.3.8. Recent Modeling Tools and Simulation Frameworks

While phenomenological models like Jiles–Atherton provide macroscopic insights, recent advances focus on spatially resolved simulations of domain dynamics. Stochastic models, such as the Alessandro–Beatrice–Bertotti–Montorsi (ABBM) model and its generalizations, treat domain wall motion as a random walk in a Brownian potential, successfully predicting avalanche statistics and power spectra in soft magnetic materials.

For detailed microstructural analysis, micromagnetic simulation tools such as OOMMF and MuMax3 have become standard. These solvers integrate the Landau–Lifshitz–Gilbert (LLG) equation on nanoscale grids, allowing researchers to directly visualize domain wall pinning at specific defects like precipitates or grain boundaries. Although computationally intensive, they provide a “virtual microscope” for validating theoretical predictions of pinning strength.

At the macroscopic sensor scale, Finite Element Method (FEM) software (e.g., COMSOL Multiphysics version 6.4, ANSYS Maxwell 2025 R1) is increasingly used to model the electromagnetic interaction between the sensor probe and the component geometry. These tools simulate the distribution of the excitation field and eddy currents, enabling the optimization of yoke geometry and coil configurations for complex sample shapes.

## 3. Instrumentation and Measurement Systems

### 3.1. Sensor Design and Configuration

The design and configuration of magnetic Barkhausen noise sensors critically determine measurement sensitivity, spatial resolution, frequency response, and practical applicability across diverse industrial and research environments [24,60]. Contemporary sensor architectures span from simple contact surface configurations through advanced multi-parameter systems incorporating high-entropy alloys and wireless integration, exemplifying the synergistic advancement of sensor physics with material innovation and digital technologies aligned with the *Sensors* journal’s emphasis on sensor system developments.

#### 3.1.1. Contact Surface Sensors and Basic Configuration

Contact surface sensors represent the standard MBN measurement approach, employing direct placement of a sensing coil assembly against the specimen surface to detect magnetic flux variations during magnetization [61]. The fundamental architecture comprises the following: (1) an excitation coil wound around a U-shaped magnetic yoke generating controlled alternating magnetic fields typically operating at 1–1000 Hz excitation frequencies; (2) a pickup coil adjacent to the sample surface detecting induced voltage proportional to magnetization rate changes [61]. The magnetic yoke, typically constructed from soft ferromagnetic materials with low coercivity, guides magnetic flux through the specimen and return path, establishing the magnetization field. The contact pressure and coupling quality between sensor pole pieces and sample surface directly affect signal amplitude and reproducibility; surface roughness and curvature variations produce measurement uncertainty exceeding ±10% [24]. Basic configuration sensors employ air gap designs with planar detection coils directly facing the sample, enabling rapid measurements on production lines but sacrificing sensitivity compared to enhanced designs [60].

#### 3.1.2. Air Coil Sensors: Simplicity and Frequency Response

Air coil sensors—utilizing coils wound in non-magnetic geometries without ferromagnetic cores—demonstrate exceptional frequency response characteristics enabling high-frequency operation [62]. The absence of a core material eliminates frequency-dependent saturation and core loss contributions, maintaining linear frequency response across octave-spanning ranges. Air coil sensitivities, while reduced compared to ferrite core configurations, enable wide-band measurement from kilohertz to megahertz frequencies through appropriate excitation and conditioning electronics [62]. The simplified construction facilitates miniaturization and integration into complex multi-axis sensor arrays; planar spiral coil designs achieve spatial resolution approaching sub-millimeter dimensions through restricted measurement volumes [62]. Air coils demonstrate superior environmental stability, avoiding temperature-dependent core permeability variations affecting ferrite cores [62].

#### 3.1.3. Ferrite Core Sensors: Enhanced Sensitivity Through Flux Concentration

Ferrite core sensors employ ferromagnetic cores exhibiting high permeability and low coercivity to concentrate magnetic flux through pickup coils, enhancing voltage signal generation by 10–50× compared to air core equivalents [62]. Typical ferrite core materials include manganese–zinc (MnZn) and nickel–zinc (NiZn) compositions, each exhibiting distinct frequency–permeability characteristics: MnZn ferrites maximize permeability at frequencies < 1 MHz but suffer elevated core losses at high frequencies; NiZn ferrites exhibit flat permeability extending to higher frequencies with reduced low-frequency permeability [62]. The permeability enhancement factor η = μ_r/μ_0 ranges from 200 to 2000 depending on core material and geometry, directly amplifying detected signals proportionally. However, core saturation at high excitation field amplitudes and temperature-dependent permeability variations limit operational ranges and introduce nonlinearities [62]. Advanced core designs incorporating distributed air gaps and composite structures modulate permeability to optimize sensitivity–bandwidth trade-offs [62].

#### 3.1.4. High-Spatial-Resolution Sensors: Sub-Millimeter Detection

To achieve sub-millimeter lateral spatial resolution through restricted measurement volumes established by small-diameter, tightly wound pickup coils [60], measurement volumes scale approximately as $V_{eff}∼(d_{c}{)}^{3}$, where d_c_ denotes coil diameter. Reducing coil diameter from 5 mm to 1 mm decreases the effective volume 125-fold, dramatically enhancing spatial resolution [60]. However, sensitivity reduction with scaling follows $S\propto N\times A\propto d_{c}^{2}$, necessitating increased coil turn counts N and opposing spatial resolution improvements [60]. Practical high-resolution systems employ (1) compact U-shaped yokes with gaps < 300 μm; (2) multi-turn pickup coils (50–100 turns) concentrating around gap regions; and (3) shielded cable configurations minimizing environmental pickup noise [60]. Applications include mapping residual stress distributions in case-hardened components and detecting localized microstructural defects in welded joints, enabling defect localization to ±0.5 mm precision [63].

#### 3.1.5. Solenoid-Type Non-Contact Sensors

Non-contact solenoid sensors position magnetizing and detecting coils coaxially around specimen surfaces at standoff distances of 1–10 mm, eliminating contact pressure and surface condition dependencies [64]. The solenoid configuration establishes approximately uniform axial magnetic fields through the specimen cross-section, enabling measurements on irregular geometries and curved surfaces inaccessible to contact sensors [64]. However, magnetic field non-uniformity increases with standoff distance, reducing effective spatial resolution and introducing standoff-dependent signal variations requiring calibration correction [64]. Non-contact operation substantially improves measurement repeatability and eliminates pressure-induced contact effects, critical for quality control automation [64].

#### 3.1.6. Advanced Core Materials: High-Entropy Alloys

High-entropy alloy (HEA) cores represent emerging materials incorporating FeCoNi and related multicomponent systems, offering engineered magnetic properties surpassing those of conventional ferrites [65]. Compositions such as FeCoNi(AlMn)_0.25_ HEAs display soft ferromagnetic characteristics with saturation magnetization M_s_ ≈ 100 Am^2^/kg and coercivity H_c_ < 2 mT, competing with or exceeding standard ferrite performance [65]. The configurational entropy stabilization in HEAs suppresses secondary phase precipitation, enabling compositional tuning to achieve optimal permeability–bandwidth characteristics across operational frequency ranges [65]. High-entropy cores exhibit superior temperature stability compared to conventional ferrites, with permeability variations < 5% across 0–100 °C ranges, substantially enhancing measurement reproducibility in field environments [65].

#### 3.1.7. Multi-Parameter Sensor Systems

Multi-parameter sensor systems simultaneously acquire MBN alongside complementary magnetic signals including harmonic content, incremental permeability, and magnetic impedance variations, enabling discriminatory material characterization [64]. Typical systems employ (1) fundamental frequency excitation (5–100 Hz) for traditional MBN acquisition; (2) harmonic excitation at 100–500 kHz enabling eddy current and permeability-based characterization; and (3) dual-frequency excitation combining low-frequency MBN with high-frequency magnetic impedance measurement [64]. Information fusion from multiple measurement modes substantially improves discrimination between competing microstructural effects—grain size, dislocation density, and residual stress—previously difficult to decouple from single-parameter measurements [64].

#### 3.1.8. Sensor Calibration and Standardization

Standardization challenges remain significant obstacles to industrial MBN adoption, with the absence of internationally agreed calibration protocols and measurement procedures compromising interlaboratory comparability [24]. Laboratory round-robin studies comparing equipment from different manufacturers reveal measurement variations exceeding ±15% despite nominally identical specimen conditioning, including sensor design, excitation amplitude, filtering characteristics, and software algorithms [24]. Recommended calibration practices include (1) reference specimen sets spanning target property ranges established through rigorous characterization; (2) monthly sensor calibration verification against reference materials; (3) environmental control maintaining temperature within ±2 °C; and (4) standardized measurement repetition protocols (typically 5–10 measurements) with documented uncertainty quantification [24].

#### 3.1.9. Environmental Considerations for Field Applications

Field deployment robustness requires environmental stability addressing temperature variations, electromagnetic interference, humidity exposure, and mechanical vibration [66]. Temperature coefficients of MBN parameters typically range from −0.2% to +0.8%/°C necessitating temperature-compensated algorithms for ±5 °C ambient variations encountered in industrial settings [66]. Shielded cables with ferromagnetic sleeves attenuate external electromagnetic interference; ferrite toroidal clamps provide additional 20–40 dB attenuation at problematic frequency bands [66]. Conformal coatings and sealed connectors protect electronics from moisture ingress; IP67 enclosures enable temporary water jet exposure without component damage [66].

#### 3.1.10. Miniaturization and Wireless Sensor Systems

Miniaturized wireless systems represent emerging frontiers combining compact magnetometer architectures—sub-2 cm^3^ volumes—with low-power wireless communication enabling Internet-of-Things (IoT) integration for distributed structural health monitoring [26]. Passive Barkhausen noise sensors incorporating energy harvesting from excitation field oscillations achieve battery-free operation, extending deployment lifetimes to decades without maintenance [26]. Wireless data transmission via Bluetooth Low Energy (BLE) or LoRaWAN protocols accommodates real-time remote monitoring with <1% data loss across industrial facility distances, supporting autonomous quality feedback systems and predictive maintenance algorithms [26]. Cloud integration enables machine learning-based anomaly detection through historical trend analysis and multivariate pattern recognition, substantially improving diagnostic accuracy compared to single-point measurements [26].

### 3.2. Signal Processing and Analysis Methods

The signal processing and analysis of magnetic Barkhausen noise represents a critical nexus between raw sensor measurements and quantitative material property evaluation, requiring integrated multi-domain approaches spanning traditional statistical analysis through contemporary artificial intelligence methodologies [4,24]. Modern sensor systems incorporate sophisticated digital processing architectures enabling real-time feature extraction and automated decision-making, essential for industrial implementation aligned with Industry 4.0 paradigms’ emphasis on integrated sensor–data systems.

#### 3.2.1. Time-Domain Analysis Fundamentals

Time-domain analysis directly examines the raw Barkhausen noise waveform acquired from sensor coils, extracting parameters reflecting magnetization dynamics through statistical characterization [24]. The root-mean-square (RMS) voltage, defined as $\mathrm{RMS}=\sqrt{\frac{1}{N}\sum_{i=1}^{N}V_{i}^{2}}$, represents the fundamental parameter quantifying overall signal magnitude and exhibits strong correlations with microstructural features and mechanical properties [24]. Pulse counting methods enumerate individual Barkhausen events exceeding defined threshold voltages, providing avalanche size statistics sensitive to pinning strength distributions; event counts typically range from thousands to millions depending on material hardness and excitation parameters [3].

Envelope analysis—extracting the outer boundary of Barkhausen signal oscillations through analytic signal processing—reveals the temporal profile of magnetization activity synchronized with applied field variations [67]. The envelope’s peak amplitude, position relative to the coercive field, width characteristics, and integrated area collectively characterize material state, with envelope parameters demonstrating superior sensitivity to residual stress compared to instantaneous RMS values in certain applications [67]. Peak amplitude detection identifies maximum voltage values within individual Barkhausen pulses, establishing statistical distributions enabling avalanche magnitude quantification; peak distributions consistently exhibit a power-law nature near criticality [18]. These temporal characteristics (pulse duration, rise time, decay time) provide complementary information to amplitude-based parameters, with shorter durations indicating stronger pinning landscapes.

#### 3.2.2. Frequency-Domain Techniques

Fast Fourier transform (FFT) analysis decomposes Barkhausen signals into frequency-domain representations, revealing spectral energy distributions across measurement bandwidth (typically 1–50 kHz for conventional systems) [68]. The power spectral density (PSD) $S(f)=|X(f){|}^{2}/(\Delta f)$, where *X*(*f*) denotes FFT amplitude and Δf represents frequency resolution, characterizes the distribution of magnetic energy across frequency bands [68]. Empirical investigations reveal that low-frequency PSD components (1–10 kHz) relate to bulk magnetic response and dislocation effects, while mid-frequency bands (10–25 kHz) show optimal sensitivity to stress and hardness variations, and high-frequency components (25–50 kHz) carry a predominantly noise-like nature with reduced material sensitivity [68]. Spectral centroid calculation—the frequency-weighted center-of-mass of the PSD—provides a single parameter quantifying spectral distribution shifts accompanying material property changes; the spectral centroid typically shifts by 2–5 kHz between fully annealed and hardened steels [68].

#### 3.2.3. Time–Frequency Analysis: STFT and Wavelets

Short-time Fourier transform (STFT) analysis applies Fourier transformation to consecutive windowed signal segments, generating spectrograms representing signal energy distributions across time–frequency planes: $X_{STFT}(t,f)=\int_{-\infty }^{\infty }x(\tau )w(\tau -t)e^{-j2\pi f\tau }d\tau$. Here, *w*(*τ*) denotes the window function [69]. The STFT overcomes time-domain and frequency-domain limitations through simultaneous temporal and spectral resolution, revealing how spectral content evolves throughout magnetization cycles [69]. Window selection—including Hamming, Blackman, and Kaiser windows—critically affects resolution trade-offs; larger windows enhance frequency resolution while reducing temporal resolution, necessitating empirical optimization balancing application requirements [69]. Spectrogram analysis reveals that maximum Barkhausen activity concentrates near magnetization reversal points (H ≈ H_c_), with spectral characteristics broadening under applied tensile stress and narrowing under compressive stress [69].

Wavelet analysis employs basis functions (wavelets) through continuous or discrete decomposition, enabling multi-scale feature extraction from non-stationary Barkhausen signals [27]. Continuous wavelet transforms (CWTs) using Morlet or Mexican hat wavelets reveal temporal localization of high-frequency energy bursts associated with individual avalanche events, with wavelet coefficients at short timescales (high frequencies) correlating directly with avalanche sizes [27]. Discrete wavelet transforms (DWTs) efficiently decompose signals into frequency bands through dyadic filter banks; approximate (cA) and detail (cD) coefficients at multiple decomposition levels provide multi-resolution characterization, enabling discrimination between near-surface and bulk-associated phenomena through depth-selective analysis [27].

#### 3.2.4. Machine Learning Approaches

Artificial neural networks (ANNs)—fully connected architectures with multiple hidden layers—learn complex, nonlinear relationships between Barkhausen features and material properties without explicit physical models [70]. Back-propagation training algorithms adjusting synaptic weights through gradient descent optimization achieve excellent performance on calibration datasets; however, limited generalization to new materials or stress–temperature regimes represents significant challenges [70]. Typical ANN architectures for Barkhausen analysis employ 50–100 extracted features as input nodes, 1–3 hidden layers with 20–100 neurons each, and single or multiple output nodes for property prediction; optimal network topologies vary considerably with application specifics [70].

Support vector machines (SVMs)—kernel-based methods defining optimal separating hyperplanes in high-dimensional feature spaces—demonstrate robust classification performance for binary material state discrimination (healthy/defective, stress regimes) [71]. SVM advantages include theoretical guarantees regarding generalization capability and reduced sensitivity to training dataset size compared to ANNs; however, multiclass problems (multiple stress levels, microstructural states) require one-versus-all or directed acyclic graph implementations complicating practical deployment [71].

Deep learning algorithms—particularly convolutional neural networks (CNNs)—excel at automated feature discovery from raw Barkhausen data or time–frequency spectrograms without manual feature engineering [4,22]. CNN architectures combining convolutional layers extracting spatial–spectral patterns with max-pooling layers reducing dimensionality achieve test accuracies of 91–99% for stress prediction, microstructure classification, and hardness estimation [22]. Long short-term memory (LSTM) recurrent networks capture temporal dependencies within sequential Barkhausen pulse trains, particularly valuable for fatigue damage monitoring or time-evolving stress states [22]. Hybrid architectures combining multiple network types (CNN-LSTM fusion) incorporate both spectral and temporal information, demonstrating superior performance on complex material characterization tasks [22].

#### 3.2.5. Feature Extraction and Digital Filtering

Feature extraction procedures systematically compute distinctive parameters from raw signals, reducing data dimensionality from millions of temporal samples to dozens of engineered metrics: classic examples include RMS amplitude, peak position, spectral centroid, wavelet coefficients at specific scales, and entropy measures quantifying signal complexity [72]. Digital filtering—applying frequency-selective processing before analysis—attenuates environmental noise and removes dc components; typical bandpass filters (2–70 kHz) concentrate analysis within Barkhausen frequency bands while rejecting 50/60 Hz power-line interference and low-frequency electromagnetic pickup [24].

#### 3.2.6. Real-Time Processing Capabilities

Real-time processing requirements for industrial implementations necessitate sub-millisecond latency from measurement acquisition through property determination, achievable through firmware implementation of simplified algorithms on field-programmable gate arrays (FPGAs) or embedded processors [3]. Modern instruments acquire Barkhausen signals at 500 kHz–1 MHz sampling rates, generating gigabit-scale data flows; on-sensor preprocessing computes envelope parameters and spectral characteristics in real time, reducing transmitted data volumes to kilobits and enabling wireless deployment [26]. Machine learning inference accelerators (neuromorphic chips, tensor processing units) enable sub-10 ms prediction latency for CNN-based material property assessment, supporting autonomous quality control and predictive maintenance applications in manufacturing environments [22].

### 3.3. Measurement Parameters and Calibration

The measurement parameters and calibration methodologies of magnetic Barkhausen noise systems represent critical determinants of quantitative accuracy, reproducibility, and applicability across diverse industrial and research contexts [24,73]. Comprehensive understanding of excitation characteristics, sensor coupling requirements, signal conditioning architectures, and standardized calibration procedures enables the development of robust measurement frameworks supporting deployment within advanced manufacturing and structural health monitoring systems, with an emphasis on sensor system integration.

#### 3.3.1. Excitation Parameters: Frequency, Amplitude, and Waveform

Magnetizing frequency fundamentally determines the temporal dynamics of domain wall motion and the frequency content of detected Barkhausen signals [74]. Typical industrial systems employ frequencies ranging from 5 to 200 Hz, with lower frequencies (5–50 Hz) enabling slower domain wall motion amenable to stress-related sensitivity, while higher frequencies (50–200 Hz) accelerate magnetization rates and enhance spectral power density [74]. The excitation frequency directly modulates penetration depth through the electromagnetic skin effect; the characteristic penetration depth *δ* is calculated as follows:

(4) $$\delta =\sqrt{\frac{2}{\mu_{0}\mu_{r}\sigma \omega }}$$

where *μ*_0_ denotes the permeability of free space, μ_r represents relative permeability, σ signifies electrical conductivity, and *ω* = 2π*f* denotes angular frequency [75]. Reduced frequencies penetrate deeper; 100 Hz magnetizing fields penetrate ~2 mm in steels, whereas 125 kHz analysis frequency restricts sensitivity to ~0.1 mm surfaces, enabling depth-selective characterization through multi-frequency excitation strategies [75].

Excitation amplitudes typically range from 1 to 10 V peak to peak (Vpp); however, it is critical to note that the drive voltage alone provides no information about the actual magnetic field strength induced in the material. The resulting tangential magnetic field (H) depends entirely on the specific excitation system parameters, including the number of coil turns, the magnetic permeability of the yoke, and the effective reluctance of the magnetic circuit (which includes the air gap and sample geometry). Therefore, reporting only the excitation voltage is insufficient for reproducibility. Instead, the tangential magnetic field at the sample surface—typically ranging from 0.5 to 15 kA/m for standard industrial sensors—should always be measured (e.g., using a Hall probe) and reported alongside the drive voltage to ensure comparable magnetization conditions across different experimental setups [24]. Sublinear amplitude dependencies characterize the lower amplitude regime where domain wall motion remains restricted to small reversible excursions; increasing the amplitude to moderate ranges (3–6 Vpp) produces approximately linear MBN amplitude enhancements, reflecting increased irreversible magnetization contributions [74]. Beyond 6 Vpp, saturation effects reduce sensitivity gains through complete magnetization reversal independent of further amplitude increments [74]. Waveform selection—sinusoidal versus triangular excitation—influences spectral distributions and temporal pulse characteristics; sinusoidal waveforms produce symmetric bipolar magnetization cycles, while triangular waveforms generate asymmetric reversals with modified avalanche size distributions, reflecting altered domain wall dynamics [74].

#### 3.3.2. Sensor Coupling and Positioning Requirements

Sensor–specimen contact quality critically determines measurement reproducibility and quantitative accuracy [24]. The contact quality between the sensor pole pieces and the sample surface is critical for signal reproducibility. While earlier studies often reported contact force in Newtons (typically 5–20 N), this metric is ambiguous without specifying the contact area. To ensure consistent magnetic coupling and minimize air gap variations, it is more informative to standardize the contact pressure. Optimal reproducibility is generally achieved at contact pressures ranging from 0.5 to 2.0 MPa, depending on the pole piece surface area (typically 10–50 mm^2^). This pressure range ensures stable magnetic contact without inducing significant localized elastic strain that could artifactually alter the Barkhausen signal [24]. Surface cleanliness proves essential; ferromagnetic particles, grinding dust, or oxide films establish parasitic coupling paths distorting normal electromagnetic interactions; cleaning with lint-free cloths followed by light oil application substantially improves repeatability [75]. The geometric positioning of sensor pole pieces relative to grain structure, surface finish direction, and applied stress orientation directly influences measured MBN parameters; deviations from specified orientations exceeding ±5° produce signal variations of 10–20% [24]. Magnetic alignment relative to principal stress axes provides directional stress sensitivity; optimal stress discrimination occurs when the measurement direction aligns perpendicular to the grinding or stress direction, establishing maximum magnetization changes [24].

#### 3.3.3. Signal Conditioning: Amplification, Filtering, and Digitization

Pre-amplification stages boost weak Barkhausen signals—typically in the 10–100 mV range—to levels appropriate for analog-to-digital conversion without introducing prohibitive noise [24]. Instrumentation amplifiers with gains of 100–10,000× achieve noise figures < 2 dB, enabling the recovery of avalanche size distributions spanning three orders of magnitude [24]. Bandpass filtering—typically confining analysis to 1–200 kHz ranges—attenuates 50/60 Hz power-line interference, subsonic mechanical vibrations, and ultrasonic environmental noise while preserving Barkhausen frequency content [24]. High-order elliptic or Chebyshev filters provide steep roll-off characteristics; specified filter order, cutoff frequencies, and ripple specifications must be documented for reproducibility [24].

Analog-to-digital conversion with 12–14 bit resolution enables quantization of signals into 4096–16,384 distinct levels, balancing noise immunity against dynamic range compression [24]. Sampling rates must satisfy Nyquist criteria: analysis frequency spans of up to 200 kHz require sampling ≥ 400 kHz to avoid aliasing; practical systems employ 500 kHz–1 MHz sampling, enabling sub-microsecond temporal resolution necessary for individual avalanche pulse reconstruction [24].

#### 3.3.4. Measurement Duration and Averaging Procedures

Complete magnetization cycles—from coercive field H_c_ through maximum applied field and return to H_c_—require 10–100 milliseconds depending on magnetizing frequency [24]. Typical protocols specify acquisition duration encompassing three to five complete cycles, generating datasets of 5–500 million temporal samples per measurement [24]. Measurement repetition is essential due to the stochastic nature of Barkhausen phenomena. A minimum of 3–10 repetitions establish robust parameter averages with quantifiable uncertainty; industrial quality control typically employs 5–10 repetitions, balancing statistical robustness against production throughput requirements [24]. Averaging procedures—including moving-average filtering and ensemble averaging across repeated measurements—reduce noise while preserving envelope shape and peak characteristics; excessive averaging risks suppressing avalanche signatures [24].

#### 3.3.5. Environmental Control Requirements

Temperature stability represents critical calibration preservation requirements; MBN parameters exhibit temperature coefficients of ±0.2–0.8%/°C [24]. Industrial laboratories maintain measurement environments within ±2 °C through climate control or compensate through calibrated temperature-dependent correction algorithms [24]. Electromagnetic interference (EMI) from nearby power equipment, wireless devices, or electrical machinery corrupts measurements; ferromagnetic cable shielding with grounding to instrument chassis and specimen connection to identical electrical potential attenuate interference by 20–40 dB [75]. Humidity control prevents moisture-induced sensor performance drift; sealed connectors and conformal coatings protect electronics in field environments [24].

#### 3.3.6. Calibration Methodologies

Hardware calibration involves periodic voltage and frequency verification against traceable laboratory standards (typically annually), ensuring that instrument specifications remain within documented tolerances [24]. Standard reference materials (SRMs)—steel specimens with certified hardness (e.g., Vickers hardness 450 ± 10 HV), residual stress (e.g., −50 ± 20 MPa), or microstructural parameters—establish quantitative calibration relationships [24]. Typical calibration protocols employ 5–10 reference specimens spanning property ranges of interest, with measured MBN parameters plotted against certified properties to establish regression models predicting unknown specimen characteristics [73]. Stress calibration employs mechanically stressed specimens loaded in uniaxial tension or compression (0–500 MPa typically) while measuring MBN amplitude dependence on applied stress, establishing sensitivity coefficients of ±1–5 mV per 100 MPa depending on the material [73]. Hardness calibration utilizes hardened/tempered steel samples with Vickers hardness spanning 300–700 HV, with MBN parameters exhibiting logarithmic or power-law relationships with hardness over appropriate ranges [73]. Microstructural calibration references grain-size-characterized specimens—established through quantitative metallography or electron microscopy—with MBN envelope parameters showing inverse power scaling with grain diameter [73].

#### 3.3.7. Traceability and Uncertainty Analysis

Metrological traceability requires unbroken calibration chains from measured specimen properties to national/international standards (NIST, NPL, PTB) through documented comparison procedures with quantified uncertainties at each step [24]. Uncertainty analysis quantifies combined contributions from hardware calibration uncertainty (typically ±2–5%), reference material certified value uncertainties (typically ±5–10%), measurement repeatability (typically ±5–15%), and environmental fluctuation effects (typically ±3–5%), yielding combined uncertainties of ±8–20% for property predictions [24].

#### 3.3.8. Quality Control Procedures

Gauge R&R (Repeatability and Reproducibility) testing statistically quantifies measurement system capability through repeated measurements by different operators using different sensors on reference specimens, establishing whether measurement variation derives from operator technique, equipment degradation, or inherent measurement limitations [24]. Monthly calibration verification against working reference specimens detects sensor drift or instrument degradation, triggering recalibration if deviations exceed ±5% [24].

### 3.4. Advanced Detection Systems

The advanced detection systems for magnetic Barkhausen noise represent cutting-edge implementations integrating sophisticated sensor architectures, multi-parameter measurement frameworks, and intelligent processing algorithms to address demanding applications across aerospace, automotive, and structural monitoring domains [27,76,77,78]. Contemporary technological advances span from grain-scale spatial resolution sensors through distributed wireless networks incorporating artificial intelligence, exemplifying the convergence of material characterization physics with modern sensor technology and digital systems.

#### 3.4.1. High-Spatial-Resolution MBN Sensors: Grain-Scale Measurements

Micromagnetic high-resolution MBN sensors achieve unprecedented spatial resolution approaching sub-millimeter dimensions through advanced probe design incorporating shielded receiver architectures and precision U-shaped magnetic yokes [63]. The fundamental design approach employs thin-film ferromagnetic shielding layers surrounding receiver coils, restricting the effective measurement volume to localized regions typically 2–5 mm in lateral extent, enabling grain-scale measurements of MBN signal distributions across individual crystallographic grains and grain boundaries [27]. Time–response histogram analysis reveals distinctly different domain wall motion patterns within grains versus at grain boundaries: intragranular signals display rapid, continuous behavior reflecting uniform domain dynamics, whereas grain boundary regions exhibit complex, multi-phase temporal profiles reflecting pinning deceleration sequences [27]. Resolution enhancements to ~1 mm dimensions require specialized coil designs concentrating flux into restricted gaps. Rather than relying on a fixed turn count, these coils are optimized to balance inductance and resonant frequency, ensuring high sensitivity within the material-specific Barkhausen frequency band while minimizing thermal noise. This design sacrifices absolute voltage amplitude for dramatically improved spatial discrimination [27].

#### 3.4.2. Multi-Element Sensor Arrays: Linear and 2D Configurations

Linear sensor arrays—comprising 8–32 individually controlled sensing elements along a measurement line—enable rapid scanning of component surfaces without mechanical repositioning, accelerating defect mapping and residual stress profiling [79]. Element-to-element spacing of 5–20 mm permits sufficient independent signal acquisition while maintaining continuous coverage; combined with automated electronic beam steering through appropriate element sequencing, linear arrays achieve two-dimensional spatial characterization from nominally one-dimensional probe configurations [79]. Two-dimensional sensor arrays—typically 4 × 4 or 8 × 8 matrix configurations—capture full surface maps during single measurement cycles, fundamentally transforming MBN from sequential point measurement methodology toward parallel imaging approaches [27]. Two-dimensional arrays require multi-channel acquisition electronics and sophisticated beam-forming algorithms to manage data volumes exceeding gigabits per measurement cycle; field-programmable gate array (FPGA) implementations enable real-time data processing, matching acquisition rates [27].

#### 3.4.3. Hybrid Sensor Systems Combining Multiple NDT Techniques

Hybrid multimodal sensor systems simultaneously acquire complementary information through integrated measurement techniques, substantially enhancing diagnostic discrimination compared to single-modality approaches [63]. Typical hybrid configurations combine (1) magnetic Barkhausen noise sensitive to dislocation and microstructural evolution; (2) eddy current impedance measurement sensitive to conductivity, permeability, and defect geometry; (3) ultrasonic techniques probing bulk elastic properties; and (4) harmonic analysis measuring nonlinear magnetic response [63]. Information fusion from multiple measurement modes enables multivariate material property prediction substantially more robust than individual technique predictions; combining three techniques reduces prediction uncertainty by approximately 30–50% compared to single-technique approaches [63].

#### 3.4.4. 3MA Technique: Multi-Frequency, Multi-Parameter Microstructure and Stress Analysis

The 3MA (Micromagnetic Multi-Parameter Microstructure and Stress Analysis) system represents a mature industrial implementation of multi-parameter magnetic characterization, systematically combining measurements from magnetic Barkhausen noise, harmonic tangential field analysis, and incremental permeability determination [76]. The 3MA methodology acquires >20 distinct measurement parameters sensitive to microstructural and stress conditions through synchronized hardware operation and subsequent computational analysis establishing multivariate regression relationships predicting material properties [76]. Fourth-generation 3MA equipment incorporates fully digital magnetic field control with vector feedback, enabling precise frequency and waveform optimization for specific applications; combined with advanced signal conditioning and feature extraction algorithms, 3MA systems achieve hardness prediction accuracies of ±20 HV (±2.4%) and residual stress determination of ±15 MPa (±3%) across industrial materials [76].

#### 3.4.5. Contactless MBN Sensors for Challenging Environments

Non-contact solenoid-type MBN sensors eliminate surface contact requirements through coaxial excitation and detection coil arrangements establishing approximate uniform magnetic fields within standoff distances of 1–10 mm [64]. Non-contact operation dramatically improves measurement repeatability through elimination of contact pressure variability, surface roughness effects, and surface conditioning dependencies—factors frequently dominating uncertainty budgets in contact measurements [64]. Contactless configurations enable inspection of (1) rough surfaces from grinding, shot-peening, or fatigue fracture; (2) curved or irregular geometries inaccessible to contact probes; (3) high-temperature components exceeding contact sensor material limitations; and (4) online monitoring during active manufacturing processes [64]. Trade-offs include reduced spatial resolution through standoff distance blur and increased sensitivity to lift-off distance variations requiring calibration correction [64].

#### 3.4.6. Wireless Sensor Networks for Distributed Monitoring

Wireless MBN sensor networks integrate compact magnetic measurement probes with low-power wireless communication (Bluetooth Low Energy, LoRaWAN) and onboard data processing, enabling distributed monitoring across geographically dispersed structural assets without installation of dedicated measurement infrastructure [26]. Passive energy harvesting technologies derive operational power from measurement frequency magnetic field oscillations, achieving multi-year battery-free operation; wireless data transmission at kilobit rates minimizes power consumption and enables adaptive sampling protocols adjusting measurement frequency based on detected material state changes [26]. Cloud integration architectures store time-series measurement data supporting longitudinal trend analysis; rolling-window anomaly detection algorithms identify material property evolution indicating advancing degradation or stress concentration development [26].

#### 3.4.7. Intelligent Detection Systems with AI Algorithms

Deep learning architectures—particularly convolutional neural networks operating on time–frequency spectrograms derived through STFT or wavelet analysis—automatically extract complex nonlinear features directly from raw Barkhausen signals without explicit feature engineering [4,22]. CNN-based stress prediction models achieve 95–98% accuracy across ±200 MPa stress ranges, substantially exceeding traditional linear/polynomial regression approaches; recurrent neural network variants incorporating temporal dependencies within avalanche sequences demonstrate enhanced performance on time-evolving material degradation scenarios [22]. Physics-informed neural networks incorporating domain knowledge constraints—such as known scaling law exponents—improve generalization to novel materials or stress states not adequately represented in training datasets, reducing overfitting vulnerabilities [22].

#### 3.4.8. Automated Scanning Systems and Robotic Integration

Automated scanning platforms combine MBN sensors with industrial robotic arms or gantry systems, enabling rapid high-resolution mapping of large component surfaces including complex geometries inaccessible to manual inspection [80]. Robotic integration with real-time defect detection algorithms enables automated sorting, directing non-conforming parts toward rework while conforming parts proceed toward subsequent manufacturing stages [80]. Measurement cycle automation eliminates operator variability; standardized probe positioning through robotic repeatability reduces measurement uncertainty, critical for high-volume production quality assurance [80].

#### 3.4.9. Integration with Manufacturing Processes for Real-Time Quality Control

In-process MBN monitoring during critical manufacturing stages—grinding, heat treatment, machining, and welding—enables real-time quality feedback and process correction [79]. Inline measurement systems installed directly at process stations continuously acquire MBN signals, detecting anomalies such as grinding burn, tool chatter, or inadequate heat penetration within minutes of occurrence rather than hours after completion [79]. Closed-loop control architectures adjust process parameters—spindle speed, feed rate, and quenching intensity—in response to detected material condition deviations, fundamentally transforming manufacturing from batch-level quality inspection toward continuous real-time process optimization supporting Industry 4.0 paradigms [79]. Industrial implementations report defect detection rates exceeding 99% with false-alarm rates < 1%, supporting reliable autonomous quality control without human intervention [79].

### 3.5. Strategies for Improving Repeatability and Reproducibility

Despite standardization efforts, achieving high repeatability in MBN measurements remains challenging due to the technique’s sensitivity to contact conditions, sensor lift-off, and magnetizing dynamics. Variations in probe orientation, surface roughness, and contact pressure can introduce significant measurement uncertainty. To address these limitations, several strategies have been established to enhance reproducibility:

- Automated Probe Handling: Replacing manual measurements with robotic or gantry-based positioning systems eliminates operator-dependent variability in sensor alignment and contact force. Automated systems can maintain constant lift-off (or controlled contact pressure) with high precision, reducing standard deviations in MBN amplitude from typical manual values of 10–15% to below 2–5%.
- Lift-Off Compensation Algorithms: For non-contact or varying-contact scenarios, establishing lift-off compensation functions is critical. By monitoring the intersection point of hysteresis loops or using simultaneous eddy current measurements to gauge distance, signal amplitudes can be mathematically corrected to a zero-lift-off reference state, significantly improving consistency on rough or curved surfaces.
- Magnetic Feedback Control: implementing active feedback loops in the magnetizing circuit ensures a constant flux density (B) or field (H) within the sample, regardless of variations in local permeability or coupling. Controlling the driving waveform to maintain a sinusoidal magnetic flux density (B-control) rather than just a sinusoidal excitation voltage (V-control) has been shown to drastically improve the reproducibility of MBN envelopes across different measurement setups.
- Surface Preparation Protocols: Standardizing surface cleaning and preparation procedures to remove non-conductive coatings or oxides ensures consistent electromagnetic coupling. Where surface modification is not possible, using lower excitation frequencies to increase skin depth can minimize the relative influence of surface variations.

## 4. Applications in Material Characterization

To clarify the positioning of magnetic Barkhausen noise within the broader family of magnetic NDT techniques, Table 1 summarizes the principal conceptual differences between MBN and more classical approaches such as hysteresis/coercivity measurements, magnetic flux leakage (MFL), eddy current (EC), and metal magnetic memory (MMM). While classical methods primarily probe bulk magnetization behavior or geometric discontinuities, MBN uniquely exploits the statistics of discrete domain wall avalanches, yielding high sensitivity to microstructure and residual stress in the near-surface region.

Table 2 provides a complementary star-rated comparison, emphasizing the relative advantages and limitations of each technique from an application perspective. MBN exhibits outstanding performance for microstructural and stress evaluation with fine spatial resolution, whereas MFL and EC remain superior for direct crack and wall loss detection, and hysteresis/MMM techniques are better suited for global condition and qualitative stress concentration assessment. These tables support the argument that MBN should be viewed as a complementary rather than competing method, particularly valuable when quantitative characterization of near-surface microstructure and residual stress is required in ferromagnetic components.

Table 3 provides a detailed operational comparison, distinguishing the quantitative capabilities and frequency-dependent depth resolution of MBN from the surface-limited or qualitative nature of traditional inspection methods.

### 4.1. Stress and Strain Measurements

The measurement of applied and residual stress through magnetic Barkhausen noise represents one of the most valuable practical applications of the technique across industrial material characterization and structural health monitoring [27,29]. The fundamental magnetoelastic coupling mechanism—whereby mechanical stress modifies magnetic anisotropy and domain wall dynamics—enables quantitative stress evaluation through systematic analysis of measured MBN parameters, advancing sensor technology for critical applications.

**Figure 3:**
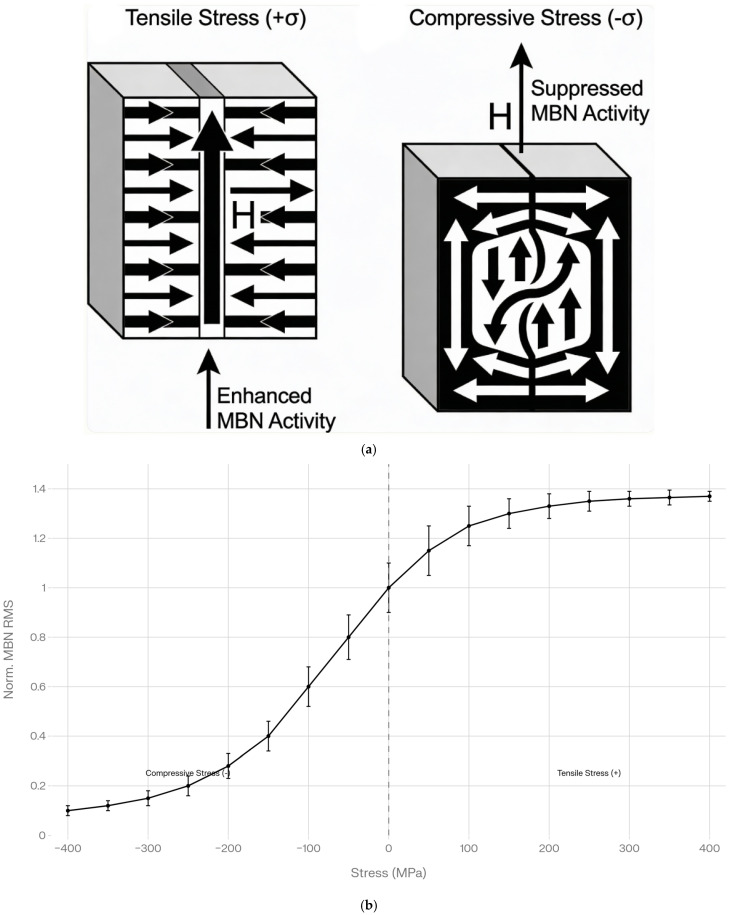
Analytical synthesis of stress–MBN correlations. (**a**) A schematic illustration of magnetoelastic coupling: tensile stress aligns domains with the magnetization direction (easy axis), enhancing domain wall dynamics, while compressive stress induces transverse alignment (hard axis), suppressing Barkhausen activity. (**b**) Typical normalized MBN RMS amplitude versus applied uniaxial stress calibration curve for structural steel, exhibiting characteristic asymmetry: saturation under high tension and rapid suppression under compression.

The fundamental dependence of magnetic Barkhausen noise on stress state is illustrated in Figure 3. Figure 3a schematically depicts the magnetoelastic coupling mechanism: Under tensile stress (for materials with positive magnetostriction like steel), magnetic domains align parallel to the stress axis, increasing the volume of domains favorably oriented with the applied field and enhancing MBN activity. Conversely, compressive stress forces domain alignment perpendicular to the stress axis, suppressing domain wall motion and reducing MBN intensity. Figure 3b presents a quantitative synthesis of this behavior, showing the characteristic asymmetric response curve where MBN amplitude increases monotonically with tension (saturating at high stresses due to domain alignment completion) and decreases rapidly under compression. This calibration curve forms the physical basis for quantitative residual stress evaluation in ferromagnetic components [2,81,82,83].

#### 4.1.1. Residual Stress Assessment: Tensile Versus Compressive

Residual stress evaluation distinguishes between tensile and compressive states through opposite effects on Barkhausen signal amplitude: tensile residual stresses increase MBN intensity through anisotropy reduction, whereas compressive stresses decrease intensity through anisotropy enhancement [84]. Subsurface tensile residual stress peaks—frequently dominant in ground components exhibiting grinding burn damage—generate pronounced MBN amplification despite potentially modest surface compressive stress, reflecting the depth-weighted electromagnetic sensitivity of MBN measurements [84]. Quantitative residual stress determination requires calibration against stress-certified reference samples through controlled tensile/compressive loading, establishing sigmoidal relationships between stress and MBN parameters spanning practical industrial stress ranges (typically ±200 MPa) [73].

#### 4.1.2. Self-Calibrating Stress Measurement Systems

Self-calibrating stress measurement systems eliminate manual calibration requirements through automated sample loading, MBN acquisition, and calibration surface computation [85]. The system applies programmed multi-directional stress states to calibration specimens, automatically acquiring corresponding MBN signals and calculating two-dimensional calibration surfaces relating in-plane stress components to measured noise intensity [85]. Advanced algorithms account for normal strain component effects directly rather than treating them as correction factors, substantially improving accuracy for multiaxial stress state determination [85]. Automated calibration dramatically reduces operator variability and enables rapid system recalibration for new material types [85].

#### 4.1.3. Advanced Sensor Configurations with High-Entropy Alloy Cores

High-entropy alloy (HEA) cores—incorporating FeCoNi and related multicomponent systems—demonstrate engineered magnetic properties superior to conventional ferrite for stress measurement applications [65]. HEA cores exhibit enhanced permeability uniformity across operational frequency ranges, reduced temperature-dependent permeability drift, and superior soft magnetic characteristics compared to traditional MnZn/NiZn ferrites [65]. Configuration of HEA cores in sensor probe assemblies improves stress sensitivity and measurement reproducibility through consistent flux concentration and reduced environmental dependencies [65].

#### 4.1.4. Non-Contact Stress Measurements: Solenoid-Type Sensors

Non-contact solenoid sensors operate at 1–10 mm standoff distances, eliminating contact pressure variability and surface condition dependencies that corrupt conventional contact measurements [64]. The directional coaxial architecture establishes approximately uniform magnetic fields through specimen cross-sections, enabling measurements on rough, curved, or thermally challenging surfaces inaccessible to contact probes [64]. Non-contact operation dramatically improves measurement repeatability; environmental round-robin studies demonstrate 5–8% measurement variation with non-contact sensors versus 12–18% with contact configurations [24].

#### 4.1.5. Thin-Film Stress Measurement

Thin ferromagnetic film stress characterization exploits the frequency-dependent electromagnetic skin effect to restrict MBN sensitivity to nanometer-to-micrometer surface layers [75]. Excitation frequency modulation from 5 kHz to 500 kHz modulates penetration depth from ~2 mm to ~10 μm, enabling stress profiling through layer-by-layer analysis [75]. Applications include residual stress mapping in surface treatments (shot-peening, nitriding), coating quality assurance, and grinding-induced damage characterization with unprecedented spatial resolution [75].

#### 4.1.6. Temperature Compensation Techniques

Temperature compensation addresses the fundamental challenge of MBN parameters varying with temperature through thermal modification of saturation magnetization and domain wall dynamics [86]. Dual-parameter measurement strategies—simultaneously acquiring MBN alongside temperature via embedded thermistors—establish mathematical compensation functions correlating temperature changes to signal evolution independent of stress [86]. Machine learning algorithms processing both MBN features and temperature data achieve stress prediction accuracy within ±15 MPa across −40–60 °C temperature ranges, a substantial improvement over single-parameter approaches [86]. Compensation effectiveness improves with multivariate feature extraction; utilizing multiple MBN parameters rather than single RMS values reduces temperature-induced prediction errors by approximately 90% [86].

#### 4.1.7. Residual Stress in Welded Structures

Welded joint residual stress characterization exploits MBN sensitivity to microstructural heterogeneity and stress redistribution inherent to fusion welding [87]. Weld bead center regions, typically exhibiting maximum tensile residual stress (~200 MPa, ~80% yield strength), generate peak MBN amplitudes; transitional heat-affected zones display intermediate characteristics; base metal regions with compressive residual stress show minimum noise [87]. Two-dimensional scanning maps residual stress distributions with sub-millimeter spatial resolution, enabling identification of stress concentration zones susceptible to fatigue crack initiation [87].

#### 4.1.8. Railway Stress Monitoring

Railway stress evaluation employs MBN to quantify longitudinal residual stresses affecting fatigue and fracture characteristics critical for operational safety [88]. Laboratory calibrations establishing MBN–stress relationships for specific rail grades enable field measurements characterizing residual stress distributions through rail cross-sections and along extended track lengths [88]. Applied load sensitivity correlations established through uniaxial tensile tests facilitate stress determination with ±15 MPa uncertainty, suitable for condition monitoring and predictive maintenance [88]. Asymmetric loading patterns from wheel contact geometry are detectable through directional measurement variations, enabling more realistic stress state reconstruction [88].

#### 4.1.9. In Situ Monitoring During Mechanical Testing

Real-time MBN monitoring during tensile/compressive loading simultaneously captures stress and microstructure evolution, revealing distinct behavior in elastic versus plastic deformation regimes [27]. In the elastic regime, tensile stress generally increases MBN intensity (in materials with positive magnetostriction) by aligning domains along the measurement axis, whereas compressive stress suppresses it. In contrast, plastic deformation typically reduces MBN intensity regardless of the loading direction, due to dislocation multiplication and grain boundary migration that pin domain walls, providing mechanistic insight into material damage evolution [27]. Time–response histogram analysis distinguishes intragranular versus grain boundary domain dynamics under stress, identifying stress concentration locations prone to crack initiation [27].

#### 4.1.10. Prestressed Concrete Strand Monitoring

Smart strand technology—integrating embedded elastomagnetic sensors into prestressed concrete tendons—enables continuous tension monitoring from construction through service life [89,90]. Magnetoelastic inductance changes directly correlate with strand tension through permeability modifications; field deployments over 15+ months demonstrate stable long-term performance with temperature-compensated measurements achieving ±5% tension estimation accuracy [90]. Sensor-based methods eliminate periodic manual measurements, providing early warning of abnormal prestress loss indicative of tendon damage or anchorage slip [90].

#### 4.1.11. Multiaxial Stress State Determination

Multiaxial stress evaluation requires multiple directional measurements—typically three non-coplanar orientations—to decompose measured MBN vectors into principal stress components [85]. Calibration surfaces relating three-dimensional MBN response to two-dimensional in-plane strain/stress components enable the reconstruction of complex stress states including magnitude, direction, and principal component identification [85]. Practical applicability extends to components experiencing complex loading including combined tension–torsion or multiaxial fatigue conditions [85].

### 4.2. Microstructural Analysis

Microstructural characterization through magnetic Barkhausen noise represents a sophisticated non-destructive methodology bridging materials science and electromagnetic phenomena, enabling quantitative evaluation of grain structure, dislocation distributions, phase compositions, and surface treatments [27,28]. The sensitivity of MBN to microstructural evolution provides advanced diagnostic capabilities for quality control and materials research aligned with the *Sensors* journal’s focus on sensor applications for industrial characterization.

#### 4.2.1. Grain Size Evaluation: Domain Wall Spacing Model

The domain wall spacing model establishes fundamental relationships between grain size and MBN through electromagnetic domain organization within crystalline grains. Magnetic domain widths scale inversely with saturation magnetization M_s_ and directly with exchange stiffness A and magnetic anisotropy K_1_ through $D=\sqrt{\frac{A}{K_{1}}}$ [28]. In polycrystalline materials, domain wall density ρ_DW_ (number of walls per unit area) scales approximately as $\rho_{DW}\propto d^{-2}$, where d denotes average grain size [28]. The inverse power-law relationship between MBN amplitude and grain size is ${\mathrm{MBN}}_{\mathrm{envelope}}\propto d^{-\alpha }$, where the exponent α typically ranges from 0.5 to 1.0 depending on material composition and stress state [28]. Fine-grained materials exhibit systematically higher domain wall densities per unit volume, producing enhanced irreversible magnetization contributions and increased MBN intensity [28].

Signal-to-noise considerations become critical in grain size measurements; materials exhibiting fine grains (d < 5 μm) generate high MBN amplitudes with favorable signal-to-noise ratios (>20:1), whereas coarse-grained materials (d > 100 μm) produce weak signals approaching noise floor levels, limiting quantitative accuracy to ±8–10 μm in extreme cases [28]. Frequency-dependent effects modulate sensitivity through electromagnetic skin effect depth variations; low-frequency excitation (5–10 Hz) emphasizes bulk grain structure, while high-frequency components (50–200 kHz) enhance near-surface microstructural sensitivity [28].

#### 4.2.2. High-Resolution Grain-Scale Measurements

Grain-scale MBN measurements exploit shielded pickup coil architectures achieving 2–5 mm lateral spatial resolution, enabling direct observation of domain wall dynamics within individual crystallographic grains and at grain boundary interfaces [27]. Time–response histogram analysis—processing MBN signals through synchronized temporal windows corresponding to specific magnetization phases—distinctly separates intragranular from grain boundary phenomena: intragranular regions display rapid, relatively uniform signal evolution reflecting homogeneous domain wall motion, whereas grain boundaries exhibit complex, multi-phase temporal structures reflecting heterogeneous pinning distributions [27]. High-spatial-resolution mapping across component surfaces reveals grain-to-grain MBN variations of 30–50%, reflecting orientation-dependent magnetic anisotropy effects [27].

#### 4.2.3. Phase Identification and Composition Analysis

Duplex stainless steel characterization exemplifies MBN’s capacity for phase identification through ferrite–austenite discrimination: MBN signals originate exclusively from ferromagnetic ferrite phases; austenitic (non-magnetic) regions contribute zero signal intensity [91]. Progressive sigma phase formation during aging treatments progressively reduces ferrite volume fractions, producing proportional MBN intensity reductions approaching noise floor levels after ~24 h aging at 850 °C, enabling quantitative ferrite volume tracking [91]. This composition-sensitive behavior establishes MBN as a viable non-destructive alternative to destructive ferritoscope or metallographic quantification techniques.

Crystallographic texture effects in grain-oriented electrical steels strongly modulate MBN directional sensitivity through magnetocrystalline anisotropy variations: conventional grain-oriented (CGO) sheets with random grain orientations display relatively isotropic MBN characteristics; high-permeability grain-oriented (HGO) sheets exhibiting strong (110) Goss texture demonstrate anisotropic MBN with 2–3× the signal intensity variation between rolling and transverse directions [4]. Short-time Fourier transform spectrograms reveal that maximum MBN activity concentrates along preferred easy axes, enabling crystallographic orientation determination through systematic angular measurements [4].

#### 4.2.4. Dislocation Density Measurements

Individual dislocation effects on domain wall motion reflect magnetoelastic coupling through strain fields surrounding dislocation cores, creating localized anisotropy variations acting as pinning sites. Micromagnetic simulations reveal that single edge dislocations establish energy barriers of 5–20 meV for domain wall interactions, with pinning strength scaling strongly with Burgers vector orientation and wall type [35]. Dislocation network collective behavior becomes dominant at densities exceeding ~10^12^ m^−2^ (typical plastic deformation thresholds), where distributed pinning sites create statistically significant resistance to domain wall motion [92].

The power-law relationship between dislocation density ρ_d_ and MBN amplitude exhibits ${\mathrm{MBN}}_{\mathrm{RMS}}\propto \rho_{d}^{-0.6–0.8}$, indicating strong suppression of Barkhausen noise through enhanced pinning strength in heavily deformed materials [92]. Quantitatively, MBN amplitude decreases by approximately 1% per 10^13^ m^−2^ dislocation density increment in ferritic steels, establishing a practical quantitative relationship enabling non-destructive dislocation density assessment [92].

#### 4.2.5. Surface Treatment Characterization

Grinding burn detection represents an established industrial MBN application exploiting near-surface stress and microstructural modifications accompanying grinding-induced thermal damage [84]. Grinding burns generate characteristic signatures: surface compressive residual stresses (typically −100 to −300 MPa) produce MBN suppression, while subsurface tensile stresses (occasionally +200 to +500 MPa from excessive grinding heat) produce competing MBN enhancement [84]. The net observed effect depends on penetration depth weighting; subsurface tensile stresses typically dominate MBN measurements, producing apparent signal amplification despite surface compressive stress presence [84]. Multi-parametric analysis utilizing envelope peak position, amplitude, and pulse count provides superior burn discrimination (90–95% detection rates) compared to single-parameter RMS analysis (65–75%) [84].

Surface treatment effects including shot-peening and nitriding produce characteristic MBN signatures: shot-peening introduces surface compressive residual stresses and dislocation-hardened layers, reducing MBN amplitude by 30–50%; subsequent thermal relaxation gradually restores baseline MBN through stress relief, enabling condition monitoring during service [84]. Nitrided layers exhibit a binary nature: shallow case-hardened regions with a transformed microstructure produce reduced ferromagnetism, while the core material retains ferromagnetic properties, enabling depth-sensitive analysis through frequency modulation [93].

#### 4.2.6. Integration with Complementary Characterization Techniques

Hybrid characterization strategies combining MBN with complementary techniques substantially enhance diagnostic discrimination:

- Magneto-optical Kerr effect (MOKE) microscopy directly visualizes domain wall motion and magnetic domain structure contemporaneously with MBN measurements, providing mechanistic validation of signal origins [27].
- Electron backscatter diffraction (EBSD) quantifies crystallographic orientation distributions and tilt angles, enabling correlation with MBN anisotropic characteristics in textured materials [4].
- X-ray diffraction (XRD) establishes residual stress profiles and dislocation densities through peak broadening analysis, validating MBN stress–dislocation relationships [84].
- Scanning electron microscopy (SEM) characterizes microstructural features including precipitate distributions, phase morphologies, and grain boundary configurations, complementing MBN sensitivity [94].
- Vickers/nanoindentation hardness correlates with MBN-derived microstructural parameters, establishing property prediction relationships [3].

These integrated approaches leverage MBN’s non-destructive nature alongside direct microstructural observations, substantially improving the reliability of material property assessments for advanced manufacturing quality assurance and condition monitoring applications.

### 4.3. Deformation and Plastic Flow Studies

The in situ monitoring of material deformation through magnetic Barkhausen noise provides unprecedented capabilities for real-time characterization of plastic flow, work hardening evolution, and fatigue damage progression, establishing MBN as a critical tool for understanding deformation mechanisms and optimizing forming processes [27,95]. The integration of MBN with mechanical testing equipment enables direct observation of elastic–plastic transitions and microstructural evolution during controlled loading, advancing sensor technology for process control and structural health monitoring, aligning with the *Sensors* journal’s emphasis on industrial sensor applications.

#### 4.3.1. Physical Basis: Dislocations–Domain Wall Interactions

Dislocations–domain wall interactions represent the fundamental mechanism coupling mechanical deformation with MBN signal evolution [35]. Dislocations, quantified through density measurements ranging from 10^10^ m^−2^ in annealed materials to >10^15^ m^−2^ in heavily strained specimens, establish elastic strain fields creating magnetoelastic pinning potentials that obstruct domain wall motion [92]. The orientation-dependent interaction strength reflects the magnetic coupling geometry: optimally oriented dislocations perpendicular to domain wall propagation direction produce maximum pinning (20–100 meV barriers), substantially exceeding single-point defect pinning (5–20 meV) [35]. As plastic deformation progresses, dislocation multiplication accelerates; strain increments of 1–5% generate 5 × 10^12^ to 10^13^ m^−2^ dislocation density increases, proportionally suppressing MBN amplitude through progressive pinning enhancement [27].

#### 4.3.2. In Situ Measurements During Tensile Testing

Real-time MBN monitoring during uniaxial tensile testing reveals distinct elastic and plastic regime characteristics [27]. In the elastic regime (strain ε < ε_Y_, where ε_Y_ denotes yield strain), MBN amplitude increases approximately linearly with applied stress through magnetoelastic anisotropy reduction, enabling a stress sensitivity of ±5–20 mV per 100 MPa [27]. Elastic regime MBN exhibits a reproducible, reversible nature through repeated loading–unloading cycles, with negligible permanent signal evolution upon stress removal [27].

Plastic regime behavior (ε > ε_Y_) exhibits markedly different characteristics: MBN amplitude progressively decreases with increasing plastic strain as dislocation accumulation progressively enhances pinning strength and restricts domain wall avalanche sizes [27]. The MBN envelope shifts toward higher magnetic fields as pinning strength increases, with characteristic shifts of 5–15 kA/m per percent plastic strain in ferritic steels [27].

#### 4.3.3. Elastic–Plastic Transition Detection

Sharp transition characteristics at yield points provide distinctive MBN signatures enabling precise elastic–plastic boundary identification [27]. MBN intensity exhibits a characteristic maximum (20–40% amplitude increase relative to elastic baseline) near the yield point, reflecting the competing effects of stress-induced anisotropy reduction (promoting MBN) and initial dislocation multiplication (suppressing MBN) occurring simultaneously [27]. Subsequent plastic strain evolution produces continued MBN suppression as dislocation density dominates over stress anisotropy effects, creating a distinctive “humped” MBN–strain curve with maximum near strain = 0.3–0.5% [27]. Time–response histogram analysis reveals that peak MBN activity concentrates at the elastic–plastic boundary, with intragranular versus grain boundary signal partitioning fundamentally changing across this transition [27].

#### 4.3.4. Work Hardening Characterization Across Different Stages

Multi-stage work hardening analysis demonstrates that MBN evolution quantitatively tracks microstructural evolution through distinct hardening stages [28]. Stage I (linear hardening, ε < 2–3%): dislocation forest strengthening dominates; MBN suppression follows approximately linear relationships with plastic strain [28]. Stage II (parabolic hardening, 3–10% strain): dislocation cell formation and boundary organization intensify; MBN suppression accelerates through collective pinning effects as organized dislocation networks establish more efficient barriers [28]. Stage III (saturation, ε > 10%): dynamic recrystallization and recovery begin competing with work hardening; MBN evolution reaches a plateau as competing mechanisms produce a relatively constant dislocation density and pinning landscape [28].

#### 4.3.5. Cyclic Deformation and Fatigue Damage Detection

Cyclic loading responses demonstrate that MBN parameters sensitively track fatigue damage evolution across fatigue lifetime stages [95,96]. In early fatigue stages (N < 0.1 N_f_, where N_f_ denotes cycles to failure), MBN exhibits an initial decrease reflecting cyclic hardening and dislocation structure refinement; magneto-elastic parameters display an oscillatory nature, reflecting periodic material responses to cyclic stressing [96]. Mid-fatigue stages (0.1 N_f_ < N < 0.7 N_f_) show relatively stable MBN characteristics as the cyclic steady-state microstructure persists; shallow microcrack initiation begins producing secondary effects undetected by traditional MBN parameters [96].

Late-stage fatigue monitoring (N > 0.7 N_f_) reveals sharp MBN signal reductions corresponding to macroscopic crack growth through 20–60% amplitude suppression near final failure, enabling crack detection before critical fracture [95]. The temporal evolution of MBN distinctly identifies fatigue damage stages, with early warning of final failure possible through MBN time-derivative analysis [95].

#### 4.3.6. Multi-Directional Measurements for Plastic Anisotropy

Directional MBN measurements reveal plastic anisotropy development through crystallographic texture evolution and preferential dislocation organization [28]. In rolling direction (RD) versus transverse direction (TD) measurements on sheet materials, work-hardened specimens exhibit pronounced magnetic anisotropy, with rolling direction MBN suppressed and transverse direction MBN enhanced (2–3× intensity variation) due to preferential domain wall reorientation into TD easy axes [28]. The degree of magnetic anisotropy scales with plastic strain, increasing from a near-isotropic response at ε = 0 to pronounced (>3:1) anisotropy at ε = 20% plastic strain [28].

#### 4.3.7. Temperature Effects During Deformation

Temperature-dependent deformation reveals competing thermal effects: decreased saturation magnetization (M_s_) with temperature through Bloch power-law evolution reduces MBN intensity, while enhanced thermally activated dislocation motion modifies pinning dynamics [27]. Elevated-temperature deformation (T = 100–200 °C) shows systematically reduced MBN suppression per unit plastic strain compared to room-temperature deformation, reflecting reduced dislocation immobility through thermal pinning assistance [27]. Cryogenic deformation (T= −50 °C) produces enhanced MBN suppression exceeding room-temperature trends, consistent with immobilized dislocation populations preventing recovery [27].

#### 4.3.8. Monitoring of Severe Plastic Deformation (SPD) Processes

Severe plastic deformation monitoring through MBN enables non-destructive characterization of ultra-fine-grained material production [97]. High-pressure torsion (HPT) and equal-channel angular extrusion (ECAE) processes generate plastic strains exceeding ε_true_ = 50–100, producing dislocation densities approaching 10^16^ m^−2^, accompanied by complete MBN signal suppression approaching noise floor levels [97]. MBN provides quantitative indicators of SPD extent: materials achieving true strains ε = 10–20 display 30–50% MBN amplitude retention, while ε > 50 produces 80–95% amplitude suppression [97].

#### 4.3.9. Additive Manufacturing Process Applications

In situ MBN monitoring during laser powder bed fusion (LPBF) additive manufacturing enables real-time residual stress control during layer-by-layer material accumulation [30]. Maraging steel 300 components measured immediately after deposition show residual stress-dependent MBN variations, enabling process parameter feedback for stress mitigation [30]. Multi-layer monitoring tracks stress redistribution as subsequent layer thermal cycles alter underlying material residual stress states through annealing effects, providing process control feedback [30].

#### 4.3.10. Local Plastic Deformation Detection

High-spatial-resolution MBN sensors (2–5 mm measurement volumes) enable local plastic deformation mapping through grain-scale characterization of strain localization patterns [27]. Strain-localized zones associated with potential crack initiation sites exhibit distinctly different MBN responses compared to uniformly strained regions, enabling predictive damage location identification before macroscopic failure [27].

#### 4.3.11. Process Control in Forming Operations

Automated inline MBN monitoring integrated with manufacturing process equipment enables real-time quality feedback and process parameter adjustment [79]. Deep drawing, stamping, and extrusion operations employ MBN measurements characterizing localized hardness, strain distribution, and residual stress; detected anomalies trigger process adjustments (spindle speed modification, coolant system tuning) to maintain quality specifications [79]. Industrial implementations report defect detection rates exceeding 95% with <1% false-alarm rates, supporting autonomous closed-loop quality control [79].

### 4.4. Phase Transformation Monitoring

The phase transformation monitoring capability of magnetic Barkhausen noise represents a powerful application enabling non-destructive tracking of microstructural evolution during heat treatment, welding, and in-service exposure [91,98]. The fundamental sensitivity of MBN to ferromagnetic phase volume fractions and compositional variations provides quantitative characterization of phase decomposition, precipitation kinetics, and harmful intermetallic formation across diverse industrial materials.

#### 4.4.1. Sensitivity to Different Magnetic Phases: Ferrite, Martensite, and Austenite

Phase-specific MBN responses reflect the distinct magnetic characteristics of ferromagnetic versus paramagnetic constituents [98]. Ferrite—the ferromagnetic body-centered cubic (BCC) iron phase—generates strong Barkhausen signals reflecting unrestricted domain wall motion; typical ferrite signals exhibit MBN envelope peaks at relatively low magnetic field strengths (5–10 kA/m) [98]. Martensite—the body-centered tetragonal (BCT) phase formed through displacive transformation from austenite—generates weaker MBN than ferrite due to tetragonal lattice distortion and internal stress distributions restricting domain wall dynamics; martensite envelope peaks shift to higher magnetic field positions (20–35 kA/m) with reduced amplitude proportional to carbon content [98]. Austenite—the paramagnetic face-centered cubic (FCC) phase—generates zero Barkhausen signal, providing clear phase identification through the absence of MBN response [98].

#### 4.4.2. Austenite Formation and Decomposition During Heat Treatment

Austenitization during heat treatment transitions ferrite → austenite above the critical transformation temperature (A_c3_ ≈ 920 °C for plain carbon steels), producing proportional MBN signal reduction, reflecting austenite formation [98]. Complete austenitization produces essentially zero MBN amplitude as the ferromagnetic ferrite phase entirely converts to paramagnetic austenite [98]. Tempering processes after quenching generate reverse austenite precipitation from martensite, observable through MBN evolution during isothermal holding as the austenite fraction increases [98]. The kinetics of reverse transformation exhibit temperature dependence: higher tempering temperatures (>350 °C) accelerate reverse austenite formation, producing rapid MBN decrease rates of 2–5% signal loss per 10 min holding time [98].

#### 4.4.3. Martensitic Transformation Characterization

Martensitic transformation from austenite generates distinctive MBN signatures through stress-induced domain structure modifications accompanying the displacive phase change [99]. Quenching-induced martensite formation produces MBN signal reappearance after complete austenitization, with signal amplitude progressively increasing with martensite volume fraction, following approximately $\mathrm{MBN}\propto V_{m}^{0.7–0.9}$, where V_m_ denotes martensite volume fraction [99]. Temperature effects on martensitic transformation kinetics modulate MBN response; lower quenching temperatures (liquid nitrogen) produce higher martensite content and stronger MBN, while higher quenching temperatures restrict transformation kinetics [99].

#### 4.4.4. Precipitation Processes in Age-Hardenable Alloys

Precipitation kinetics monitoring through MBN enables non-destructive tracking of hardening particle formation during controlled aging heat treatments [100]. Age-hardenable systems (17-4 PH stainless steel, nickel-based superalloys, aluminum–copper alloys) exhibit distinctive MBN evolution through precipitation stages: under-aged conditions show gradual MBN reduction as coherent precipitate formation induces localized anisotropy variations; peak-aged conditions approach saturation as precipitate volume fraction plateaus; over-aged conditions exhibit MBN recovery through precipitate coarsening reducing local pinning effectiveness [100]. Time–temperature transformation (TTT) diagram mapping correlates MBN evolution with precipitation kinetics, enabling the determination of optimal heat treatment schedules for target mechanical properties [100].

#### 4.4.5. Harmful Intermetallic Phase Detection: Sigma and Chi Phases in Duplex Stainless Steels

Sigma phase formation in duplex stainless steels during thermal aging above 550 °C represents a critical industrial concern due to severe embrittlement, despite minimal volume fractions; MBN sensitivity to ferrite decomposition enables early detection before mechanical property deterioration [91]. As the sigma phase (paramagnetic) precipitates from ferrite (ferromagnetic), the ferrite volume fraction progressively decreases, producing proportional MBN amplitude reductions: 24 h aging at 850 °C produces ~90% MBN suppression, reflecting nearly complete ferrite → sigma transformation [91]. Chi phase detection follows similar mechanisms; combined sigma + chi formation produces complete MBN signal loss (approaching background noise levels) via ~50 h thermal aging, enabling practical field component condition monitoring [91].

#### 4.4.6. Temperature-Dependent Phase Stability Monitoring

In-service high-temperature component monitoring exploits temperature-dependent phase stability variations through systematic MBN measurements at operational temperatures or after controlled cooling following extended exposure [98]. Creep-resistant superalloys experience γ′ (Ni_3_Al) precipitate coarsening during prolonged high-temperature service, observable through MBN evolution reflecting secondary phase precipitation/dissolution kinetics [98]. Real-time MBN monitoring during thermal cycling reveals phase instability development through characteristic MBN fluctuations accompanying reverse transformations and competing phase precipitation [98].

#### 4.4.7. Quantum Effects in Low-Temperature Phase Transformations

Quantum tunneling effects in low-temperature martensitic transformations (cryogenic quenching at T < 77 K) modulate transformation kinetics and microstructure through modified diffusion pathways and reduced thermal activation requirements [99]. MBN measurements at cryogenic temperatures reveal enhanced martensite formation rates and a modified defect structure, reflecting quantum mechanical processes competing with classical thermal activation [99].

#### 4.4.8. Kinetics of Phase Transformations: Isothermal and Non-Isothermal

Isothermal transformation tracking (constant-temperature aging) produces predictable MBN evolution following Avrami-type kinetics, $V_{\mathrm{phase}}=1-exp(-kt^{n})$, where k and n denote transformation rate constants and the Avrami exponent [98]. Non-isothermal transformations (continuous cooling, heating) exhibit more complex MBN behavior, reflecting competing transformation pathways; continuous heating through austenite’s stability region produces rapid MBN decrease rates as austenite formation progressively reduces ferrite volume [98].

#### 4.4.9. Advanced Signal Processing for Phase Separation

Principal component analysis (PCA) applied to multi-parameter MBN datasets (amplitude, peak position, integral, spectral centroid) enables automated phase identification and quantification through multivariate pattern recognition independent of single-parameter ambiguities [101]. Wavelet-based feature extraction revealing time–frequency characteristics specific to individual phases (ferrite, martensite, austenite) enables robust phase discrimination even in complex multi-phase materials [101].

#### 4.4.10. In-Service Monitoring of High-Temperature Components

Extended service monitoring of power generation turbines, pressure vessels, and aerospace structures through periodic MBN measurements enables the detection of harmful phase precipitation before critical property loss occurs [91]. Field deployments demonstrate MBN’s ability to identify sigma/chi phase formation in duplex stainless steel piping at <3% phase volume fractions, well below mechanical property threshold values, enabling preventive maintenance scheduling [91].

## 5. Correlations with Material Properties

### 5.1. Grain Size and Texture Effects

**Figure 4:**
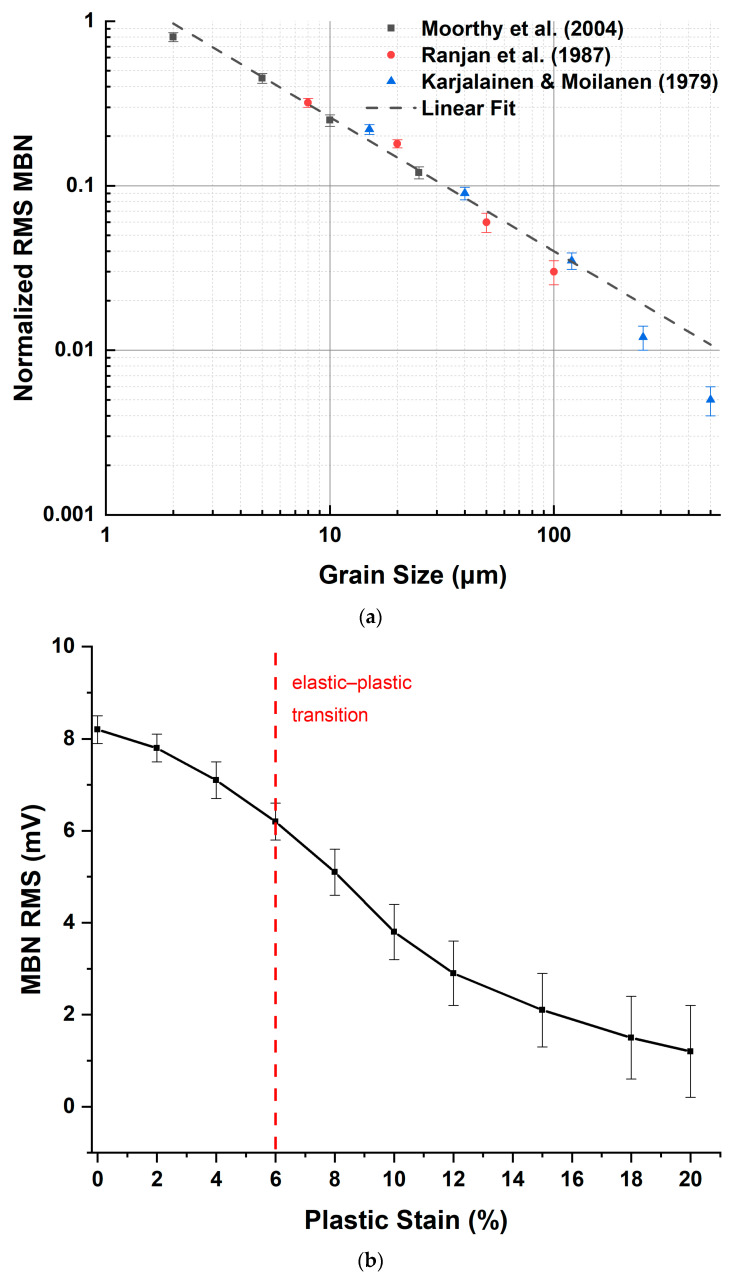

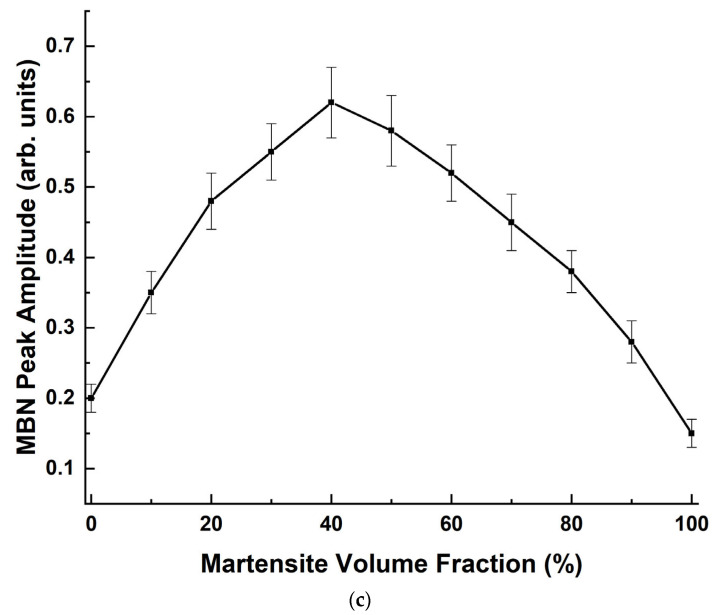
Analytical synthesis of MBN parameters versus key microstructural features from representative studies on stress-free ferromagnetic steels. (**a**) Log–log plot of normalized MBN RMS versus grain size (1–1000 μm) showing universal power-law scaling $MBN_{RMS}\propto d_{g}^{-0.7}$ (solid line), consistent with results reported for ferritic steels [103,104,109]; (**b**) MBN RMS versus plastic strain (0–20%) exhibiting an initial decrease followed by elastic–plastic transition near 6% strain; (**c**) MBN peak amplitude versus martensite volume fraction (0–100%) showing non-monotonic sensitivity peaking at intermediate phase fractions.

The quantitative relationships between MBN parameters and microstructural features are illustrated in Figure 4, which synthesizes representative experimental trends reported across multiple studies. Figure 4a demonstrates the universal power-law dependence of normalized MBN RMS on grain size observed in ferritic steels, with $MBN_{RMS}\propto d_{g}^{-0.7}$ (where $d_{g}$ represents average grain diameter), consistent with comprehensive grain refinement studies [102,103]. This scaling behavior reflects the domain wall spacing model, where coarser grains permit larger avalanche events due to reduced grain boundary pinning density. Figure 4b illustrates the evolution of MBN RMS with accumulated plastic strain, showing an initial monotonic decrease followed by accelerated suppression beyond the elastic–plastic transition (~6% strain), characteristic of dislocation multiplication during deformation hardening [104]. Figure 4c reveals the non-monotonic sensitivity to the martensite volume fraction, peaking at intermediate phase contents (40–60%) due to optimal magnetic heterogeneity enhancing domain wall pinning interactions between ferrite and martensite phases [105,106,107]. These established correlations provide quantitative benchmarks for MBN-based microstructural characterization across diverse alloy systems and processing conditions [28,108].

#### 5.1.1. Domain Wall Spacing Model and Theoretical Relationships

The domain wall spacing model establishes the fundamental relationship between grain geometry and magnetic domain organization within polycrystalline ferromagnets [28]. The magnetic domain width scales as $D=\sqrt{\frac{A}{K_{1}}}$, where A denotes exchange stiffness and K_1_ represents magnetocrystalline anisotropy energy [28]. Within crystallographic grains bounded by high-angle boundaries, multiple magnetic domains subdivide the grain volume; the number of domain walls per unit area scales approximately as $\rho_{DW}\propto d^{-2}$, where d denotes average grain diameter [28]. This fundamental relationship translates directly to MBN sensitivity: increased domain wall density in finer-grained materials produces greater numbers of irreversible domain wall displacements during magnetization, generating proportionally stronger Barkhausen signals [28].

#### 5.1.2. Power-Law Dependence on Grain Size (*n* = 0.5–1.0)

The quantitative grain size–MBN relationship follows ${\mathrm{MBN}}_{\mathrm{envelope}}\propto d^{-n}$, where the exponent n typically ranges from 0.5 to 1.0 depending on material composition, stress state, and measurement methodology [28]. Ferritic steels with uniform microstructure exhibit exponent values *n* ≈ 0.5–0.7, reflecting domain wall density scaling; dual-phase materials containing martensite exhibit steeper dependencies (*n* ≈ 0.8–1.0), reflecting competing grain refinement and phase transformation effects [28]. The power-law relationship enables quantitative grain size determination, $d=C\times {\mathrm{MBN}}^{-1/n}$, where C denotes a material-dependent calibration constant [28].

#### 5.1.3. Effects of Measurement Noise on Accuracy

Signal-to-noise considerations critically impact grain size measurement accuracy, particularly for coarse-grained materials (d > 100 μm) generating weak MBN amplitudes approaching instrumental noise floor levels [28]. Fine-grained materials (d < 5 μm) exhibit high MBN amplitudes with favorable signal-to-noise ratios (>20:1), enabling accurate grain size determination to ±5–8 μm precision; coarse-grained materials produce measurement uncertainties exceeding ±15–20% due to reduced signal magnitude relative to electronic noise [28]. Frequency-dependent measurement strategies modulate effective noise characteristics; high-frequency excitation (200–500 kHz) produces enhanced surface sensitivity but reduced signal-to-noise ratios, while low-frequency operation (5–50 Hz) provides superior bulk sensitivity with improved noise margins [28].

#### 5.1.4. Influence of Nature of Grain Boundary Distribution

The nature of grain boundary distribution—quantifying high-angle versus low-angle grain boundary proportions and misorientation angle distributions—significantly influences MBN characteristics beyond simple average grain size effects [27]. High-angle grain boundaries (>15° misorientation) create pronounced magnetic dipole concentrations and strong domain wall pinning sites; materials with high-angle boundary-dominated structures exhibit enhanced MBN compared to low-angle boundary-dominated microstructures despite similar average grain sizes [27]. Kernel average misorientation (KAM) mapping via electron backscatter diffraction (EBSD) enables quantitative grain boundary characterization; correlations with MBN reveal that KAM values > 2° (indicating high-angle boundary prevalence) produce 20–40% higher MBN amplitudes compared to low-KAM (<1°) microstructures [27].

#### 5.1.5. High-Spatial-Resolution Observations at Grain Scale

Micromagnetic grain-scale observations through high-resolution MBN sensors (2–5 mm measurement volumes) reveal heterogeneous domain wall dynamics across individual crystallographic grains and grain boundary regions [27]. Time–response histogram analysis demonstrates that grain boundaries generate distinctly different MBN temporal signatures: intragranular regions display rapid, relatively uniform signal evolution; grain boundary regions exhibit complex, multi-phase temporal structures reflecting heterogeneous pinning distributions [27]. Two-dimensional scanning across component surfaces reveals grain-to-grain MBN variations of 30–50%, reflecting anisotropy-driven heterogeneity despite relatively uniform grain sizes [27]

#### 5.1.6. Crystallographic Texture and Anisotropy: Magnetocrystalline Anisotropy Energy

Magnetocrystalline anisotropy energy (MAE)—the energy cost for magnetization along different crystallographic directions—fundamentally determines MBN directional sensitivity in textured materials [108]. In cubic ferromagnets, easy axes preferentially align along <100> directions; MAE for magnetization along <100> versus <111> directions differs by several orders of magnitude (>10^5^ J/m^3^), establishing strong crystallographic preference for magnetization orientation [108]. Grain-oriented electrical steels exhibiting strong Goss texture (crystallographic plane parallel to rolling direction, <100> direction perpendicular to rolling direction) demonstrate pronounced magnetic anisotropy; rolling direction magnetization saturates easily while transverse direction magnetization requires substantially higher applied fields [108].

#### 5.1.7. Angular Dependence of MBN Parameters

Directional MBN measurements reveal angle-dependent intensity variations reflecting magnetocrystalline anisotropy through magnetic easy/hard axis alignment [74,108]. The MBN envelope’s maximum amplitude typically varies by 2–3× (approximately 300% variation) across the full angular sweep (0–180°) in highly textured materials; grain-oriented silicon steel measurements demonstrate sharp MBN maxima aligned with the rolling direction and sharp minima in the transverse direction, reflecting Goss texture <100> easy axis orientation perpendicular to the rolling plane [74]. The angular MBN energy function approximately follows ${\mathrm{MBN}}_{\mathrm{energy}}(\theta )=I\times [\cos^{3}(\theta -\phi_{1})+\cos^{3}(\theta -\phi_{2})]$, where φ_1_, φ_2_ denote easy axis positions and I represents the intensity scaling factor [108].

#### 5.1.8. Rolling and Drawing Textures

Rolling-induced texture—primarily {110}<112> and {112}<111> texture components—produces moderate magnetic anisotropy in non-oriented steels through alignment of <100> easy axes; MBN anisotropy ratios (maximum/minimum) typically reach 1.5–1.8× [108]. Drawing-induced texture—primarily {111}<110> fibrous texture—generates weaker magnetic anisotropy compared to rolling since <111> hard axes align preferentially, producing MBN anisotropy ratios ~1.2–1.3× [108]. Grain-oriented electrical steels with strong Goss texture achieve MBN anisotropy ratios exceeding 3–4×, enabling superior permeability along the rolling direction essential for transformer core applications [108].

#### 5.1.9. Recrystallization Process Monitoring

In situ recrystallization monitoring during annealing employs MBN to track microstructural evolution through distinct stages: recovery (dislocation rearrangement without new grain formation), recrystallization (new defect-free grain nucleation and growth), and grain growth (coarsening of fully recrystallized structure) [110]. MBN parameters exhibit distinctive evolution patterns: during recovery (<300 °C for iron), progressive MBN suppression reflects dislocation density reduction (~10–15% MBN decrease per 1% dislocation density reduction); during recrystallization (300–600 °C), complex MBN behavior reflects competing effects of dislocation elimination (MBN increase) versus grain coarsening (MBN decrease), producing characteristic MBN valleys at intermediate recrystallization fractions; post-recrystallization grain growth produces gradual MBN decreases, reflecting continued coarsening [110].

#### 5.1.10. Separating Grain Size and Texture Contributions

Multivariate analysis of angular MBN measurements enables separation of grain size and texture effects through directional sensitivity differences: grain size contributions scale isotropically (similar scaling in all directions), while texture effects exhibit strong angular dependence peaking along easy axes [108]. Principal component analysis applied to multi-directional MBN measurements (typically 8–16 angles spanning 0–180°) enables decomposition into texture-weighted and isotropic contributions; modern approaches achieve > 80% discrimination accuracy between microstructural versus texture-driven MBN variations [108].

#### 5.1.11. Temperature Effects on Relationships

Temperature-dependent MBN scaling reflects modification of magnetic properties and pinning mechanisms through thermal evolution [27]. Saturation magnetization M_s_ decreases with temperature following Bloch’s law (~3–4% decrease per 100 K near room temperature), proportionally reducing MBN intensity; simultaneously, thermally activated depinning reduces effective pinning strength, producing competing MBN evolution [27]. Consequently, grain size calibration curves demonstrate 5–10% parameter shifts across ±50 K temperature ranges, necessitating temperature-compensated algorithms for field measurements [27].

#### 5.1.12. Machine Learning for Complex Microstructure Analysis

Deep learning architectures—particularly convolutional neural networks (CNNs) operating on multi-parameter MBN spectrograms—achieve superior performance for complex microstructure characterization compared to traditional statistical regression [4,108]. CNN-based approaches achieve grain size prediction accuracies within ±10 μm across 10–200 μm ranges and 90–95% classification accuracy for texture components, substantially outperforming single-parameter threshold approaches [4]. Transfer learning leveraging pretrained networks trained on large MBN datasets enables rapid adaptation to new material systems with minimal calibration data (20–50 samples), addressing practical deployment constraints [108].

### 5.2. Dislocation Density and Crystal Defects

The quantitative characterization of dislocation ensembles through magnetic Barkhausen noise serves as a powerful non-destructive tool for materials science and engineering applications, enabling assessment of work hardening evolution, plastic deformation extent, and structural damage accumulation [35,92]. The fundamental understanding of magnetoelastic coupling with dislocation structures establishes MBN as a versatile tool for monitoring deformation processes and material degradation across diverse industrial contexts aligned with the *Sensors* journal’s emphasis on advanced sensor technologies.

#### 5.2.1. Magnetoelastic Energy and Pinning Force Relationships

Magnetoelastic coupling between domain walls and dislocations originates from strain fields surrounding dislocation cores, which create localized modifications of magnetic anisotropy through magnetoelastic energy contributions [35]. The magnetoelastic energy interaction between a domain wall and a dislocation is expressed as follows:

(5) $$E_{\mathrm{mel}}=-\frac{3}{2}\lambda_{s}\sigma_{\mathrm{local}}{sin}^{2}\theta$$

where λ_s_ denotes saturation magnetostriction, σ_local represents the localized stress field from the dislocation, and θ defines the magnetization orientation relative to the strain axis [35]. Pinning force calculations from micromagnetic simulations reveal that optimal dislocation orientations perpendicular to domain wall propagation direction establish energy barriers ranging from 20 to 100 meV, substantially exceeding point-defect pinning (~5–20 meV) [35]. The orientation-dependent pinning strength exhibits pronounced anisotropy: edge dislocations oriented optimally generate maximum pinning; screw dislocations provide weaker pinning through different magnetoelastic coupling geometry [35].

#### 5.2.2. Collective Behavior of Dislocation Ensembles

Dislocation network interactions produce substantially stronger effective pinning than individual dislocation contributions through collective effects [92]. At moderate dislocation densities (~10^12^ m^−2^), distributed pinning sites create statistically significant resistance to domain wall motion; above ~10^13^ m^−2^, organized dislocation structures (cells, subgrain boundaries) establish preferentially oriented pinning landscapes [92]. Collective pinning strength exhibits superlinear scaling with dislocation density through dimensional considerations: linear pinning site density increases since $\rho_{DW}^{1/2}$, where ρ_DW_ denotes domain wall density; however, effective resistance scales more strongly through collective barrier interactions, producing near-quadratic net scaling [92].

#### 5.2.3. Power-Law Scaling with Dislocation Density (m = 0.3–0.7)

Quantitative MBN–dislocation density relationships follow power-law scaling: ${\mathrm{MBN}}_{\mathrm{RMS}}\propto \rho_{d}^{-m}$, where ρ_d denotes dislocation density and the exponent m typically ranges from 0.3 to 0.7 depending on material class and the deformation pathway [92]. Ferritic steels display exponent values m ≈ 0.5–0.6; dual-phase materials exhibit steeper dependencies (m ≈ 0.6–0.7), reflecting competing grain refinement and phase transformation effects alongside dislocation hardening [92]. The physical basis reflects increased pinning site density; an approximately 1% MBN amplitude decrease per 10^13^ m^−2^ dislocation density increment in ferritic steels enables quantitative dislocation density assessment [92].

#### 5.2.4. Geometrically Necessary Versus Statistically Stored Dislocations

Geometrically necessary dislocations (GNDs)—accumulating at grain boundaries and strain-localized zones to accommodate plastic deformation gradients—exhibit stronger MBN signatures than statistically stored dislocations (SSDs) distributed throughout grain interiors [111]. GND densities at grain boundaries typically exceed bulk SSD concentrations by 5–10×; consequently, high-spatial-resolution MBN measurements reveal grain boundary regions with 20–40% enhanced signal intensity compared to uniform grain interiors [111]. EBSD-based GND mapping, obtained by reconstructing Nye tensor components from local orientation gradients, has been shown to correlate strongly with spatial variations in MBN intensity (coefficients of determination $R^{2}>0.85$), thereby providing independent validation that MBN is sensitive to deformation gradient accumulation and geometrically necessary dislocation density [111].

#### 5.2.5. Dislocation Cell Structures and Subgrain Boundaries

Dislocation cell formation during moderate-to-large plastic deformation produces organized substructures with characteristic dimensions of 0.5–5 μm, separated by high-angle-misorientation subgrain boundaries [28]. MBN’s response to cellular structures exhibits distinctive characteristics: cell interiors (containing relatively low dislocation density ~10^12^ m^−2^) display higher MBN amplitudes; cell walls (containing dense dislocation networks ~10^14^ m^−2^) show suppressed MBN, reflecting enhanced pinning [28]. Two-dimensional MBN mapping across deformed specimens reveals cellular pattern replicas with spatial resolution comparable to cell dimensions [28].

#### 5.2.6. Point Defects and Radiation Damage Effects

Radiation-induced point defects—vacancies and interstitials from neutron/ion bombardment—create pinning sites through magnetic anisotropy modifications, observable through MBN intensity increases following irradiation [76]. Neutron-irradiated reactor pressure vessel steels exhibit progressive MBN amplitude reduction through competing effects: initial defect-induced pinning enhancement (MBN increase) transitions to suppression as defect clusters accumulate and interact with dislocation networks [76]. The dose-dependent MBN evolution correlates with ductile-to-brittle transition temperature shifts, enabling non-destructive fracture toughness assessment [76].

#### 5.2.7. Precipitate–Dislocation Interactions

Complex precipitate–dislocation coupling emerges in age-hardenable systems, where coherent precipitates introduce both direct pinning sites and elastic strain fields interacting with mobile dislocations [112]. Early precipitation stages produce MBN suppression through precipitate-induced pinning; over-aged conditions show MBN recovery as precipitate coarsening reduces effective pinning while dislocation interactions persist [112]. Orowan looping—the mechanism of dislocation motion around non-shearable precipitates—produces characteristic MBN signatures distinct from single-phase dislocation pinning [112].

#### 5.2.8. Temperature and Strain Rate Dependencies

Temperature effects on dislocation–MBN relationships reflect thermal modification of dislocation mobility and pinning mechanism activation [113]. Elevated temperatures enhance thermally activated depinning, reducing effective pinning strength and moderating MBN suppression per unit plastic strain compared to room-temperature deformation [113]. Cryogenic deformation (T < 150 K) produces enhanced MBN suppression through immobilized dislocation populations; dislocations become effectively “frozen” at sub-thermal barriers, establishing stronger pinning [113]. Strain rate dependencies modulate dislocation velocity; higher strain rates reduce dislocation residence times at pinning sites, modifying effective pinning strength and producing strain rate-dependent MBN evolution [113].

#### 5.2.9. Fatigue-Induced Dislocation Structures

Cyclic deformation produces distinctive cyclic slip-induced dislocation band structures, distinguished from monotonic- loading cell formation through ladder-like dislocation arrangements [114]. MBN characteristics during cyclic loading exhibit oscillatory behavior reflecting alternating forward/reverse domain wall displacement cycles; low-cycle-fatigue stages show progressive MBN suppression as cyclic hardening accumulates dislocations, transitioning to relative saturation in high-cycle-fatigue regimes [114].

#### 5.2.10. Work-Hardening Stage Identification

Multi-stage work hardening produces distinctive MBN evolution patterns, enabling microstructural stage identification [28]. Stage I (linear hardening, ε < 2–3%): forest dislocations produce approximately linear MBN suppression; Stage II (parabolic hardening, 3–10% strain): cell formation accelerates suppression through collective pinning; Stage III (saturation, ε > 10%): dynamic recovery/recrystallization onset produces an MBN plateau reflecting competing mechanisms [28].

#### 5.2.11. Complementary Characterization: EBSD and TEM

Electron backscatter diffraction (EBSD) enables quantitative GND density determination through Nye tensor computation from crystallographic misorientation maps; correlations with MBN reveal R^2^ > 0.80 relationships validating MBN sensitivity to deformation gradients [111]. Transmission electron microscopy (TEM) provides direct dislocation imagery confirming density ranges inferred from MBN measurements, establishing consistency between non-destructive and destructive characterization approaches [28].

#### 5.2.12. Applications to Additive Manufacturing and SPD Processes

Severe plastic deformation (SPD) monitoring through MBN enables non-destructive ultra-fine-grained material characterization; MBN signal suppression approaching 80–95% reflects dislocation densities >10^16^ m^−2^ achieved through high-pressure torsion or equal-channel angular extrusion [97]. Additive manufacturing quality control employs MBN for residual stress and near-surface dislocation density assessment, enabling real-time feedback during layer-by-layer material accumulation [30].

### 5.3. Hardness and Strength Relationships

The quantitative correlation between magnetic Barkhausen noise and material hardness represents one of the most industrially significant applications of MBN technology, enabling non-destructive hardness assessment across diverse materials and manufacturing processes [108]. The fundamental physics linking microstructural hardening mechanisms with MBN characteristics establishes a quantitative framework supporting practical sensor deployment for quality assurance and material characterization aligned with the *Sensors* journal’s emphasis on sensor technology applications.

#### 5.3.1. Theoretical Basis: Common Microstructural Dependencies

The fundamental MBN–hardness relationship derives from shared microstructural mechanisms governing both hardening and Barkhausen generation [3]. Hardness reflects material resistance to plastic deformation, fundamentally determined by dislocation density, grain structure, precipitate distribution, and phase composition [3]. MBN intensity directly reflects domain wall mobility, conversely related to pinning site density and pinning strength—parameters also controlling deformation resistance [3]. Consequently, mechanisms producing hardness increases (dislocation multiplication, grain refinement, precipitation, phase transformation) simultaneously suppress MBN through enhanced domain wall pinning, establishing inverse correlations [3].

#### 5.3.2. Logarithmic Correlation: MBN = A − B·log(HV)

Empirical MBN–hardness relationships commonly follow logarithmic functional forms, ${\mathrm{MBN}}_{\mathrm{RMS}}=A-B\times log(\mathrm{HV})$, where A and B denote material-dependent calibration constants, and HV represents Vickers hardness [3,108]. The logarithmic form reflects the fundamental pinning density–MBN scaling; dislocation density scales approximately linearly with hardness, while MBN amplitude exhibits power-law dependence on dislocation density (exponent m ≈ 0.5–0.7), producing combined logarithmic hardness–MBN scaling [3]. Typical correlations demonstrate determination coefficients R^2^ > 0.85–0.90 across industrial hardness ranges (250–700 HV), enabling hardness prediction within ±20–40 HV uncertainty [108].

#### 5.3.3. Precipitation Hardening Versus Work Hardening Mechanisms

Precipitation hardening mechanisms—coherent precipitate formation increasing yield strength through Orowan looping—exhibit distinctive MBN evolution compared to classical work hardening [3]. Precipitation causes localized magnetic anisotropy variations surrounding coherent particles, producing additional pinning sites and MBN suppression; over-aged conditions show MBN recovery as precipitate coarsening reduces effective pinning [3]. Work hardening mechanisms—dislocation forest accumulation—produce more gradual MBN suppression following power-law relationships with strain; logarithmic MBN–hardness relationships remain approximately valid across hardening pathways despite mechanistic differences [3].

#### 5.3.4. Surface Treatment Effects: Induction Hardening, Nitriding, and Carburizing

Induction-hardened components exhibit distinctive MBN characteristics reflecting a case-hardened microstructure overlying a softer base material [115]. The MBN envelope displays characteristic double-peak profiles when case thickness approximates electromagnetic penetration depth (~0.5–3 mm for typical excitation frequencies): a lower-field peak from softer bulk material and a higher-field peak from harder surface case [115]. Peak intensity ratios correlate linearly with case depth up to approximately 1 mm thickness; beyond this threshold, the bulk metal peak becomes negligible [115]. Nitriding processes introduce nitrogen interstitials, creating compound layers with a transformed microstructure; MBN suppression reflects nitride formation and associated dislocation structures, enabling non-destructive nitriding depth assessment [116]. Carburizing processes produce carbon-enriched surface layers with increased martensite fraction; MBN responses reflect competing effects of carbon enrichment (enhanced pinning through increased interstitial content) versus phase transformation (martensite hardening) [116].

#### 5.3.5. Case-Hardened Component Characterization: Double-Peak Profiles

Depth-sensitive MBN measurement via electromagnetic frequency variation reveals near-surface versus bulk material properties through distinct spectral components [115]. Low-frequency excitation (5–50 Hz) penetrates deeply (1–3 mm), capturing bulk properties; high-frequency operation (100–500 kHz) restricts sensitivity to 0.1–0.5 mm surface layers [115]. Case-hardened steels display pronounced two-peak envelope morphology: the first peak (lower magnetic field) reflects a harder case, while the second peak (higher field) reflects a softer core; peak area ratios directly correlate with case depth-to-measurement volume ratios [115].

#### 5.3.6. Grinding Burn Detection and Thermal Damage Assessment

Grinding burn detection exploits MBN sensitivity to thermal damage-induced microstructural changes [84,94]. Burn mechanisms include the following: (1) surface softening—white layer formation and tempering of martensite; (2) subsurface tensile stresses—thermal cycle-induced stress redistribution; (3) hardness variation—pronounced gradients from surface softening through subsurface hardness peaks [84]. MBN measurements reveal characteristic modifications: compressive surface stress typically suppresses MBN; subsurface tensile stresses frequently dominate measurements through electromagnetic depth-weighting, producing net MBN amplification despite surface compression [84]. Multi-parametric analysis combining envelope position, amplitude, and peak count achieves burn detection accuracies exceeding 90% with <1% false-alarm rates [94].

#### 5.3.7. Shot-Peening and Surface Modification Effects

Shot-peening-induced modifications produce characteristic MBN signatures through competing effects: compressive residual stress and work hardening suppress MBN; induced martensitic transformation in metastable austenitic alloys increases the ferromagnetic volume fraction, enhancing MBN [117]. The net MBN response depends on stacking fault energy-dependent deformation mechanisms; low-SFE alloys (AISI 201LN) exhibit pronounced martensitic transformation, producing MBN increases; high-SFE alloys (AISI 304L) show work hardening dominance and net MBN suppression [117].

#### 5.3.8. Temper Embrittlement Detection

Temper embrittlement—drastic toughness loss in certain steels through improper tempering (350–500 °C) or slow cooling from tempering temperature—produces distinctive MBN signatures enabling non-destructive detection [118]. Embrittled specimens exhibit enhanced MBN RMS values reflecting retained martensite and fine dislocation structures; correctly tempered specimens (high-temperature tempering or rapid quenching) show reduced MBN, consistent with reduced dislocation density and fuller austenite reversion [118].

#### 5.3.9. Correlations with Tensile Strength and Yield Stress

Tensile strength–MBN relationships follow similar logarithmic trends to hardness correlations through shared microstructural dependencies; empirical relationships approximate $\sigma_{\mathrm{UTS}}\approx 3.0\times \mathrm{HV},$ enabling indirect strength prediction through MBN-based hardness assessment [3]. Yield stress correlations exhibit weaker scaling through enhanced stress state sensitivity; applied stresses modify anisotropy independently of microstructural pinning, introducing nonlinearities absent in hardness correlations [3].

#### 5.3.10. Hardenability Assessment

Hardenability—material susceptibility to hardening through quenching—exhibits MBN sensitivity through martensite formation, with the extent of formation correlating with carbon content and hardenability [3]. Quantitative hardenability assessment through MBN requires calibration against standard hardenability tests (Jominy end-quench); correlations enable rapid hardenability estimation complementing or substituting for destructive testing [3].

#### 5.3.11. Non-Destructive Hardness Mapping

Two-dimensional hardness mapping through automated scanning systems enables rapid surface hardness distribution characterization [3]. Linear or 2D sensor arrays integrated with robotic positioning systems measure surface hardness at millimeter-scale spatial resolution, generating hardness maps for quality control, process optimization, and component diagnostics [3].

#### 5.3.12. Machine Learning for Improved Prediction Accuracy

Neural network approaches—particularly back-propagation networks processing multiple MBN features—achieve superior hardness prediction compared to single-parameter linear/logarithmic regression [108]. BP-NN models achieve hardness prediction errors of 1.4–3.8%, substantially outperforming single-parameter approaches exhibiting 3–5% errors [108]. Feature importance analysis via mean influence value methods identifies dominant MBN parameters, enabling model optimization [108].

## 6. Advanced Signal Processing Techniques

**Figure 5:**
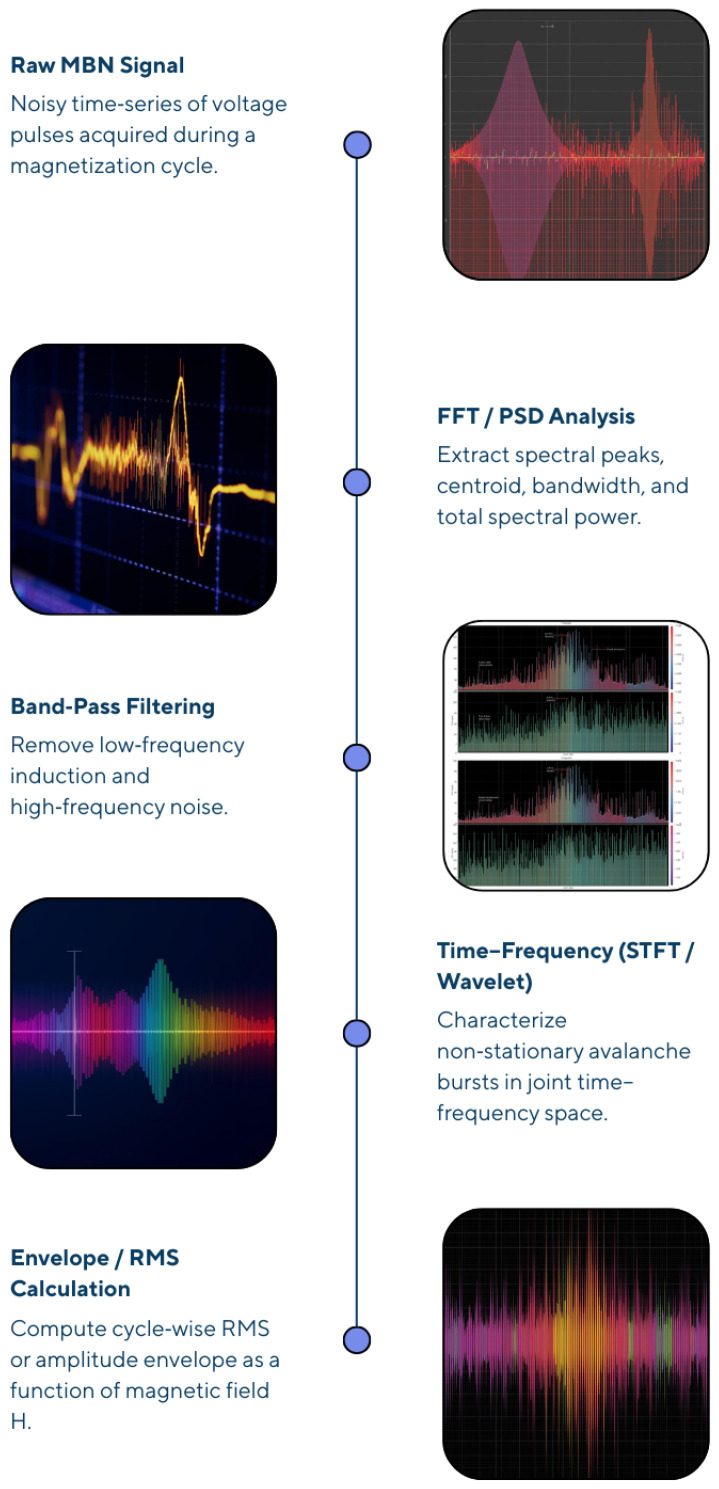
A workflow diagram of signal processing and feature extraction for magnetic Barkhausen noise. The raw noisy time-series is first bandpass-filtered to isolate the Barkhausen frequency band, followed by envelope or RMS evaluation on a cycle basis. Frequency-domain descriptors are obtained via FFT/PSD, while time–frequency methods such as STFT or wavelet analysis capture non-stationary burst behavior. These parameters are assembled into a feature vector that serves as input to machine learning models for material property classification or regression.

The overall data processing pipeline used in modern MBN analysis integrates classical time-domain, frequency-domain, and time–frequency methods with machine learning-based decision algorithms. Figure 5 summarizes this workflow, starting from the raw Barkhausen time-series and progressing through bandpass filtering, envelope/RMS calculation, spectral analysis, and time–frequency decomposition, culminating in the construction of a multi-parameter feature vector for use in classification or regression models. This hierarchical approach enables simultaneous exploitation of amplitude statistics, spectral content, and temporal localization of avalanches, significantly improving sensitivity and robustness in practical material characterization tasks.

### 6.1. Time-Domain Analysis

The time-domain analysis of magnetic Barkhausen noise signals represents a fundamental approach for extracting quantitative material information directly from raw temporal waveforms acquired during magnetization cycles [20,67]. Modern signal processing methodologies systematically extract multiple parameters spanning classical statistical measures through sophisticated temporal feature extraction, establishing comprehensive characterization frameworks supporting industrial quality control and materials research.

#### 6.1.1. RMS Voltage Calculation and Integration Methods

Root-mean-square (RMS) voltage calculation represents the most fundamental MBN parameter, computed as $\mathrm{RMS}=\sqrt{\frac{1}{N}\sum_{i=1}^{N}V_{i}^{2}}$, where N denotes the total sample count and V_i_ represents instantaneous voltage values [24]. RMS integration methods accumulate signal energy over complete magnetization cycles, producing cycle-averaged parameters exhibiting reduced stochastic variability compared to single-pulse measurements [24]. Compensated RMS calculation—subtracting background noise levels determined from high-field regions where Barkhausen activity vanishes—enhances signal-to-noise ratios and improves material sensitivity by 10–20% [67]. Partial RMS computation within specific field windows (±0.5–2.5 kA/m around coercive field) concentrates analysis on peak Barkhausen activity regions, reducing influence from low-sensitivity high-field regions [67].

#### 6.1.2. Peak Amplitude Analysis and Adaptive Thresholding

Peak amplitude detection identifies maximum voltage values within individual Barkhausen pulses, establishing statistical distributions quantifying avalanche sizes [20]. Adaptive thresholding methodologies automatically adjust detection thresholds based on instantaneous noise floor levels, dramatically improving pulse identification in low-signal regimes [24]. Traditional fixed-threshold approaches (typically 20–50 μV) risk missing weak pulses in heavily pinned materials or detecting false-positive noise peaks in high-noise environments; adaptive algorithms dynamically modify thresholds by 2–5× factor ranges, achieving 95%+ pulse detection accuracy across diverse conditions [24].

#### 6.1.3. Pulse Counting with Threshold Optimization

Pulse counting methodologies—enumerating individual Barkhausen events exceeding defined thresholds—generate event count distributions spanning 10^3^–10^6^ pulses per magnetization cycle [3]. Threshold optimization represents a critical preprocessing step; too low thresholds generate false-positive noise counts, while excessively high thresholds miss genuine avalanche events [3]. Optimal thresholds empirically approximate 3–5× background noise RMS values, established through signal detection theory [3]. Event count parameters exhibit strong hardness correlations, ${\mathrm{No}}_{E}^{\mathrm{TOT}}\propto {\mathrm{HV}}^{0.7–1.0}$, enabling quantitative material characterization [3].

#### 6.1.4. Duration Analysis of Barkhausen Events (Pulse Full Width), Rise Time, and Decay Time Measurements

Event duration measurements—quantifying the temporal extent of individual avalanche pulses from initiation to termination—reveal avalanche size–duration relationships underlying power-law scaling laws [20]. Duration distributions exhibit power-law characteristics, $P(T)∼T^{-\alpha }$, with the exponent α ≈ 1.5–2.0 depending on material dimensionality and interaction ranges [20]. Avalanche size–duration correlations follow $\langle S\rangle_{T}∼T^{\gamma }$, where γ denotes the scaling exponent relating average size to duration, approximately γ ≈ 2 for three-dimensional systems with long-range interactions [20].

#### 6.1.5. Rise Time and Decay Time Measurements

Rise time analysis—quantifying temporal slope from avalanche initiation to peak amplitude—characterizes domain wall acceleration kinetics and inertial effects [55]. Rise times typically range from 100 to 500 μs depending on domain wall velocity, damping parameters, and applied field magnitudes [55]. Decay time measurements quantifying pulse return-to-baseline durations reflect damping-dominated magnetization relaxation; decay times generally exceed rise times by 2–5 factors through exponential relaxation dynamics [55]. Rise/decay asymmetry quantified through pulse shape parameters provides material-sensitive information complementary to amplitude-based characterization [55].

#### 6.1.6. Envelope Function Extraction

Envelope function extraction employs analytic signal processing—complex envelope construction from real signals via Hilbert transformation—to recover outer pulse boundaries filtering oscillatory carrier signals [67]. The analytic signal is as follows:

(6) $$z(t)=x(t)+j\hat{x}(t)=|z(t)|e^{j\phi (t)}$$

where $\hat{x}(t)$ denotes the Hilbert transform, enabling the extraction of the instantaneous amplitude envelope ∣z(t)∣ and instantaneous phase ϕ(t) [67]. Envelope parameters—including peak height, width, and integrated area—exhibit superior material sensitivity compared to raw RMS values, with determination coefficients R^2^ > 0.85 for hardness prediction [67].

#### 6.1.7. Statistical Moments: Variance, Skewness, and Kurtosis

The second moment (variance) quantifies signal dispersion around the mean, computed as $\sigma^{2}=\frac{1}{N}\sum_{i=1}^{N}(V_{i}-\overset{-}{V}{)}^{2}$ [20]. The third moment (skewness) characterizes distribution asymmetry as $\gamma_{3}=\frac{1}{N}\sum_{i=1}^{N}\frac{(V_{i}-\overset{-}{V}{)}^{3}}{\sigma^{3}},$ quantifying preferential amplitudes toward high-voltage extreme values [20]. The fourth moment (kurtosis) describes distribution peakedness:

(7) $$\gamma_{4}=\frac{1}{N}\sum_{i=1}^{N}\frac{(V_{i}-\overset{-}{V}{)}^{4}}{\sigma^{4}}-3$$

with positive kurtosis indicating narrower, more-peaked distributions characteristic of constrained pinning regimes [20]. These higher-order moments reveal avalanche dynamics features absent from RMS-based approaches, with skewness exhibiting characteristic peaks identifying relaxation timescales [55].

#### 6.1.8. Autocorrelation Analysis

Autocorrelation functions quantifying signal self-similarity across time lags reveal temporal correlations within avalanche sequences [20]. The autocorrelation is as follows:

(8) $$R(\tau )=\frac{1}{T}\int_{0}^{T}V(t)V(t+\tau )dt$$

This exhibits exponential decay with characteristic correlation timescales reflecting domain wall dynamics [20]. Materials with enhanced pinning produce shorter correlation times through accelerated avalanche termination; weakly pinned systems exhibit longer correlations through extended avalanche durations [20].

#### 6.1.9. Time Delay Analysis for Surface Integrity

Time delay analysis—measuring temporal offset from magnetizing field sinusoid maximum to peak Barkhausen signal occurrence—provides surface integrity characterization sensitive to microstructural variations [67]. The time delay parameter is as follows:

(9) $$\Delta t=t_{\max\mathrm{BN}}-t_{\max\mathrm{current}}$$

This exhibits strong correlation with surface hardness, with $\Delta t\propto {\mathrm{HV}}^{0.5–0.7}$, enabling hardness-based surface condition assessment [67]. Time delay analysis demonstrates superior reliability (correlation coefficient r = 0.989) compared to traditional RMS-based approaches, enabling micro-hardness prediction within ±16 HV uncertainty [67].

#### 6.1.10. Transient and Multi-Scale Analysis

Transient analysis isolates non-stationary signal features occurring during magnetization reversals through continuous wavelet or short-time Fourier transforms [27]. Multi-scale analysis decomposes signals across hierarchical timescales from nanosecond-scale pulse structures through microsecond-scale avalanche envelopes and millisecond-scale magnetization cycle modulations [27]. This hierarchical decomposition enables the separation of competing phenomena: high-frequency components reflect individual domain wall segments; intermediate frequencies capture collective avalanche dynamics; low-frequency modulation reflects overall cycle evolution [27].

### 6.2. Frequency-Domain Methods

The frequency-domain analysis of magnetic Barkhausen noise signals reveals spectral energy distributions across measurement bandwidths, providing material-sensitive information complementary to time-domain approaches [4,68]. Contemporary frequency-domain methodologies systematically extract multiple spectral parameters characterizing Barkhausen signal composition, establishing comprehensive signal processing frameworks supporting industrial material characterization and quality control aligned with the *Sensors* journal’s emphasis on advanced signal processing technologies.

#### 6.2.1. Power Spectral Density (PSD) Calculation

Power spectral density (PSD) computation via fast Fourier transform (FFT) algorithms transforms raw Barkhausen time-domain waveforms into frequency-domain representations, quantifying magnetic energy distribution across excitation bandwidth [68]. The PSD is calculated as $\mathrm{PSD}(f)=\frac{|X(f){|}^{2}}{\Delta f}$, where *X*(*f*) denotes the FFT magnitude and Δf represents frequency resolution [68]. Typical Barkhausen signals spanning a 1–50 kHz measurement bandwidth generate PSD spectra exhibiting 2–3-order-of-magnitude energy variations across frequency ranges, reflecting competing contributions from individual domain wall dynamics (high frequencies) through collective avalanche processes (intermediate frequencies) to magnetization cycle modulation (low frequencies) [68].

#### 6.2.2. Spectral Peak Analysis and Dominant Frequency Identification

Spectral peak analysis—identifying maximum PSD values and corresponding peak frequencies—reveals material-dependent spectral characteristics [68]. Peak frequency typically shifts with material condition: annealed ferritic steels display peak frequencies ~5–15 kHz; hardened steels show peak shifts to 15–25 kHz, reflecting enhanced pinning through increased dislocation density and reduced avalanche duration [68]. Dominant frequency identification enables material state discrimination; single-peak spectra indicate homogeneous microstructures; multi-peak spectra suggest complex heterogeneous structures with competing domain dynamics from distinct microstructural phases [68].

#### 6.2.3. Bandwidth Analysis: 3 dB Bandwidth

Three-decibel (3 dB) bandwidth—defined as a frequency range spanning PSD values ≥ 70.7% of peak magnitude—quantifies spectral concentration [68]. Narrow 3 dB bandwidths (<10 kHz) characterize well-defined domain wall dynamics in uniform microstructures; broad bandwidths (>15 kHz) reflect heterogeneous structures with distributed relaxation timescales [68]. Three-decibel bandwidth correlates inversely with material hardness through dislocation effects, ${\mathrm{BW}}_{3dB}\propto {\mathrm{HV}}^{-0.4–0.5}$, enabling quantitative hardness assessment [68].

#### 6.2.4. Spectral Shape Parameters: Centroid, Spread, and Roll-Off

The spectral centroid—the frequency-weighted center-of-mass of the PSD spectrum—is calculated as $f_{c}=\frac{\sum_{k}f_{k}\times \left|S(f_{k})\right|}{\sum_{k}\left|S(f_{k})\right|}$, where f_k_ denotes frequency and *S*(*f_k_*) represents PSD magnitude [68]. The spectral centroid shifts systematically with material properties; annealed steels exhibit centroids ~8–12 kHz, while hardened steels show shifts to 15–22 kHz, reflecting microstructure-dependent frequency distributions [68]. The spectral spread—the standard deviation around the spectral centroid—quantifies bandwidth breadth and correlates with microstructural homogeneity [68]. Spectral roll-off—the frequency containing 95% cumulative PSD energy—provides characteristic frequency indicators independent of peak magnitude variations [68].

#### 6.2.5. Harmonic Analysis and Nonlinearity Assessment

Harmonic analysis evaluates frequency-domain nonlinearities through systematic examination of integer multiple harmonics (2f, 3f, 4f, etc.) of fundamental magnetizing frequency. Nonlinear ferromagnetic systems (particularly under high excitation amplitudes) generate harmonic distortion exceeding linear theoretical predictions; harmonic amplitudes quantify material nonlinearity through total harmonic distortion (THD). $\mathrm{THD}=\frac{\sqrt{\sum_{n=2}^{\infty }A_{n}^{2}}}{A_{1}}\times 100\%$, where *A_n_* denotes the nth harmonic amplitude. Hardened materials exhibit enhanced harmonic generation through pronounced nonlinear magnetization behavior, reflecting increased domain wall pinning.

#### 6.2.6. Frequency-Dependent Depth Profiling: Skin Effect

Electromagnetic skin effect depth modulation exploits frequency-dependent field penetration. $\delta =\frac{1}{\sqrt{\pi f\mu_{0}\mu_{r}\sigma }}$, where δ denotes penetration depth, f represents frequency, and *μ*_0_, *μ_r_*, *σ* represent permeability and conductivity [84]. Low-frequency excitation (5–50 Hz) penetrates deeply (1–3 mm), emphasizing bulk material properties; high-frequency excitation (100–500 kHz) restricts sensitivity to 0.1–0.5 mm surface layers [84]. Multi-frequency measurement strategies systematically vary analysis frequency, generating depth-resolved property profiles through spectral filtering and signal reconstruction [84].

#### 6.2.7. Filter Bank Decomposition

Filter bank decomposition—partitioning broad-bandwidth signals into multiple frequency sub-bands through parallel bandpass filtering—enables multi-scale analysis, capturing hierarchical microstructural information [27]. Typical implementations employ octave-band or fractional-octave decomposition (½-octave, ⅓-octave) creating logarithmically spaced frequency sub-bands with overlapping frequency coverage [27]. Sub-band analysis reveals that different frequency ranges exhibit distinct material sensitivities: low frequencies (1–10 kHz) relate to bulk microstructural features; mid-frequencies (10–30 kHz) optimally encode stress state; high frequencies (30–100 kHz) enhance surface sensitivity [27].

#### 6.2.8. Spectral Subtraction for Noise Reduction

Spectral subtraction methods—subtracting estimated noise spectral characteristics from signal spectra—improve signal-to-noise ratios while preserving signal characteristics [68]. $S_{\mathrm{signal}}(f)=S_{\mathrm{total}}(f)-\alpha \times S_{\mathrm{noise}}(f)$, where α denotes the subtraction factor (typically 1.0–2.0) determined through high-field regions exhibiting minimal Barkhausen activity, reducing background noise while maintaining target signal integrity [68].

#### 6.2.9. Cepstral Analysis

Cepstral analysis—logarithmic spectral analysis followed by inverse FFT—extracts echo and periodicity structures in the power spectrum, revealing multiplicative components [4]. The cepstral analysis, C(n)=IFFT{log(∣FFT{x(t)}∣)}, reveals periodicities in spectral content corresponding to cascade reflections and repeated structures in magnetization dynamics [4]. Cepstral peaks occurring at frequencies corresponding to repetition periods of dislocation spacings or domain wall distributions enable quantitative defect spacing characterization [4].

#### 6.2.10. Coherence and Cross-Spectral Analysis

Coherence analysis—measuring frequency-domain correlation between paired signals—quantifies correlation strength as a function of frequency. $\gamma_{xy}^{2}(f)=\frac{|G_{xy}(f){|}^{2}}{G_{xx}(f)\times G_{yy}(f)}$, where G denotes cross-spectral density and G_xx_, G_yy_ denote auto-spectral densities [68]. Multi-directional measurements (rolling direction versus transverse direction) reveal texture-dependent coherence patterns; textured materials display high coherence in easy-axis directions (γ^2^ > 0.8) and low coherence in hard-axis orientations (γ^2^ < 0.4), enabling crystallographic orientation characterization [68]. Phase analysis from cross-spectral computation reveals temporal relationships between paired measurements; aligned in-phase signals indicate correlated phenomena, while π-phase-shifted signals suggest competing mechanisms [68].

### 6.3. Time–Frequency Analysis

The time–frequency analysis of magnetic Barkhausen noise addresses fundamental limitations of purely time-domain or frequency-domain approaches by simultaneously capturing the temporal and spectral evolution of non-stationary signals [69,74]. Contemporary time–frequency methodologies systematically decompose complex Barkhausen waveforms into hierarchical representations revealing multi-scale microstructural phenomena, establishing sophisticated signal processing frameworks that support advanced material characterization aligned with the *Sensors* journal’s emphasis on innovative analytical techniques.

#### 6.3.1. Short-Time Fourier Transform (STFT) with Windowing

Short-time Fourier transform (STFT) analysis addresses non-stationarity through windowed Fourier decomposition, computed as $X_{STFT}(t,f)=\int_{-\infty }^{\infty }x(\tau )w(\tau -t)e^{-j2\pi f\tau }d\tau$, where *w*(*τ*) denotes a windowing function constraining the analysis to localized temporal intervals [74]. The resulting spectrogram $\mathrm{SPEC}\left(t,f\right)={\left|X_{STFT}(t,f)\right|}^{2}$ provides two-dimensional time–frequency representations visualizing how spectral energy evolves throughout magnetization cycles [74]. Window function selection—including rectangular, Hamming, Blackman, and Kaiser windows—critically affects resolution trade-offs; Kaiser windows with parameter β = 0.5 provide balanced characteristics applicable across diverse Barkhausen measurement conditions [74]. Window length optimization requires balancing temporal and frequency resolution: small windows (64–128 samples) yield fine temporal localization (~64–128 μs resolution) but reduced frequency specificity; larger windows (512–1024 samples) enhance frequency resolution to 488 Hz but coarsen temporal detail (~2 ms resolution) [74].

#### 6.3.2. Wavelet Transforms: Continuous and Discrete

Continuous wavelet transforms (CWTs) employ basis functions—wavelets—through $C(a,b)=\int_{-\infty }^{\infty }x(t)\psi^{*}(\frac{t-b}{a})\frac{dt}{a}$, where a denotes scale (inversely related to frequency), b represents temporal position, and ψ(t) defines the mother wavelet [27]. CWT provides high redundancy, enabling fine-grained time-scale analysis; Morlet and Mexican-hat wavelets exhibit optimal Barkhausen sensitivity through localized frequency responses matching avalanche timescale distributions [27]. Discrete wavelet transforms (DWTs) employ dyadic (power-of-two) scale decomposition—$a=2^{j}$, b = k·2^j^—producing efficient multi-level decomposition through successive filtering and decimation [27]. DWT generates hierarchical decomposition: approximation coefficients (cA) contain low-frequency coarse features; detail coefficients (cD) capture high-frequency fine features, with J-level decomposition producing $2^{J}$ distinct frequency bands [27].

#### 6.3.3. Multi-Resolution Decomposition

Multi-resolution analysis through successive wavelet decomposition levels reveals hierarchical microstructural contributions [27]. Level-1 decomposition (cA_1_, cD_1_) isolates the lowest frequencies; subsequent levels progressively access higher frequencies: cD_2_ captures the 2–4 kHz band, cD_3_ spans 4–8 kHz, and cD_4_–cD_6_ address 8–16 kHz through 64–128 kHz [27]. Each decomposition level exhibits material-dependent energy distributions: fine-grained materials concentrate energy in higher-level cD coefficients, reflecting numerous small avalanches; coarse-grained materials show energy concentration in lower levels, indicating larger avalanche events [27].

#### 6.3.4. Time–Frequency Feature Extraction

Quantitative spectral features extracted from time–frequency representations enable material characterization without direct spectrogram interpretation [74]. Extracted parameters include the following: (1) temporal features—the RMS, maximum, mean, standard deviation, and variance computed across time axis; (2) spectral features—the spectral centroid, spectral spread, and spectral roll-off computed across frequency axis; (3) statistical moments—skewness and kurtosis characterizing distribution asymmetry and peakedness [74]. Entropy-based measures—Shannon or Renyi entropy computed from normalized time–frequency energy distributions—quantify disorder; low-entropy spectra indicate organized domain dynamics, while high-entropy spectra reflect chaotic or heavily disordered processes [74].

#### 6.3.5. Transient Phenomenon Identification

Transient event detection exploits time–frequency analysis to isolate non-periodic signal components from background continuous activity [27]. Individual Barkhausen avalanche events manifest as time-localized energy concentrations in spectrograms: high-amplitude, narrow-duration events appear as vertical streaks at specific time–frequency coordinates; multi-phase events display temporally extended, frequency-scattered energy distributions [27]. Transient identification enables avalanche-scale analysis independent of cycle-averaged characteristics; rare large-amplitude events detectable through spectral energy thresholding provide direct access to extreme-value statistics fundamental to understanding critical phenomena [27].

#### 6.3.6. Non-Stationary Signal Analysis

Non-stationary characteristics inherent to Barkhausen signals—where spectral composition evolves throughout magnetization cycles—demand time–frequency approaches capturing dynamic evolution [69]. Cycle-dependent spectral evolution exhibits distinctive patterns: low-field regions (H < ±0.5 kA/m) display continuous spectra with relatively uniform energy distribution; near-coercive-field regions (H ≈ ±H_c_) generate narrow spectral bands with concentrated energy, representing domain wall avalanche activity; high-field regions show diminished MBN with low-frequency-dominant characteristics from reversed domain dynamics [69]. The frequency bandwidth expansion from ~5 kHz at low fields to >30 kHz near coercive points quantifies the intensity of irreversible magnetization processes [69].

Time–frequency analysis thus provides indispensable capabilities for sophisticated Barkhausen noise characterization, revealing temporal evolution and multi-scale phenomena inaccessible through conventional single-domain approaches, enabling advanced material diagnosis and sensor-based quality control applications.

### 6.4. Machine Learning and Artificial Intelligence

The integration of machine learning and artificial intelligence with magnetic Barkhausen noise analysis represents a transformative advancement enabling automated feature extraction, nonlinear relationship modeling, and superior prediction accuracy compared to traditional statistical approaches [4,108]. Contemporary AI methodologies systematically leverage labeled training data to learn complex mappings from MBN signals to material properties, establishing sophisticated predictive frameworks supporting industrial quality control and material characterization.

#### 6.4.1. Neural Networks: Feedforward and Recurrent Architectures

Feedforward neural networks—particularly back-propagation networks (BP-NN)—represent foundational architectures mapping MBN features to material properties through cascaded nonlinear transformations [108]. Typical BP-NN configurations employ 50–100 input nodes (corresponding to extracted MBN features), 1–3 hidden layers with 20–100 neurons each utilizing sigmoid or ReLU activation functions, and single- or multiple-output nodes for property prediction [108]. Training via gradient descent with back-propagation iteratively adjusts synaptic weights, minimizing prediction error; mean squared error (MSE) loss functions commonly guide optimization, achieving convergence within 100–500 epochs [108]. BP-NN models achieve hardness prediction accuracies within ±1.4–3.8% error, substantially outperforming single-parameter linear regression (3–5% error) through nonlinear feature combination capabilities [108]. Recurrent neural networks (RNNs)—incorporating temporal dependencies through feedback connections—enable sequential Barkhausen pulse analysis; long short-term memory (LSTM) architectures demonstrate superior performance for time-evolving phenomena including fatigue damage monitoring and real-time deformation tracking [22].

#### 6.4.2. Deep Learning Approaches: CNNs for Time-Series

Convolutional neural networks (CNNs)—traditionally associated with image processing—demonstrate exceptional performance when applied to one-dimensional Barkhausen time-series through temporal convolution operations [4]. CNN architectures for MBN analysis typically comprise (1) input layers receiving raw signal waveforms or time–frequency spectrograms; (2) convolutional layers (3–5 layers) extracting hierarchical features through learnable filter banks; (3) pooling layers reducing dimensionality; (4) fully connected layers integrating extracted features; and (5) output layers providing classification or regression predictions [4]. Raw signal classification without feature engineering—a key CNN advantage—eliminates manual feature design, enabling networks to automatically discover optimal discriminative features directly from data [4]. Grain-oriented silicon steel classification experiments demonstrate 91–99% accuracy using CNN-based approaches versus 80–85% accuracy when using traditional machine learning with hand-crafted features, highlighting CNN’s superiority for complex pattern recognition [4].

#### 6.4.3. Classification and Regression Trees (CARTs)

Decision trees—hierarchical structures recursively partitioning feature space through binary splits maximizing information gain—provide interpretable rule-based classification [119]. CART algorithms construct trees through (1) root node selection, identifying the most discriminative feature; (2) recursive splitting, generating child nodes through threshold optimization; (3) pruning, reducing tree complexity and preventing overfitting [119]. While single trees risk overfitting and instability, their transparent structure enables direct visualization of decision pathways—a significant interpretability advantage over black-box neural networks [119].

#### 6.4.4. Ensemble Learning: Random Forest, AdaBoost, and Gradient Boosting

Random Forest (RF)—ensembles of decision trees trained on bootstrap-sampled subsets—aggregate predictions through majority voting (classification) or averaging (regression), substantially improving robustness compared to single trees [120]. RF implementations for Barkhausen analysis typically employ 100–500 trees, achieving hardness prediction R^2^ > 0.90 with reduced overfitting compared to individual CART models [120]. AdaBoost—adaptive boosting sequentially training weak learners and emphasizing misclassified samples—demonstrates superior performance on imbalanced datasets common in defect detection applications [120]. Gradient Boosting—particularly extreme gradient boosting (XGBoost)—achieves state-of-the-art prediction accuracy through iterative residual minimization; MBN applications report RMSE reductions of 15–25% compared to RF approaches [120].

#### 6.4.5. Support Vector Machines (SVMs) with Kernel Functions

Support vector machines—maximum-margin classifiers defining optimal hyperplanes separating classes in high-dimensional feature spaces—demonstrate robust performance for binary material state discrimination [121]. Kernel functions—including linear, polynomial, radial basis function (RBF), and sigmoid kernels—enable nonlinear decision boundaries through implicit feature space transformations without explicit high-dimensional mapping [121]. RBF kernels $\;K(x_{i},x_{j})=exp(-\gamma ||x_{i}-x_{j}|{|}^{2})$ prove particularly effective for Barkhausen applications, with hyperparameter optimization (regularization C, kernel width γ) critical for performance [121]. Ensemble SVM approaches—training multiple SVM models on data subsets—reduce training complexity while maintaining accuracy; the experimental results demonstrate a 10–100× increase in training speed with <2% accuracy degradation compared to standard LIBSVM implementations [122].

#### 6.4.6. Feature Selection and Dimensionality Reduction

Feature selection methodologies—including mutual information-based selection, recursive feature elimination, and LASSO regularization—identify subsets of maximally informative features, reducing computational complexity and improving generalization [119]. Mutual Information-based Feature Selection with Class-dependent Redundancy (MIFS-CR) achieves superior performance for MBN applications, reducing feature dimensionality from 50–100 to 10–20 features with minimal performance degradation (<3% accuracy loss) [119]. Principal component analysis (PCA)—linear dimensionality reduction projecting data onto orthogonal principal components—reduces 50-dimensional feature spaces to 5–10 principal components capturing > 95% variance, enabling visualization and accelerating training [120].

#### 6.4.7. Training Data Requirements and Augmentation

Training dataset size critically determines model performance; empirical studies indicate minimum requirements of 200–500 samples for simple neural networks and 1000–5000 samples for deep CNNs, achieving robust generalization [4]. Data augmentation strategies—including synthetic data generation through Gaussian noise injection, time-shifting, and amplitude scaling—artificially expand training datasets by 5–10×, enabling improved model robustness without additional physical measurements [21]. Transfer learning—leveraging pretrained models from related tasks—reduces training data requirements to 50–100 samples for fine-tuning applications [108].

#### 6.4.8. Cross-Validation and Performance Metrics

K-fold cross-validation—partitioning datasets into K subsets (typically K = 5–10), iteratively training on K-1 folds, and validating on the remaining fold—provides unbiased performance estimates [120]. Performance metrics including accuracy, precision, recall, F1-score (classification), root-mean-square error (RMSE), mean absolute error (MAE), and determination coefficient (R^2^) (regression) quantify model capabilities [120]. Stratified cross-validation—preserving class proportions—proves essential for imbalanced datasets common in defect detection [120].

#### 6.4.9. Decoupling Concurrent Influences on MBN

In practical components, Barkhausen noise responds simultaneously to residual stress, hardness, grain size, dislocation density, phase composition, and surface integrity, which raises the critical question of how to isolate the individual contribution of each factor. Controlled calibration experiments address this issue by varying only one parameter at a time—such as uniaxial stress on stress-relieved specimens, or tempering heat treatments at a fixed stress state—while keeping all other variables nominally constant, thereby providing reference surfaces for subsequent inversion. Frequency-dependent excitation and multi-parameter measurements further support factor separation: by exploiting different effective sampling depths and combining MBN with incremental permeability or ultrasonic parameters, several studies have demonstrated that stress and microstructural effects can be distinguished with multivariate regression and feature selection techniques. More recently, random forest and support vector machine models trained on carefully designed factorial datasets have been used to estimate stress and hardness simultaneously from the same MBN feature vector, while sensitivity analysis and mutual information-based feature ranking identify which features are predominantly associated with each physical variable. Although perfect decoupling is rarely achievable in service conditions, these combined strategies—factorial experimental design, multi-frequency and multimodal sensing, and multivariate/AI-based inversion—provide a practical route to quantifying the dominant influence of individual factors for given material classes.

#### 6.4.10. Interpretability Versus Accuracy Trade-Offs

Model interpretability—understanding decision-making processes—conflicts with accuracy optimization in complex models [123]. Decision trees and linear models offer transparent decision pathways enabling engineering validation but achieve moderate accuracy; deep neural networks and ensemble methods attain superior accuracy while functioning as black boxes limiting interpretability [123]. Grad-CAM visualization—gradient-weighted class activation mapping—enables post hoc CNN interpretation through saliency map generation, identifying signal regions influencing predictions, partially addressing interpretability limitations [123].

## 7. Industrial Applications

**Figure 6:**
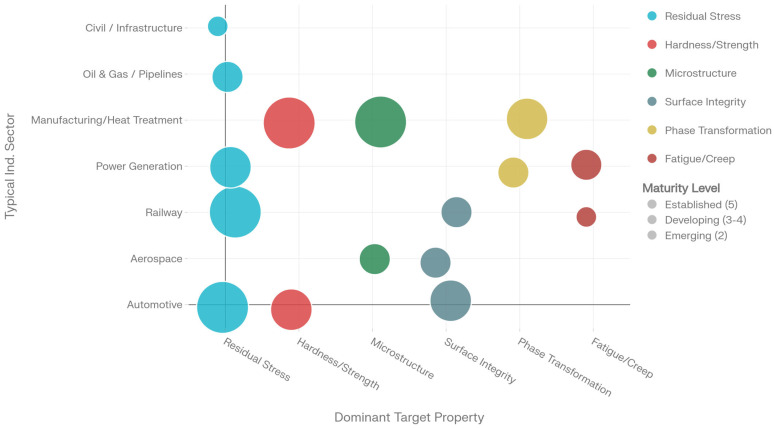
Application map of magnetic Barkhausen noise (MBN) linking dominant target properties to major industrial sectors. The horizontal axis groups the principal material properties assessed by MBN—residual stress, hardness/strength, microstructure, surface integrity, phase transformation behavior, and fatigue/creep damage—while the vertical axis lists representative sectors. Bubble size indicates the relative maturity of each application domain, with larger bubbles corresponding to well-established use cases.

The versatility of MBN as an industrial NDT tool is best appreciated when viewed across both target properties and application domains. Figure 6 presents an application map in which bubble positions represent combinations of dominant material properties assessed by MBN and the industrial sectors where these measurements are routinely performed. Established applications, indicated by larger bubbles, include residual stress and hardness evaluation in automotive powertrain components, railway rails and wheels, and induction-hardened machine parts, as well as microstructure and phase transformation monitoring in heat treatment lines. Emerging areas, denoted by smaller bubbles, involve fatigue and creep damage assessment in power generation components and residual stress mapping in civil infrastructure and pipeline systems, where MBN is increasingly investigated as part of multimodal NDT strategies.

### 7.1. Manufacturing Quality Control

#### 7.1.1. Industrial Applications: Manufacturing Quality Control

The implementation of magnetic Barkhausen noise in manufacturing quality control represents a transformative advancement enabling real-time material characterization and process optimization across diverse industrial applications [24,80]. The integration of MBN-based monitoring systems with automated production workflows supports Industry 4.0 paradigms, establishing predictive quality frameworks and autonomous decision-making capabilities aligned with the *Sensors* journal’s emphasis on advanced sensor technology and industrial applications.

#### 7.1.2. Real-Time Monitoring During Heat Treatment Processes

In situ heat treatment monitoring employs MBN to track microstructural evolution during critical thermal processes including hardening, tempering, carburizing, and nitriding [124]. Non-destructive real-time measurements during process execution enable immediate detection of anomalies—insufficient heating, excessive cooling rates, and compositional deviations—triggering corrective action before component acceptance [124]. Nitriding depth control through MBN signals acquired from active electrodes during diffusion processes provides direct assessment of nitrogen layer development, eliminating uncertainty from indirect gas composition measurements [124]. Laboratory implementations demonstrate MBN’s ability to differentiate nitrided layer thickness variations of <10 μm, enabling process parameter optimization and ensuring uniform depth specifications across batches [124].

#### 7.1.3. Grinding Process Assessment and Burn Detection

Grinding burn detection represents the most mature and industrially established MBN application, with deployment spanning aerospace, automotive, and bearing manufacturing sectors [94]. Grinding-induced thermal damage—manifesting as surface softening, tensile stresses, or white layer formation—produces characteristic MBN signature changes directly correlating with thermal severity: moderate heat generates surface compression and subsurface tension producing complex envelope profiles; excessive heat creates over-tempered structures with drastically reduced MBN amplitudes [94]. Frequency-dependent burn sensitivity exploits the electromagnetic skin effect: low-frequency MBN (5–50 kHz) detects subsurface thermal effects; high-frequency analysis (100–500 kHz) emphasizes near-surface signatures, enabling depth-selective damage characterization [93]. Field implementations report > 95% grinding burn detection rates with <1% false-alarm rates, achieving 100% production coverage at cycle times compatible with manufacturing throughput [80].

#### 7.1.4. Surface Integrity Evaluation After Machining

Post-machining surface characterization through MBN quantifies near-surface microstructural and stress modifications from tool–workpiece interactions, enabling discriminatory assessment of machining quality [116]. Hard milling operations generate distinctive MBN signatures reflecting white layer formation (reheated martensite) and heat-affected zones: characteristic asymmetrical Barkhausen envelopes distinguish hard-milled surfaces from conventionally heat-treated materials despite similar overall hardness, enabling process traceability [116]. Surface roughness and integrity parameters derived from MBN measurements demonstrate superior sensitivity to machining-induced damage compared to traditional surface profilometry, enabling root-cause identification of process deviations [116].

#### 7.1.5. Shot-Peening and Surface Treatment Verification

Shot-peening quality assessment employs MBN to non-destructively verify compressive residual stress development essential for fatigue performance enhancement [125]. Barkhausen noise signal amplitude reduction during shot-peening directly correlates with induced compressive stresses through magnetoelastic coupling: baseline soft material MBN decreases as peening accumulates stress, enabling real-time coverage and intensity verification without sample removal [125]. Automated peening system feedback integrating MBN measurements enables in-process parameter adjustment—ball velocity, dwell time, and coverage patterns—to achieve uniform residual stress distributions, substantially improving fatigue life predictions compared to traditional Almen strip calibration [125]. Modern systems combining coverage imaging with Barkhausen measurements achieve comprehensive process documentation meeting aerospace and automotive quality requirements [80].

#### 7.1.6. Statistical Process Control Implementation

Statistical process control (SPC) methodologies employing MBN establish quantitative quality boundaries, supporting autonomous production decisions without human inspection [24]. Control charts tracking MBN parameters over sequential components reveal process drift, enabling intervention before systematic out-of-specification production occurs [24]. Capability index calculations (C_p_, C_pk_) quantifying process centering and spread demonstrate whether MBN-based systems reliably distinguish conforming from non-conforming parts; typical implementations achieve C_pk_ > 1.33, supporting zero-defect production objectives [24].

#### 7.1.7. Inline Inspection Systems

Integrated inline Barkhausen systems—installed directly in production lines during component transit—enable 100% inspection compatibility with modern manufacturing throughput [80]. Automated sensor positioning (robotic arms, gantry systems) rapidly acquires measurements on component geometries ranging from flat surfaces to complex curved features; measurement cycle times of 2–5 s per component support production rates of 600–1800 parts/h [80]. Real-time data transmission to MESs (manufacturing execution systems) and ERP (enterprise resource planning) systems enables immediate quality feedback, diverting out-of-specification components for rework while releasing conforming parts to downstream operations [80].

#### 7.1.8. Process Optimization and Feedback Control

Closed-loop process optimization leverages MBN measurements as control variables, adjusting manufacturing parameters to maintain optimal process windows [126]. Grinding parameter optimization combining process modeling with MBN measurements enables systematic selection of wheel speeds, feed rates, and depths of cut, maximizing productivity while maintaining grinding burn-free surfaces [126]. Polynomial regression models fitted to MBN, surface roughness, and cutting power data enable the development of grinding productivity optimization functions, maximizing material removal rates subject to surface integrity constraints [126]. Industrial implementations report productivity improvements of 15–25% through optimized parameter selection while maintaining 100% conformance with quality specifications [126].

### 7.2. Structural Health Monitoring

#### 7.2.1. Railway Infrastructure and Beyond

The application of magnetic Barkhausen noise to structural health monitoring represents a critical advancement enabling non-destructive assessment of critical transportation infrastructure and industrial pressure systems [83,88]. The integration of MBN methodologies into operational monitoring frameworks addresses the pressing need for continuous, quantitative condition assessment of assets whose failure poses severe safety and economic consequences.

##### Railway Infrastructure Applications: Rail Stress and Residual Stress Monitoring

Residual stress evaluation in railway rails represents the most established MBN application in transportation infrastructure, with field deployments spanning multiple global rail networks [88]. Residual stresses developed during rail fabrication, heat treatment, and subsequent wheel-contact loading directly affect fatigue characteristics and crack propagation; quantitative residual stress assessment enables predictive maintenance scheduling and fracture risk mitigation [88]. The magnetic Barkhausen noise method achieves a stress measurement accuracy of ±15 MPa—adequate for structural significance discrimination—through careful magnetization protocol optimization and directional sensitivity exploitation [88]. Applied tensile loads produce linear MBN amplitude increases when the measurement direction aligns with load orientation; perpendicular measurement orientations exhibit negligible signal variation, enabling multiaxial stress state determination through tri-directional measurements [88].

Continuous welded rail (CWR) temperature stress evaluation through combined MBN–metal magnetic memory (MMM) techniques achieves stress detection accuracies of 71.43–82.76% across operating stress ranges (−30 to +90 MPa), enabling rapid on-site assessment supporting dynamic track geometry management [127,128]. The combined MBN-MMM methodology substantially outperforms single-technique approaches through complementary information fusion, with particular effectiveness for early damage detection before catastrophic failure [127].

##### Ultrasonic–Barkhausen Combined Measurements

Hybrid measurement frameworks combining MBN with complementary ultrasonic techniques substantially enhance diagnostic discrimination for complex damage scenarios [63]. Ultrasonic methods probe bulk elastic properties and detect crack-like defects, while MBN characterizes microstructural state and near-surface stresses—orthogonal information providing comprehensive structural assessment [63]. Integration of these modalities enables automated anomaly detection supporting unattended monitoring systems essential for remote rail corridors.

##### Prestressed Concrete Strand Assessment

Magnetoelastic methods exploiting stress-dependent magnetic permeability modifications enable non-destructive tendon force monitoring in prestressed concrete structures [90]. Embedded elastomagnetic sensors integrated within prestressed strands detect tension variations through permeability changes, achieving ±5% force estimation accuracy over multi-year deployments with battery-free operation through energy harvesting [90]. This capability directly addresses the critical infrastructure challenge of continuous prestress monitoring in bridges and parking structures, where sudden prestress loss indicates anchorage failure or tendon fracture necessitating emergency intervention [90].

##### Switch Point and Crossing Frog Inspection

Switching mechanism inspection through MBN characterizes residual stress distributions in complex wheel-contact regions, identifying stress concentration zones susceptible to fatigue crack initiation. The high-spatial-resolution MBN sensor configurations enable localized measurements within constrained geometries, supporting defect mapping and predictive maintenance scheduling for safety-critical switching infrastructure.

#### 7.2.2. Bridge and Structural Component Monitoring

Structural steel bridge monitoring employs MBN for residual stress profiling in welded joints and fastened connections, identifying regions with elevated tensile stress or thermal damage promoting fatigue [83]. Automated scanning systems mounted on robotic platforms enable comprehensive bridge deck assessment during routine inspections, with measurement cycles compatible with normal traffic operations.

#### 7.2.3. High-Speed Rail and Heavy-Haul Operations

Accelerated wear assessment in high-speed and heavy-haul rail operations through MBN detects incipient damage preceding visible wear, enabling proactive wheel and rail replacement [88]. The directional MBN sensitivity to asymmetric wheel loads characteristic of curved track sections provides enhanced damage detection compared to single-directional measurement approaches.

#### 7.2.4. Urban Transit Systems

Rapid transit rail monitoring systems integrate MBN measurements with condition-based maintenance protocols, optimizing replacement timing and reducing service disruptions through predictive scheduling [127]. The non-contact solenoid-type MBN sensors enable measurements through accumulated dirt and corrosion products without cleaning requirements, practical for urban subway applications with restricted maintenance windows.

#### 7.2.5. Pressure Vessels and Pipeline Inspection

Pressure boundary integrity assessment in ferromagnetic pressure vessels exploits MBN sensitivity to microstructural degradation and residual stress redistribution from service-induced creep, fatigue, or stress relaxation [127,128]. Periodic MBN surveys enable early detection of material degradation before operational stress limits approach fracture thresholds, supporting extended equipment service life while maintaining safety margins.

### 7.3. In-Service Inspection and Condition Monitoring

#### 7.3.1. In-Service Inspection and Condition Monitoring

The in-service inspection and condition monitoring of ferromagnetic structures and components through magnetic Barkhausen noise represents a transformative advancement enabling continuous assessment of material degradation and remaining useful life [96,114]. Modern condition-based maintenance paradigms leverage real-time MBN measurements to optimize inspection scheduling and replacement timing, delivering substantial economic benefits through predictive rather than reactive maintenance strategies on practical sensor technology applications.

#### 7.3.2. Fatigue Damage Detection and Remaining Life Assessment

Cyclic loading responses demonstrate distinctive MBN evolution patterns quantitatively tracking fatigue damage progression across service lifetime stages [114]. Early-stage fatigue (N < 0.1 N_f_, where N_f_ denotes cycles to failure) exhibits MBN characteristics reflecting cyclic hardening and dislocation structure refinement; temporal MBN profiles display oscillatory behavior synchronized with loading cycles [96]. Mid-stage fatigue (0.1 N_f_ < N < 0.7 N_f_) maintains relatively stable MBN as cyclic steady-state microstructure persists; subtle secondary effects from shallow microcrack initiation remain below detection thresholds of traditional single-parameter measurements [96]. Late-stage fatigue (N > 0.7 N_f_) reveals sharp MBN signal reductions corresponding to macroscopic crack propagation, with 20–60% amplitude suppression preceding final fracture, enabling quantitative damage assessment before critical failure.

Remaining useful life (RUL) prediction models leverage fatigue stage identification through time-derivative analysis of MBN evolution, achieving 80–95% accuracy in predicting remaining cycles before critical threshold values [114]. Machine learning approaches processing multi-parameter MBN features outperform traditional single-parameter thresholding through exploitation of complex nonlinear relationships between damage progression and signal characteristics [114]. The temporal evolution of spectral parameters—particularly frequency centroid shift and bandwidth expansion—provides supplementary RUL indicators complementing amplitude-based approaches and improving prediction robustness across diverse material conditions [114].

#### 7.3.3. Corrosion Monitoring and Early Detection

Corrosion-induced microstructural degradation produces characteristic MBN signatures enabling early detection before penetration corrosion breaches structural boundaries [76]. Surface corrosion pit initiation creates localized plasticity and dislocation multiplication within pit-adjacent material, detectable through MBN amplitude increases preceding visible pit enlargement; this early-warning capability enables corrective action before critical loss of thickness occurs [76]. Subsurface corrosion development—particularly dangerous in pressure vessels and pipelines where external surfaces remain protected while internal corrosion progresses—produces microstructural changes detectable through frequency-dependent MBN measurements exploiting electromagnetic depth-weighting [76].

Automated corrosion monitoring platforms integrated with wireless transmission enable continuous assessment across geographically dispersed assets without requiring personnel access to hazardous or remote locations [26]. Long-duration passive operation through energy harvesting from excitation field oscillations eliminates battery replacement burdens during extended monitoring campaigns [26].

#### 7.3.4. Creep Damage Assessment in High-Temperature Components

Creep-induced microstructural evolution in high-temperature service produces characteristic MBN signatures tracking cavitation, precipitate coarsening, and dislocation substructure development [28]. Thermal power generation equipment, including boiler tubes and turbine casings, experiences progressive creep damage, reducing load-bearing capacity; periodic MBN assessments enable quantitative damage staging, supporting predictive replacement scheduling [28]. Comparative measurements at operating temperature and after controlled cooling through elevated-temperature MBN variants reveal phase stability and residual stress redistribution attendant to creep deformation [28].

#### 7.3.5. Condition-Based Maintenance Strategies

Condition-based maintenance (CBM) paradigms employ MBN measurements as trigger variables, initiating maintenance interventions when detected parameters exceed established threshold values [24]. Unlike fixed-interval or run-to-failure strategies, CBM optimization minimizes total ownership costs through balanced consideration of maintenance labor expense, component replacement cost, and catastrophic failure consequences [24]. Economic analysis demonstrates 20–40% lifetime cost reductions through optimized CBM thresholds compared to conventional fixed-interval maintenance, with particularly pronounced benefits for high-value components exhibiting substantial variability in degradation rates [24].

#### 7.3.6. Automated Inspection Platforms

Robotic inspection systems combining MBN sensors with automated positioning establish standardized measurement protocols across large structural assets, reducing measurement variability from operator techniques [79]. Integrated data management systems archive complete measurement histories, supporting trend analysis and anomaly detection through statistical process control methodologies [79]. Real-time decision algorithms directing non-conforming components toward enhanced scrutiny while releasing conforming components to service optimize manufacturing throughput while maintaining quality objectives [79].

#### 7.3.7. Wireless Monitoring Networks

Distributed wireless sensor networks combining miniaturized MBN probes with low-power communication protocols enable continuous structural assessment across extended geographic areas without dedicated measurement infrastructure [26]. Cloud integration architectures support longitudinal trend analysis through rolling-window anomaly detection algorithms, identifying material property evolution, indicating advancing degradation or stress concentration development [26]. Autonomous decision-making capabilities trigger maintenance notifications when measured parameters deviate from established baselines via thresholds, indicating imminent failure risk [26].

#### 7.3.8. Economic Benefits and Cost–Benefit Analyses

Economic justification for MBN implementation relies on quantifiable cost avoidance through extended component service life and the prevention of catastrophic failures, imposing severe direct and indirect consequences [24]. Typical cost–benefit analyses demonstrate payback periods of 1–3 years for high-value industrial assets through reduced inspection labor, optimized maintenance scheduling, and extended component life [24]. Secondary economic benefits including improved safety (reduced failure-induced accidents), enhanced operational availability (predictive scheduling minimizes production interruptions), and superior asset utilization rates collectively support strong financial cases for MBN deployment [24].

## 8. Challenges and Future Directions

The magnetic Barkhausen noise methodology, despite remarkable advances in sensor technology, signal processing, and practical applications over the past decade, confronts several interconnected technical and implementation challenges that must be addressed to realize its full potential for advanced material characterization and structural health monitoring [24,27]. Strategic resolution of these challenges, coupled with emerging research directions, will position MBN technology at the forefront of next-generation sensor systems.

Measurement standardization remains a critical impediment to universal industrial adoption of MBN technology, with the absence of internationally agreed calibration protocols producing interlaboratory variations exceeding ±15% despite nominally identical specimen preparation. Round-robin studies comparing equipment from different manufacturers reveal substantial discrepancies in measured parameters, reflecting sensor design differences, excitation waveform variations, signal conditioning algorithms, and software implementations. Establishing comprehensive international standards—encompassing sensor calibration methodologies, magnetization protocols, signal processing specifications, and quantitative interpretation criteria—represents an essential prerequisite for widespread industrial deployment.

Advanced material characterization including additively manufactured components, high-entropy alloys, compositionally complex steels, and ceramic–metal composites remains comparatively underdeveloped relative to conventional steels. The physical properties and microstructural characteristics of these emerging material systems frequently exhibit behaviors outside the empirical calibration ranges of existing MBN models, necessitating fundamental research establishing quantitative MBN relationships for novel material classes. Particularly challenging scenarios—including multi-phase materials with competing ferromagnetic and non-magnetic constituents, stronglyvtextured microstructures exhibiting pronounced magnetic anisotropy, and materials with extreme hardness or low saturation magnetization—require dedicated investigation and algorithm development.

Field deployment challenges including temperature fluctuations (±50 K variations producing 5–10% parameter shifts), electromagnetic interference from industrial equipment, humidity exposure, and mechanical vibration necessitate enhanced environmental compensation and sensor hardening. Current temperature-dependent correction algorithms, while adequate for ±5 °C variations under laboratory conditions, require substantial refinement for field applications spanning ±50 K temperature ranges. Ferrite core magnetic properties demonstrate pronounced temperature sensitivity; advanced core materials including high-entropy alloys offer superior stability but require integrated thermal management within sensor packages.

Model generalization across material systems, processing histories, and operational conditions represents a fundamental challenge limiting practical deployment of machine learning approaches. Deep learning models trained on laboratory-controlled datasets frequently exhibit substantial accuracy degradation when applied to field measurements containing unexpected signal characteristics or material states absent from training data. Physics-informed neural networks—incorporating fundamental domain knowledge constraints—offer promising solutions through improved generalization, but require substantial algorithmic development before practical implementation.

Extended frequency operation toward gigahertz ranges would enable investigation of fundamental domain wall dynamics and enhanced depth sensitivity for thick-section components, but requires novel instrumentation architectures exceeding current commercial implementations. High-temperature measurements beyond 300 °C necessitate specialized sensor designs employing advanced materials and cryogenic-free cooling, currently limiting monitoring of turbine components and furnace equipment.

Autonomous sensor systems combining miniaturized MBN probes with onboard data processing, wireless communication, and cloud connectivity remain underdeveloped compared to conventional sensors. Integration challenges including standardized data formats, cyber-security protocols, and autonomous decision-making algorithms require collaborative industry-academia initiatives.

Integrated measurement frameworks combining MBN with complementary non-destructive techniques—ultrasonic, eddy current, acoustic emission, and thermal imaging—show substantially enhanced diagnostic capability for complex damage scenarios, but require the development of unified data fusion architectures and decision algorithms. The complementary sensitivities of diverse techniques provide orthogonal information enabling discrimination between competing damage mechanisms currently impossible with single-modality approaches.

Emerging research frontiers include the following: (1) fundamental physics investigations—exploring quantum tunneling effects in low-temperature phase transformations and relativistic corrections in extreme magnetic field conditions; (2) advanced material development—engineered ferromagnetic nanocomposites optimizing sensor performance through controlled magnetic properties; (3) artificial intelligence advancement—physics-informed machine learning incorporating domain knowledge for improved generalization; (4) autonomous systems integration—miniaturized wireless sensors with energy harvesting and edge computing capabilities supporting distributed structural health monitoring networks; (5) standardization initiatives—international collaborative efforts establishing measurement protocols, calibration standards, and interpretation frameworks enabling universal industrial adoption. Specifically, the development of universal calibration blocks and round-robin testing protocols is essential to quantify and minimize inter-laboratory variability, ensuring that MBN data are comparable across different instruments and operators.

A persistent challenge is the strong coupling between stress, microstructure, and surface condition in practical components, which complicates unique inversion of MBN parameters. Future work should therefore prioritize statistically designed calibration campaigns and multimodal measurements (e.g., MBN combined with incremental permeability or ultrasonics) to enable more robust factor decoupling across different steel grades and processing histories.

Despite significant progress, several fundamental questions remain unresolved, representing critical opportunities for future research:

- Decoupling Multi-Source Signals: Isolating the individual contributions of simultaneous microstructural changes (e.g., grain refinement vs. precipitation) and stress states remains a complex inverse problem, often requiring multi-frequency or multi-parameter approaches that are not yet fully standardized.
- Quantitative Model Universality: While stochastic models describe avalanche statistics well, a universal predictive model linking specific alloy compositions directly to MBN signatures without extensive empirical calibration is still lacking.
- High-Speed Rail and Extreme Environments: The behavior of MBN signals under the extreme dynamic loads and high-speed conditions relevant to modern railway infrastructure requires further validation, particularly regarding the long-term stability of sensors in harsh operating environments.

## 9. Conclusions

Tise comprehensive review of magnetic Barkhausen noise sensor technology and applications demonstrates the remarkable evolution of this technique from a fundamental physical discovery into a sophisticated, multifaceted tool essential for contemporary materials characterization and structural health monitoring. Over the past decade, transformative advances spanning theoretical understanding, instrumentation innovation, signal processing methodologies, and industrial implementation have positioned MBN at the forefront of non-destructive testing technologies addressing critical challenges in manufacturing, infrastructure maintenance, and condition-based diagnostics.

The theoretical foundations rooted in statistical mechanics and domain wall physics provide quantitative frameworks enabling the interpretation of Barkhausen signals in terms of microstructural features and mechanical states. The integration of Jiles–Atherton hysteresis models with mean-field theory and experimental validation of power-law scaling relationships establish MBN as a model system for investigating critical phenomena with practical material characterization applications. This synergistic combination of fundamental physics with engineering relevance positions MBN uniquely among non-destructive testing methodologies.

Instrumentation advances including miniaturized sensors, multi-parameter measurement systems, high-entropy alloy cores, and automated scanning platforms enable deployment across diverse industrial applications previously inaccessible to MBN technology. The emergence of non-contact solenoid-type sensors and wireless monitoring networks extends applicability to harsh operating environments and distributed asset monitoring, supporting the transition toward Industry 4.0 autonomous systems and digital manufacturing paradigms.

Signal processing methodologies spanning time-domain, frequency-domain, time–frequency, and machine learning approaches collectively enable extraction of comprehensive material information from raw Barkhausen signals. Deep learning architectures, particularly convolutional neural networks applied to spectrograms and LSTM networks capturing temporal dependencies, demonstrate superior performance compared to traditional statistical approaches while reducing operator expertise requirements. Physics-informed neural networks incorporating domain knowledge constraints offer promising solutions for improved generalization across material systems and operational conditions.

Key material property correlations established through systematic research demonstrate quantitative relationships between MBN parameters and grain size, hardness, dislocation density, residual stress, and phase composition. These correlations, combined with stress sensitivity through magnetoelastic coupling, enable comprehensive material diagnosis supporting quality assurance, predictive maintenance, and structural integrity assessment across aerospace, railway, manufacturing, and pressure system applications.

Challenges requiring strategic attention include the standardization of measurement protocols enabling universal industrial adoption, the characterization of emerging materials including additively manufactured components and high-entropy alloys, the development of robust machine learning approaches ensuring field deployment reliability, and the integration of autonomous MBN systems within digital manufacturing ecosystems. High-frequency extension toward gigahertz ranges and high-temperature operation beyond 300 °C represent technical frontiers demanding novel instrumentation architectures.

Future directions encompassing multimodal sensor fusion, physics-informed artificial intelligence advancement, autonomous wireless sensor networks with cloud integration, and international standardization initiatives promise substantial enhancements to diagnostic capability and industrial adoption rates. The integration of MBN technology with complementary non-destructive techniques creates opportunities for comprehensive damage discrimination in complex materials and loading scenarios currently beyond single-modality capabilities.

Magnetic Barkhausen noise technology has evolved into a mature, versatile sensor methodology combining rigorous physical foundations with proven practical utility across diverse industrial applications. Continued advancement through standardization, machine learning integration, and autonomous system development will establish MBN as an indispensable technology for twenty-first-century material characterization and structural health monitoring, supporting safe, sustainable, and economically efficient engineering practice across critical infrastructure and manufacturing sectors.

## Figures and Tables

**Table 1:** Qualitative comparison between magnetic Barkhausen noise (MBN) and classical magnetic non-destructive testing (NDT) techniques, including hysteresis/coercivity measurements, magnetic flux leakage (MFL), eddy current (EC), and metal magnetic memory (MMM). The table highlights differences in measured quantities, microstructural and stress sensitivity, penetration depth, defect detectability, calibration demands, and data processing complexity, as synthesized from established reviews of magnetic NDT methods.

Aspect	Magnetic Barkhausen Noise (MBN)	Classical Magnetic NDT (Hysteresis/Coercivity, MFL, EC, MMM)
**Primary measured quantity**	High-frequency voltage pulses from discontinuous domain wall jumps under alternating magnetization.	Hysteresis: bulk magnetization vs. field loop; MFL: leakage flux above defects; EC: coil impedance change due to eddy currents; MMM: self-magnetization field anomalies in weak ambient field.
**Sensitivity to microstructure**	Very high sensitivity to grain size, dislocation density, precipitation state, and phase fractions through domain wall pinning landscape.	Hysteresis/coercivity: sensitive to bulk microstructure but with lower spatial resolution; MFL and EC primarily sensitive to geometric defects and conductivity/permeability; microstructural effects are secondary.
**Sensitivity to stress/residual stress**	Highly sensitive to elastic and plastic stress via magnetoelastic coupling; widely used for residual stress evaluation in steels.	Hysteresis-based stress measurements possible but require bulky magnetizing frames; MFL and EC show weaker, often indirect stress sensitivity; MMM can detect stress concentration but is mainly qualitative.
**Depth of penetration**	Limited to near-surface region controlled by skin depth (typically tens to a few hundred micrometers at 10–200 kHz).	Hysteresis: bulk; MFL: surface and subsurface up to several mm; EC: depth controlled by frequency (from μm to mm); MMM: near surface but with larger sensing region along component.
**Defect detection capability**	Indirect for cracks/voids; strongest for changes in hardness, stress, and microstructure rather than isolated sharp defects.	MFL and EC are excellent for surface/subsurface cracks, corrosion, pitting, and wall loss; hysteresis and MMM better for global condition and stress concentration zones.
**Spatial resolution**	High local resolution (sub-millimeter) achievable with small probes and scanning; suitable for mapping stress/microstructure gradients.	Hysteresis: low spatial resolution (bulk); MFL and EC: mm scale depending on probe; MMM: typically coarse, used for line scanning of long components.
**Measurement speed and portability**	Fast, point-wise measurements (ms per point) with compact probes; suitable for inline or in situ inspection after calibration.	EC and MFL can be high-speed for automated scanning; hysteresis setups are bulkier and slower; MMM allows for rapid surveying but with limited quantification.
**Calibration requirements**	Requires material-specific calibration curves to relate MBN features to stress, hardness, or microstructure; sensitive to magnetizing conditions and sensor lift-off.	Hysteresis and coercivity often referenced to standard samples; MFL/EC need calibration blocks with known defects; MMM usually qualitative, with empirical thresholds.
**Data complexity and processing**	Rich, non-stationary signals; advanced signal processing (RMS, PSD, STFT, wavelets) and increasing use of machine learning to extract features.	Hysteresis: relatively simple scalar descriptors (Hc, Br, µ); MFL/EC: amplitude/phase or image-like maps; MMM: low-bandwidth field curves along scan line.
**Typical applications**	Residual stress and hardness assessment, grinding burn detection, case depth evaluation, microstructure monitoring in steels and ferromagnetic alloys.	MFL: pipeline, wire rope, tank floor inspection; EC: surface cracks, conductivity/thickness; Hysteresis: bulk magnetic property control; MMM: stress concentration and early damage detection in welded or loaded components.

**Table 2:** Star-rated assessment (1–5 stars) of MBN and classical magnetic NDT techniques with respect to key performance criteria, where five stars indicate excellent performance and one-star indicates poor performance. The ratings summarize relative strengths for microstructure and residual stress sensitivity, defect detection, penetration depth, portability, and ease of calibration and interpretation, based on comparative studies of magnetic methods for residual stress and defect evaluation.

Criterion	MBN	Hysteresis/Coercivity	Magnetic Flux Leakage (MFL)	Eddy Current (EC)	Metal Magnetic Memory (MMM)
**Microstructure sensitivity**	★★★★★	★★★★☆	★★☆☆☆	★★☆☆☆	★★☆☆☆
**Residual stress sensitivity**	★★★★★	★★★☆☆	★★☆☆☆	★★☆☆☆	★★★★☆
**Surface/near-surface resolution**	★★★★★	★★☆☆☆	★★★★☆	★★★★☆	★★☆☆☆
**Crack/defect detection**	★★☆☆☆	★★☆☆☆	★★★★★	★★★★★	★★☆☆☆
**Depth of penetration (bulk reach)**	★★☆☆☆	★★★★★	★★★★☆	★★★☆☆	★★★☆☆
**Quantitative capability**	★★★★☆	★★★★☆	★★★☆☆	★★★★☆	★★☆☆☆
**Equipment portability**	★★★★☆	★★☆☆☆	★★★★☆	★★★★★	★★★★★
**Setup and calibration complexity** (*more stars* = *easier*)	★★☆☆☆	★★★☆☆	★★★☆☆	★★★☆☆	★★☆☆☆
**Data interpretation complexity** (*more stars* = *simpler*)	★★☆☆☆	★★★★☆	★★★☆☆	★★★☆☆	★★★★☆

**Table 3:** Operational comparison of magnetic Barkhausen noise (MBN) versus classical NDT techniques, highlighting differences in sensing depth, quantitative analysis potential, and implementation constraints.

Technique	Penetration Depth	Quantitative Capability	Lift-Off Sensitivity	Main Limitation
**MBN**	Frequency-dependent (Skin effect limited); typically 0.01–1.5 mm	High (calibrated stress in MPa; hardness in HV)	High; requires consistent contact or lift-off compensation algorithms	Stochastic nature requires statistical averaging; signals restricted to ferromagnetic materials only
**MFL**	Volumetric (dependent on saturation depth); can detect subsurface defects	Moderate; defect sizing is possible but nonlinear and complex	Moderate; signal amplitude decreases with distance (1/r^3^)	Requires strong magnetic saturation (bulky equipment); limited sensitivity to microstructural changes
**ECT**	Frequency-dependent (standard depth of penetration equation)	High; precise crack depth sizing and conductivity values	Very High; exponential signal decay with lift-off distance	Permeability variations in ferromagnetic steels can mask defect signals (signal-to-noise ratio issues)
**MPI**	Surface and immediate subsurface only	Low; primarily qualitative (Pass/Fail) based on visual indication	N/A (direct contact with particles/suspension required)	High operator dependence; requires post-inspection demagnetization and cleaning; difficult to automate

## Data Availability

The data presented in this study are available on request from the corresponding author.
